# APOE Christchurch enhances a disease-associated microglial response to plaque but suppresses response to tau pathology

**DOI:** 10.1186/s13024-024-00793-x

**Published:** 2025-01-22

**Authors:** Kristine M. Tran, Nellie E. Kwang, Claire A. Butler, Angela Gomez-Arboledas, Shimako Kawauchi, Cassandra Mar, Donna Chao, Rocio A. Barahona, Celia Da Cunha, Kate I. Tsourmas, Zechuan Shi, Shuling Wang, Sherilyn Collins, Amber Walker, Kai-Xuan Shi, Joshua A. Alcantara, Jonathan Neumann, Duc M. Duong, Nicholas T. Seyfried, Andrea J. Tenner, Frank M. LaFerla, Lindsay A. Hohsfield, Vivek Swarup, Grant R. MacGregor, Kim N. Green

**Affiliations:** 1 Department of Neurobiology and Behavior, Charlie Dunlop School of Biological Sciences, University of California, Irvine, CA 92697-4545 USA; 2 Institute for Memory Impairments and Neurological Disorders, University of California, Irvine, CA 92697 USA; 3 Transgenic Mouse Facility, ULAR, Office of Research, University of California, Irvine, CA 92697-2300 USA; 4 ARCProteomics, Atlanta, GA 30322 USA; 5 Goizueta Alzheimer’s Disease Research Center, Emory University School of Medicine, Atlanta, GA 30322 USA; 6 Department of Molecular Biology & Biochemistry, Charlie Dunlop School of Biological Sciences, University of California, Irvine, CA 92697 USA; 7 Department of Pathology and Laboratory Medicine, University of California, Irvine, CA 92697 USA; 8 Center for Complex Biological Systems, University of California, Irvine, CA 92697 USA; 9 Department of Developmental and Cell Biology, Charlie Dunlop School of Biological Sciences, University of California, Irvine, CA 92697 USA

**Keywords:** APOE Christchurch, PS19, 5xFAD, Microglia, DAM, Amyloid, Tau, Resilience

## Abstract

**Background:**

*Apolipoprotein E* ε4 (*APOE4*) is the strongest genetic risk factor for late-onset Alzheimer’s disease (LOAD). A recent case report identified a rare variant in APOE, *APOE3*-R136S (Christchurch), proposed to confer resistance to autosomal dominant Alzheimer’s Disease (AD). However, it remains unclear whether and how this variant exerts its protective effects.

**Methods:**

We introduced the R136S variant into mouse *Apoe* (*ApoeCh*) and investigated its effect on the development of AD-related pathology using the 5xFAD model of amyloidosis and the PS19 model of tauopathy. We used immunohistochemical and biochemical analysis along with single-cell spatial omics and bulk proteomics to explore the impact of the *ApoeCh* variant on AD pathological development and the brain’s response to plaques and tau.

**Results:**

In 5xFAD mice, *ApoeCh* enhances a Disease-Associated Microglia (DAM) phenotype in microglia surrounding plaques, and reduces plaque load, dystrophic neurites, and plasma neurofilament light chain. By contrast, in PS19 mice, *ApoeCh* suppresses the microglial and astrocytic responses to tau-laden neurons and does not reduce tau accumulation or phosphorylation, but partially rescues tau-induced synaptic and myelin loss. We compared how microglia responses differ between the two mouse models to elucidate the distinct DAM signatures induced by *ApoeCh*. We identified upregulation of antigen presentation-related genes in the DAM response in a PS19 compared to a 5xFAD background, suggesting a differential response to amyloid versus tau pathology that is modulated by the presence of *ApoeCh*. Bulk proteomics show upregulated mitochondrial protein abundance with *ApoeCh* in 5xFAD mice, but reductions in mitochondrial and translation associated proteins in PS19 mice.

**Conclusions:**

These findings highlight the ability of the *ApoeCh* variant to modulate microglial responses based on the type of pathology, enhancing DAM reactivity in amyloid models and dampening neuroinflammation to promote protection in tau models. This suggests that the Christchurch variant's protective effects likely involve multiple mechanisms, including changes in receptor binding and microglial programming.

**Graphical Abstract:**

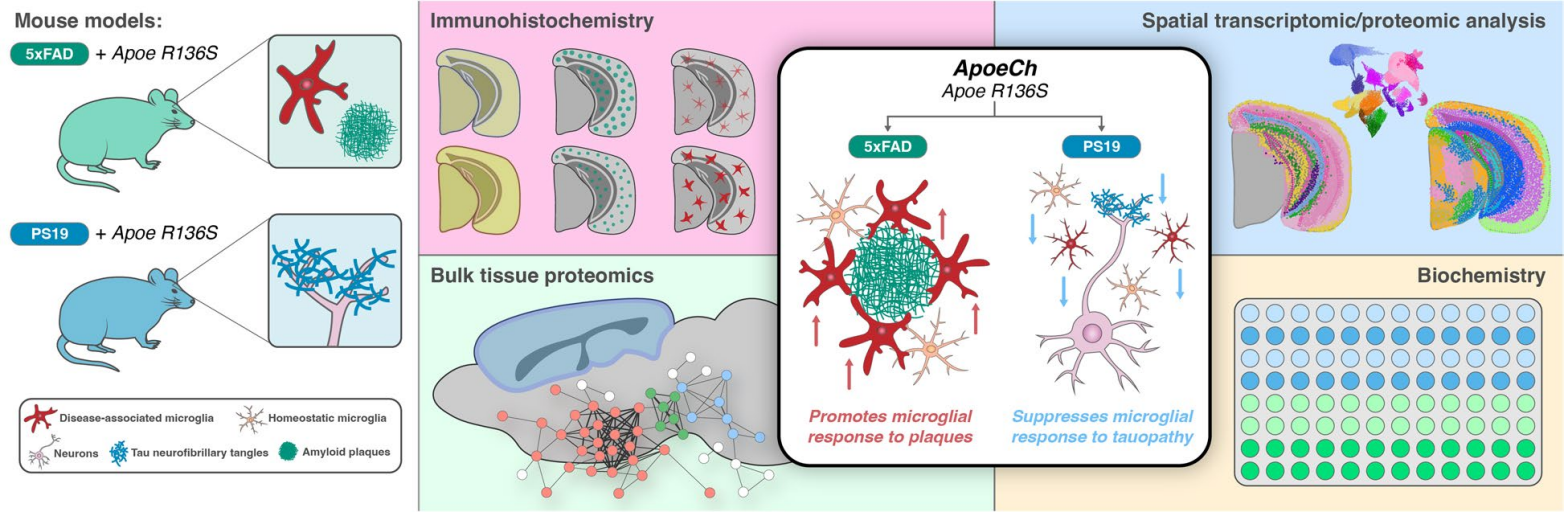

**Supplementary Information:**

The online version contains supplementary material available at 10.1186/s13024-024-00793-x.

## Background

Alzheimer's Disease (AD), the most prevalent neurodegenerative disease, is characterized by a progressive decline in cognitive function accompanied by the development of extracellular neurotoxic amyloid-beta (Aβ) plaques and intracellular neurofibrillary tangles (NFT) [1]. AD can be classified into subtypes based on age of symptom onset: early-onset AD (EOAD) and late-onset AD (LOAD), and disease genetics: genetically determined and sporadic AD. Genetic factors can determine an individual's susceptibility to AD, with rare autosomal dominant AD (ADAD) cases manifesting within families carrying pathogenic variants of *Amyloid Precursor Protein (APP), Presenilin 1 (PSEN1), and Presenilin 2 (PSEN2)* genes [2–7]. Polymorphisms in additional genes have been identified as major risk factors for developing both EOAD and LOAD, such as *Apolipoprotein E* (*APOE)* [8].

**Fig. 1:**
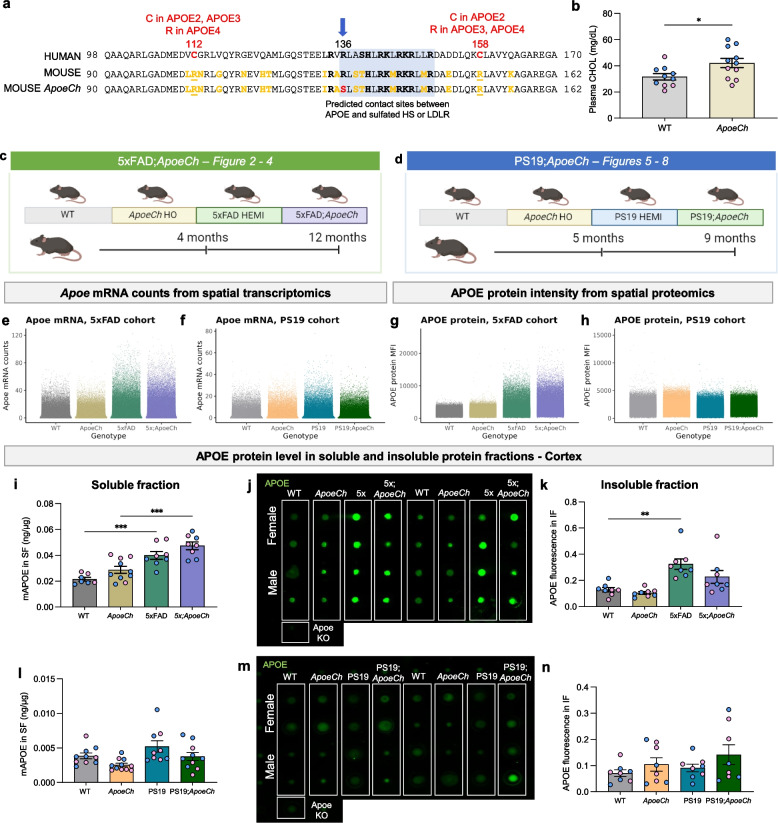
Generation of *ApoeCh* mouse model. **a** Partial amino acid (AA) sequence alignment between human and mouse APOE. The vertical blue arrow denotes the location of the R136S Christchurch variant (*rs121918393*), shown in red in the mouse *ApoeCh* sequence. Mouse has human APOE4 type sequence at positions 112 and 158 (underlined). AA differences between mouse and human APOE4 in this region are highlighted in yellow. In panels b, i, k, l and n, sex of individual animals are denoted by pink (female) or blue (male) circles. **b** Plasma cholesterol in 4 mo WT and *ApoeCh* HO mice (*p* = 0.0274). **c, d** Schematic showing mouse groups and study design of 5xFAD;*ApoeCh* (**c**) and PS19;*ApoeCh* (**d**) cohorts. **e–f** *Apoe* mRNA counts from single-cell spatial transcriptomics in the 5xFAD (**e**) and PS19 (**f**) cohorts. Each point represents one cell. **g-h** APOE protein mean fluorescence intensity (MFI) from single-cell spatial proteomics in the 5xFAD (**g**) and PS19 (**h**) cohorts. Each point represents one cell. **i,l** Quantification of APOE in soluble protein fraction from 5xFAD (**i**) and PS19 cohort animals (**l**) measured via ELISA, normalized to total protein concentration. **j,m** Dot blot analysis of APOE protein in insoluble protein fraction of 5xFAD (**j**) and PS19 (**m**) cohort. **k,n** Quantification of APOE protein in insoluble fraction 5xFAD (**k**) and PS19 (**n**) cohort normalized to sample brain weight. *n* = 2–3 mice/genotype for spatial proteomics. *n* = 3–6 mice/sex/genotype for cortical protein fractions. Data are represented as mean ± SEM. Two-way ANOVA followed by Tukey’s post hoc tests to examine biologically relevant interactions. Statistical significance is denoted by *p < 0.05, **p < 0.01, ***p < 0.001, ****p < 0.0001

APOE is an apolipoprotein secreted by cells in both the central nervous system (CNS) and periphery. APOE is the most abundant apolipoprotein in the brain and is normally expressed at highest levels by astrocytes, and lower levels by microglia, oligodendrocytes, pericytes, endothelial cells, choroid plexus epithelial cells, and neurons. APOE mediates lipid transport and delivery between cell types in the CNS. Mature APOE is a 299 amino acid (AA) protein comprised of three domains – an N-terminal region with four alpha-helices that includes a receptor binding region (AA136-150), a central hinge domain, and a C-terminal domain that can bind lipid (AA244-272). Humans have three common alleles of *APOE* named *APOE-e2* (hereafter referred to as *APOE2)*, *APOE-e3 (APOE3)* and *APOE-e4 (APOE4)*, which encode APOE2, APOE3, and APOE4 proteins respectively. These APOE variants differ in the presence of a cysteine or arginine at positions 112 and 158 within the N-terminal domain, with APOE2 having C112, C158; APOE3 C112, R158 and APOE4 R112, R158. *APOE4* is the most robust genetic risk factor for sporadic LOAD. Homozygous *APOE4* individuals have a 6 to 18-fold increase in AD risk compared to APOE2/3 individuals [9, 10]. By contrast, mouse APOE (mAPOE) shares 70% homology with human APOE and is encoded by one major allele, which resembles the human APOE4 isoform by having arginine at both positions 112 and 158 [11]. Unlike human APOE, mAPOE behaves as a single domain, which affects its lipid-binding capacity. However, the receptor-binding sites (AA136-150) for lipoprotein receptors such as the low-density lipoprotein receptor (LDLR), lipoprotein-related receptor 1 (LRP1), and heparan sulfate proteoglycans (HSPGs) are located in a region conserved between species (Fig. 1a) [11–14].

In 2019, a case report [15] provided striking evidence that APOE is an essential mediator of plaque-induced tau pathology and subsequent neurodegeneration. The proband was heterozygous for the ADAD-associated *PSEN1* E280A variant and was anticipated to develop AD by her forties although she remained cognitively intact until her seventies. Positron emission tomography (PET) imaging revealed significant Aβ plaque deposits in her brain, consistent with carriers of FAD mutations, but a relatively low and atypical burden of neurofibrillary tau tangles and neurodegeneration. Further investigation revealed she was homozygous for the rare Christchurch (R136S) variant in an *APOE3* allelic background (encoding APOE3Ch), suggesting this might account for her cognitive preservation [15]. This key study demonstrated that the presence of plaques can be separated from the development of AD. Understanding the mechanisms by which APOE3Ch prevents the subsequent development of tau pathology and neurodegeneration is essential to stop dementia during the long plaque-laden prodromal phase of AD.

The R136S variant resides in a conserved region of APOE involved in binding to lipoprotein receptors, including LDLR, LRP1, and HSPGs, all implicated in promoting amyloid-β aggregation and neuronal uptake of extracellular tau. Studies indicate that APOE binding is integral to some of these processes [16–20] with differences in APOE isoform-specific receptor binding affinity and lipid interactions affecting neuroinflammation, tau pathology, and amyloid plaque dynamics [21–24]. Collectively, these recent findings underscore the influence of APOE and APOE3Ch on both Aβ and tau pathology, either directly or through downstream effects such as neuroinflammation. The APOE3Ch variant offers a unique opportunity to explore the interplay between Aβ and tau, given its potential association with limited tau development and resistance to cognitive decline despite increased Aβ levels. Mechanistically, APOE may modulate the immune response to plaques, preventing aspects that drive tau pathology, or it may play a pivotal role in inducing and spreading tau pathology and subsequent neurodegeneration. To test these hypotheses, we developed an Apoe*R136S variant in mice, and independently introduced it into both an aggressive mouse model of amyloidosis (5xFAD mice), and of tauopathy (PS19 mice). A potential limitation of this approach is that the Christchurch variant might produce different effects on a mouse APOE background compared to a human APOE3 background due species specific APOE differences. However, a significant advantage of introducing the Christchurch variant into mouse APOE is that this precludes potential confounding effects of studying the human APOE protein in a mouse background. Here, we describe the effects of the mAPOE*R136S (mAPOECh) variant on the development of both plaques and tau pathology, as well as its impact on the brain’s response to these pathologies.

## Methods

### Animals

All experiments involving mice were approved by the UC Irvine Institutional Animal Care and Use Committee and were conducted in compliance with all relevant ethical regulations for animal testing and research. All experiments involving mice comply with the Animal Research: Reporting of in Vivo Experiments (ARRIVE-10) guidelines.

### Environmental conditions

Animals were housed in autoclaved individual ventilated cages (SuperMouse 750, Lab Products, Seaford, DE) containing autoclaved corncob bedding (Envigo 7092BK 1/8″ Teklad, Placentia, CA) and two autoclaved 2″ square cotton nestlets (Ancare, Bellmore, NY) plus a LifeSpan multi-level environmental enrichment platform. Tap water (acidified to pH2.5–3.0 with HCl then autoclaved) and food (LabDiet Mouse Irr 6F; LabDiet, St. Louis, MO) were provided ad libitum. Cages were changed every 2 weeks with a maximum of 5 adult animals per cage. Room temperature was maintained at 72 ± 2°F, with ambient room humidity (average 40–60% RH, range 10—70%). Light cycle was 14 h light / 10 h dark, lights on at 06.30 h and off at 20.30 h.

### Mice

CRISPR/Cas9 endonuclease-mediated genome editing was used to introduce a CGG to TCT missense change resulting in an arginine to serine substitution (R128S) in the mouse apolipoprotein E (*Apoe*) gene. This models the Christchurch R136S missense variant in human *APOE3* (Supp. Figure 1a). Alt-R Crispr RNA (TMF1648 – gcacagaggagatacgggcg) and tracrRNA plus CAS9 protein (HiFi Cas9 nuclease V3, Integrated DNA Technologies (IDT), Coralville, IA) as a ribonucleoprotein (RNP) was electroporated into C57BL/6 J (B6J) zygotes (Jackson Lab stock # 000664, Bar Harbor, ME) along with a ssODN sequence (TMF 1341–5’- ACCATGCTGGGCCAGAGCACAGAGGAGATAAGAGCATCTCTCTCCACACACCTGCGCAAGATGCGCAAGCG —3’). G0 founder animals containing the desired DNA sequence changes were backcrossed with B6J mice and N1 heterozygous mice were sequenced to determine the mutant allele. N1 heterozygous mice were backcrossed with B6J mice to produce N2F1 heterozygotes, which were used to generate animals for subsequent analysis. The formal allele name is *Apoe*^*em1Aduci*^ (Jackson Laboratory stock 039301) For brevity, hereafter we refer to the allele as *ApoeCh*.

5xFAD hemizygous (B6.CgTg(APPSwFlLon,PSEN1*M146L*L286V)6799Vas/Mmjax, Jackson Lab stock # 034848) mice and PS19 hemizygous mice (B6.Cg-Tg(Prnp-MAPT*P301S)PS19Vle/J; Jackson Lab stock # 024841) were obtained from The Jackson Lab. The experimental cohort for 5xFAD hemizygous (HEMI); *ApoeCh* homozygous (HO) and littermate control *ApoeCh* HO mice was derived from natural mating of 5xFAD HEMI; *ApoeCh* HO with *ApoeCh* HO mice. 5xFAD HEMI control mice were generated via in vitro fertilization and embryo transfer to pseudo pregnant surrogate females. Non-transgenic wildtype mice were obtained through natural mating of B6J mice. Similarly, all four groups of the PS19;*ApoeCh* cohort were generated from natural mating of B6J *ApoeCh* heterozygous (HET) mice with (C57BL/6 × C3H)F1 PS19 HEMI mice with subsequent breeding of mixed background PS19 HEMI; *ApoeCh* HET with *ApoeCh* HET. Post-weaning, mice were group-housed with littermates and aged until the designated harvest dates. All animals were bred in the Transgenic Mouse Facility at UCI.

*ApoeCh* genotyping used a common primer set to amplify both the *Apoe* wildtype and the *ApoeCh* alleles (For 5′-GGAGGACACTATGACGGAAGTA-3′ and Rev 5′-TGCCTTGTACACAGCTAGGC-3′). Two fluorophore labeled-hydrolysis probes were used to identify the mouse *Apoe* wildtype amplicon (5’-AGCACAGAGGAGATACGGGCGC −3’—HEX) and the *ApoeCh* amplicon (5’- AGCACAGAGGAGATAAGAGCATC −3’-FAM). The relative fluorescence from each probe was quantified at the end point of PCR cycles to assign genotype using Allelic Discrimination function in Bio-Rad CFX Maestro software (Bio-Rad, Hercules, CA). For 5xFAD genotyping, a hydrolysis probe that hybridizes to the APP(Swe) amplicon was used (For 5′-TGGGTTCAAACAAAGGTGCAA-3′ and Rev 5′-GATGACGATCACTGTCGCTATGAC-3′: APP(Swe) probe 5′-CATTGGACTCATGGTGGGCGGTG-3′). Ct values were normalized to amplification of the *Apob* locus (For 5′-CACGTGGGCTCCAGCATT-3′ and Rev 5′-TCACCAGTCATTTCTGCCTTTG-3′: *ApoB* probe 5′-CCAATGGTCGGGCACTGCTCAA-3′).

Genotyping for the PS19 transgene was performed using two primer sets to amplify both the wildtype (For 5’-CAAATGTTGCTTGTCTGGTG-3’ and Rev 5’-GTCAGTCGAGTGCACAGTTT) and P301S (For 5’-GGCATCTCAGCAATGTCTCC-3’ and Rev 5’- GGTATTAGCCTATGGGGGACAC-3’) using cycling conditions from The Jackson Laboratory (mouse stock # 024841).

### Off-target analysis

Genomic DNA was extracted from mouse tail biopsies using DirectPCR Lysis Reagent (Viagen Biotech, Los Angeles, CA) and Proteinase K (Roche, Indianapolis, IN). Amplification was performed using a Bio-Rad CFX-96 instrument. For each amplicon, a single PCR product was confirmed by capillary electrophoresis (5200 Fragment Analyzer, Agilent, Santa Clara, CA) then subjected to Sanger sequencing (Retrogen, San Diego, CA) and analyzed using SeqMan Ultra 17.4 (DNASTAR, Madison, WI). Potential off-target sites are listed in Supplementary Table 1 while primers for PCR amplification and sequencing of potential off-target sites are listed in Supp. Table 2.

### Behavioral assays

Noldus Ethovision software (Wageningen, Netherlands) was used for video recording and tracking of animal behavior, and subsequent analyses. Detailed protocols are openly accessible via the AD Knowledge Portal (https://adknowledgeportal.synapse.org/). The following behavioral assays were executed following established methodologies as briefly outlined below [25]:

#### Hindlimb clasping

Mice were lifted by the base of their tails while clear of all surrounding objects. They were observed for 10 s and scored based on their hindlimb position as described [26]. Scoring was done by observers blinded to genotype.

#### Elevated plus maze (EPM)

To evaluate anxiety, mice were positioned at the center of an elevated plus maze (arms 6.2 × 75 cm, with 20 cm side walls on two closed arms, elevated 63 cm above the ground) for 5 min. Automated scoring quantified the cumulative time spent by each mouse in the open and closed arms of the maze.

#### Open field (OF)

To assess both motor function and anxiety, mice were placed within a white box (33.7 cm L × 27.3 cm W × 21.6 cm H) for 5 min with movement recorded via video. Videos were subsequently analyzed to quantify the percentage of time spent in the center of the arena, total distance traveled, and speed.

### Plasma lipid measurement

Blood plasma was collected in non-fasted mice immediately before transcardial perfusion and analyzed using the Piccolo® blood chemistry analyzer from Abaxis (Union City, CA) following the manufacturer’s instructions. Plasma was diluted 1:1 with distilled water (ddH_2_O), and 100 µl of the diluted sample loaded onto the Piccolo lipid plus panel plate (#07P0212, Abaxis). Various lipid parameters including total cholesterol (CHOL), high-density lipoprotein (HDL), non-HDL cholesterol (nHDLc), triglycerides, low-density lipoprotein (LDL), and very low-density lipoprotein (vLDL) were analyzed and plotted. Lipid and general chemistry controls (#07P0401, Abaxis) were utilized to verify accuracy and reproducibility of the measurements.

### Tissue preparation for histology

Mice were euthanized by CO_2_ inhalation (5xFAD at 4 and 12 months of age, and PS19 at 5 and 9 months of age), then transcardially perfused with 1X phosphate buffered saline (PBS). In all experiments, the brains were dissected and the hemispheres divided along the midline. One half of each brain was fresh-frozen in dry-ice cooled isopentane at −40 °C for spatial single-cell transcriptomics, while the other half was fixed in 4% paraformaldehyde (PFA) in PBS (Thermo Fisher Scientific, Waltham, MA) for 24 h at 4 °C for immunohistochemical analysis.

### Immunohistochemistry

PFA-fixed brain halves were sectioned into 40 μm coronal slices using a Leica SM2000R freezing microtome, and slices taken for immunohistological analysis (between Bregma −2.78 mm and –3.38 mm for 5xFAD and between Bregma −1.58 mm and −2.7 mm for PS19 according to the Allen Mouse Brain Atlas, Reference Atlas version 1, 2008). One representative brain slice from each mouse within the identical experimental cohort (comprising the same genotype, age, and sex) was subjected to simultaneous staining in the same container as described [25, 27]. The free-floating sections underwent a series of washes, at room temperature unless otherwise stated: three times in 1X phosphate-buffered saline (PBS) for 10 min, 5 min, and 5 min, successively. For AmyloGlo staining, after the PBS washes, free-floating brain slices were washed in 70% ethanol for 5 min, followed by a rinse in deionized water for 2 min, before being immersed in Amylo-Glo RTD Amyloid Plaque Staining Reagent (diluted 1:100 in 0.9% saline solution; TR-200-AG; Biosensis, Thebarton, South Australia) for 10 min. Post-incubation, the sections were subjected to a 5-min wash in 0.9% saline solution and a rinse in deionized water for 15 s before proceeding with a standardized indirect immunohistochemical protocol. Subsequently, the sections were immersed in blocking serum solution (5% normal goat serum with 0.2% Triton X-100 in 1X PBS) for 1 h, followed by overnight incubation at 4 °C in primary antibodies diluted in blocking serum solution. For synaptic staining, free-floating sections were washed three times in 1X PBS then incubated in blocking serum solution overnight at 4 °C, followed by overnight incubation at 4 °C in primary antibodies diluted in blocking serum solution. Finally, the sections were incubated with secondary antibodies for 1 h in the dark followed by 3 washes in 1X PBS, before mounting on microscope slides.

Brain sections were stained using a standard indirect technique [25, 28] with the following primary antibodies against: ionized calcium-binding adapter molecule 1 (IBA1; 1:2000; 019–19,741; Wako, Osaka, Japan), glial fibrillary acidic protein (GFAP; 1:1000; AB134436; Abcam, Cambridge, MA, United States), lysosome-associated membrane protein 1 (LAMP1; 1:200; AB25245, Abcam), CD68 (1:700; 137,002, Biolegend), MC1 (1:100; a gift from P. Davies, Albert Einstein College of Medicine, New York, New York, USA), phospho-Tau Ser202, Thr205 (AT8; 1:500; MN1020; Invitrogen, Waltham, MA, United States), Bassoon (1:250; 75–491; Antibodies Inc, Davis, CA, United States), and Homer1 (1:250; 160,003; Synaptic Systems, Gottingen, Germany).

Brain sections were imaged with a Zeiss Axio Scan Z1 Slidescanner using a 10 × 0.45 NA Plan-Apo objective. Images were also obtained using a 20x, 0.75 NA objective on a Leica TCS SPE-II confocal microscope and analyzed using Bitplane Imaris Software.

For super-resolution imaging, the CA1-SR hippocampal region of WT, *ApoeCh*, PS19 and PS19;*ApoeCh* 9 months old mice were acquired with a Zeiss LSM 900 Airyscan 2 microscope and Zen image acquisition software (Zen Blue, Carl Zeiss, White Plains, NY) with identical conditions. All images were collected using a 63 × 1.4 NA Plan-Apo oil objective and 3 Z-stacks (180 nm step interval, within a depth of 4–6 μm) per mouse/sex/genotype was acquired to obtain a full representation of the CA1-SR. Airyscan processing of all channels and z-stack images was performed in Zen software and Bitplane Imaris Software used for quantification.

### Biochemical analysis of Aβ, tau, and neurofilament light chain

Sample preparation and quantification of Aβ followed established protocols [25, 28]. The hippocampal and cortical regions of each mouse brain hemisphere were micro-dissected and flash-frozen. Samples were pulverized using a Bessman Tissue Pulverizer. For Aβ biochemical analysis, pulverized hippocampal tissue was homogenized in 150µL of Tissue Protein Extraction Reagent (TPER; Life Technologies, Grand Island, NY), while cortical tissue was homogenized in 1000µL/150 mg of TPER. This formulation of TPER, containing 25 mM bicine and 150 mM sodium chloride (pH 7.6), effectively solubilizes proteins within brain tissue post-homogenization. Protease (Roche) and phosphatase inhibitors (Sigma-Aldrich) were added to the homogenized samples, which were then centrifuged at 100,000 g for 1 h at 4 °C to generate TPER-soluble fractions. To generate formic acid fractions, pellets from TPER-soluble fractions were homogenized in 70% formic acid: 75µL for hippocampal tissue or half of the TPER volume used for cortical tissue. Subsequently, samples were again centrifuged at 100,000 g for 1 h at 4 °C. Protein in the insoluble fraction of micro-dissected hippocampal and cortical tissue was normalized to the respective brain region weight, while protein in soluble fractions was normalized to the protein concentration determined via Bradford Protein Assay. Formic acid neutralization buffer (1 M TRIS base, 0.5 M Na_2_HPO_4_, 10% NaN_3_) was used to adjust pH before running ELISAs.

Tau protein extraction from PS19 mice was performed as described [29, 30]. Briefly, microdissected hippocampal and cortical tissues were homogenized in 10uL high salt reassembly buffer (RAB buffer; C752K77; ThermoFisher; 100 mM MES, 1 mM EGTA, 0.5 mM MgSO_4_, 750 mM NaCl, 20 mM NaF, 1 mM Na_3_VO_4_, pH = 7.0) with protease (Roche) and phosphatase inhibitors (Sigma-Aldrich) per 1 mg sample then centrifuged at 50,000 g for 20 min to obtain the supernatant as the RAB-soluble fraction. The pellet was then dissolved in 10uL lysis radioimmunoprecipitation assay (RIPA) buffer with EDTA (CAT# J61529.AP; ThermoFisher; 150 mM NaCl, 50 mM Tris, 0.5% deoxycholic acid, 1% Triton X-100, 0.1% SDS, 5 mM EDTA, 20 mM NaF, 1 mM Na_3_VO_4_, pH 8.0) with protease and phosphatase inhibitor per 1 mg sample and centrifuged at 50,000 g for 20 min to obtain a RIPA-soluble fraction. For formic acid fractions, pellets from RIPA-soluble fractions were homogenized in 70% formic acid: 10µL per 1 mg of tissue. Samples were then centrifuged at 50,000 g for 20 min. Protein in the insoluble fraction of micro-dissected hippocampal and cortical tissue was normalized to the respective brain region weight, while protein in soluble fractions was normalized to the protein concentration determined via Bradford Protein Assay.

#### Meso Scale Discovery (MSD)

Quantitative biochemical analyses of human Aβ soluble and insoluble fraction levels were acquired using the V-PLEX Aβ Peptide Panel 1 (6E10) (K15200G-1; Meso Scale Discovery, Rockville, MD). Tau protein levels were obtained using the Phospho (Thr231)/Total Tau Kit (K15121D; Meso Scale Discovery). Quantitative biochemical analysis of neurofilament-light chain (NfL) in plasma was performed using the R-Plex Human Neurofilament L Assay (K1517XR-2; Meso Scale Discovery).

#### Enzyme Linked-Immuno-Sorbent Assay (ELISA)

Quantification of mouse APOE protein in soluble fraction were acquired using the Mouse Apolipoprotein E (APOE) ELISA Kit (EKN43629-96 T; Biomatik, Wilmington, Delaware,USA).

#### Tau western blots

The cortical soluble protein fraction extracted for Aβ MSD assay were also used for western blotting. Protein concentrations were determined by the Pierce protein assay (22,660; Thermofisher Scientific). Proteins were separated by sodium dodecyl sulfate–polyacrylamide gel electrophoresis (SDS-PAGE) through a 4–12% Bis/Tris gel (Life Technologies) for 2 h at 125 V. Proteins were then transferred to 0.22 μM nitrocellulose membranes (926–31,092, LI-COR) and stained with REVERT 520 total protein stain (TPS, 926–10,021; LI-COR) for 5 min at room temperature. After a brief rinse, the TPS was quantified using ImageStudio software and an Odyssey F imager. TPS values were used for subsequent normalization. Membranes were blocked for 1 h in Intercept® (TBS) blocking solution (927–60,001; LI-COR). Primary antibody combinations and dilutions used include total human Tau (1:1000, A0024; Dako) & pTau (AT8, 1:1000, MN1020; Thermofisher Scientific), total tau (HT7, 1:1000, MN1000; Thermofisher Scientific) & pTau (pThr231, 1:1000, 577,813; Calbiochem). Membranes were incubated with primary antibodies in Intercept® (TBST) antibody diluent (927–65,001; LI-COR) overnight at 4 °C with rocking. The following day, membranes were washed four times (for 4 min each) in TBS with 0.1% Tween-20 (TBST) then incubated with secondary antibodies in Intercept® (TBST) antibody diluent for 1 h at room temperature with rocking. Secondary antibodies and dilutions used for total tau (DAKO) and pTau (AT8) blots were anti-rabbit IRDye® 680RD (1:15,000, 926–68,071; LI-COR) and anti-mouse IRDye® 800CW (1:15,000, 926–32,210; LI-COR). For total tau (HT7) and pTau (pThr231) blots anti- mouse IRDye® 680RD (1:15,000, 926–68,070; LI-COR) and anti-rabbit IRDye® 800CW (1:15,000; 926–32,211; LI-COR) secondaries were used. After four washes of 4 min with TBST, followed by two washes of 2 min with TBS, membranes were imaged using a Odyssey F imager. Quantitative analyses were performed with Empiria Studio Software v2.3 (LI-COR) and fluorescence values normalized to TPS.

#### Dot blots

The cortical formic acid insoluble fractions (IF) extracted for Aβ MSD assay were used for APOE dot blots. For PS19, IF samples were neutralized at 1:15 ratio of sample: neutralization buffer (1 M Tris base, 0.5 M Na_2_HPO_4_, 10% NaN_3_). For 5xFAD, IF samples were neutralized at a ratio of 1:200. Total sample loaded onto microsample filtration apparatus was 200 μl per well/sample onto 0.22 μM nitrocellulose membrane. Once dried, the membrane was blocked in Intercept® (TBS) blocking solution (927–60,001; LI-COR), overnight at 4 °C with rocking. Primary antibodies used: β-amyloid (6e10, 1:1000, 803,001; BioLegend) and Apolipoprotein E (ApoE) (1:1000, AB183596; ABCAM). Membranes were incubated with primary antibodies in Intercept® (TBST) antibody diluent (927–65,001; LI-COR) for 4 h at 4 °C with rocking. Following four washes (4 min each) in TBST, membranes were incubated with secondary antibodies for 1 h at room temperature with rocking. Secondary antibodies used: anti- mouse IRDye® 680RD (1:15,000, 926–68,070; LI-COR) and anti-rabbit IRDye® 800CW (1:15,000; 926–32,211; LI-COR). After four washes of 4 min with TBST, followed by two washes of 2 min with TBS, membranes were imaged using an Odyssey F imager. Quantitative analyses were performed with ImageJ, values expressed as mean fluorescence intensity of each spot (ROI) normalized to pulverized brain weight used to create formic acid fraction and/or β-amyloid fluorescence for 5xFAD samples.

### Imaris quantitative analysis

Confocal images of each brain region were quantified automatically using the spots and surfaces module within Imaris v9.7. Amyloid burden was assessed by measuring the total AmyloGlo + plaque number and volume from 10 × 0.45 NA Zeiss Axio Scan Z1 Slidescanner images. Similarly, volumetric measurements (i.e., AmyloGlo + plaque, IBA1 + microglia volume, etc.) were acquired automatically utilizing the surfaces module with confocal images of each brain region. Quantitative comparisons between experimental groups were carried out in sections stained simultaneously.

For synaptic quantification, the total number of Bassoon or Homer1 puncta was quantified using the spots function of Imaris v9.6. Co-localization of pre- and post- synaptic puncta (Bassoon-Homer1) was determined using the spots function on Imaris and Matlab software to determine the total number of colocalized spots (defined as ≤ 200 nm distance). Results were normalized to the total volume of each image, to correct for any difference in the depth of imaging. Quantitative comparisons between experimental groups were carried out in sections stained simultaneously with the same imaging settings.

### FIJI ImageJ analysis

Prior to analysis, all images were converted to 8-bit gray-scale using Fiji ImageJ. The threshold feature was adjusted and used to distinguish signal from background before percent coverage was measured. To quantify AT8 percent area coverage in the dentate gyrus, the whole FOV of the 20 × confocal images of the dentate gyrus were analyzed. For quantification of AmyloGlo + percent plaque load and CD68 + coverage in the entire brain section, whole brain-stitched 10 × slidescanned images were used with appropriate brain regions outlined to define the region of interest.

### Single-cell spatial transcriptomics and proteomics

Isopentane fresh-frozen brain hemispheres were embedded in optimal cutting temperature (OCT) compound (Tissue-Tek, Sakura Fintek, Torrance, CA), and 10um thick coronal sections were prepared using a cryostat (CM1950, LeicaBiosystems, Deer Park, IL). Six hemibrains were mounted onto each VWR Superfrost Plus microscope slide (Avantor, 48,311–703) and kept at −80 °C until fixation. For both 5xFAD (14 months old, males) and PS19 (9 months old, females and 1 male *ApoeCh*) models, *n* = 3 mice per genotype except for *n* = 2 for PS19;*ApoeCh* (wild-type, *ApoeCh* HO, 5xFAD HEMI or PS19 HEMI, and 5xFAD HEMI; *ApoeCh* HO or PS19 HEMI;*ApoeCh* HO) were used for transcriptomics and proteomics. The same mice were used for both transcriptomics and proteomics. Tissues were processed according to the Nanostring CosMx fresh-frozen slide preparation manual for RNA and protein assays (NanoString University).

#### Slide preparation for spatial transcriptomics

Slides were immersed in 10% neutral buffered formalin (NBF; CAT#15,740) for 2 h at 4 °C, washed three times in 1X PBS (pH 7.4) for 2 min each, then baked at 60 °C for 30 min. Slides were processed as follows: three washes of 1X PBS for 5 min each, 4% sodium dodecyl sulfate (SDS; CAT#AM9822) for 2 min, three washes of 1X PBS for 5 min each, 50% ethanol for 5 min, 70% ethanol for 5 min, and two washes of 100% ethanol for 5 min each, before air drying for 10 min at room temperature. Antigen retrieval was performed in a pressure cooker at 100 °C for 15 min in 1X CosMx Target Retrieval Solution (Nanostring, Seattle, WA). Slides were transferred to DEPC-treated water (CAT#AM9922) and washed for 15 s, incubated in 100% ethanol for 3 min, then air-dried for 30 min. Each slide was incubated with digestion buffer (3 µg/mL Proteinase K in 1X PBS; Nanostring) for tissue permeabilization, then washed twice in 1X PBS for 5 min each. Fiducials for image alignment were diluted to 0.00015% in 2X SSC-T and applied to the slide, then incubated for 5 min. Tissues were then post-fixed with the following washes: 10% NBF for 1 min, two washes of NBF Stop Buffer (0.1 M Tris–Glycine Buffer, CAT#15,740) for 5 min each, and 1 × PBS for 5 min. Next, NHS-Acetate (100 mM; CAT#26,777) mixture was applied to each slide and incubated for 15 min at RT. Slides were washed twice in 2X SSC for 5 min each. Slides were incubated with a modified 1000-plex Mouse Neuro RNA panel (Nanostring) for in situ hybridization along with an rRNA segmentation marker in a hybridization oven at 37 °C for 16–18 h overnight. Following overnight in situ hybridization, slides were washed twice in a stringent wash solution (50% deionized formamide [CAT#AM9342], 2X saline-sodium citrate [SSC; CAT#AM9763]) at 37 °C for 25 min each, then twice in 2X SSC for 2 min each. Slides were incubated in DAPI nuclear stain for 15 min, washed in 1X PBS for 5 min, incubated with GFAP and histone cell segmentation markers for 1 h, then washed three times in 1X PBS for 5 min each. Flow cells were affixed to each slide to create a fluidic channel for imaging, then loaded into the CosMx instrument. Approximately 300 FOVs were selected per slide, capturing hippocampal and cortical regions for each hemibrain section. Slides were imaged for 7 days and data were uploaded to the Nanostring AtoMx platform. Pre-processed data was exported as a Seurat object for further analysis in R 4.3.1.

#### Slide preparation for spatial proteomics

Slides were fixed in 10% NBF for 2 h at 4 °C, washed three times in 1X PBS for 5 min each, baked at 60 °C for 30 min, then washed three times in 1X Tris Buffered Saline with Tween (TBS-T; CAT#J77500.K2) for 5 min each. Antigen retrieval was performed in a pressure cooker at 80 °C in Tris–EDTA buffer (10 mM Tris Base [CAT#10708976001], 1 mM EDTA solution, 0.05% Tween 20, pH 9.0) for 7 min. After antigen retrieval, slides were cooled to RT for 5 min, then washed three times in 1X TBS-T for 5 min each. Slides were then incubated with Buffer W (Nanostring) for 1 h at RT. Next, slides were incubated with CosMx 64-plex protein panel and segmentation markers (GFAP, IBA1, NEUN, and S6) at 4 °C for 16–18 h overnight. After overnight incubation, slides were washed three times in 1X TBS-T for 10 min each, then washed in 1X PBS for 2 min. Fiducials for image alignment were diluted to 0.00005% in 1X TBS-T and applied to the slide, then incubated for 5 min. Slides were washed in 1X PBS for 5 min, incubated in 4% PFA for 15 min, then washed three times in 1X PBS for 5 min each. Slides were incubated in DAPI nuclear stain for 10 min, then washed twice in 1X PBS for 5 min each. Slides were incubated in 100 mM NHS-Acetate and washed in 1X PBS for 5 min. Flow cells were affixed to each slide, then loaded into the CosMx instrument. Approximately 700 FOVs were selected per slide, capturing each full hemibrain section. Slides were imaged for 6 days before the data were uploaded to the Nanostring AtoMx platform for analysis. Data visualization of each exported Seurat object file was performed using R 4.3.1.

#### Spatial transcriptomics data analysis

Spatial transcriptomics datasets were filtered using the AtoMx RNA Quality Control module to flag outlier negative probes (control probes targeting non-existent sequences to quantify non-specific hybridization), lowly-expressing cells, FOVs, and target genes. Datasets were then normalized and scaled using Seurat 5.0.1 *SCTransform* to account for differences in library size across cell types [31]. Principal component analysis (PCA) and uniform manifold approximation and projection (UMAP) analysis were performed to reduce dimensionality and visualize clusters in space. Unsupervised clustering at 1.0 resolution yielded 33 clusters for the 5xFAD dataset and 40 clusters for the PS19 dataset. Clusters were manually annotated based on gene expression and spatial location. Cell proportion plots were generated by first plotting the number of cells in each major cell type and scaling to 1. Normalized percentages for each genotype were calculated by dividing the number of cells in a given cell type-genotype pair by the total number of cells in that genotype, then dividing by the sum of the proportions across the cell type, to account for differences in genotype sample sizes (i.e., in the PS19;*ApoeCh* dataset, *n* = 3/genotype except for *ApoeCh*, where *n* = 2/ genotype). Differential gene expression analysis per cell type between genotypes was performed on scaled expression data using MAST [32] to calculate the average difference, defined as the difference in log-scaled average expression between the two groups for each major cell type. Differential upregulation (DU) and differential downregulation (DD) scores were calculated for all upregulated and downregulated genes, respectively, by summing the product of the negative log-10 adjusted *p*-value and the average difference for each statistically significant gene (i.e., p_adj_ < 0.05). A DU and DD score was calculated for each cluster, then plotted in XY space to visualize spatial differences in gene expression. Directionality of the DU or DD score is reported relative to the first group of the comparison; e.g., in the PS19;*ApoeCh* vs. PS19 comparison, a negative DD score indicates downregulation in the PS19;*ApoeCh* group. However, because PS19;*ApoeCh* brains have very few DAMs, visualizing DD score in PS19;*ApoeCh* brains does not clearly show the impact of *ApoeCh* on DAMs. To visualize DAMs in space, we instead plotted DD score in a PS19 brain (Fig. 8i). Cells with a negative DD score in a PS19;*ApoeCh* brain are conversely upregulated in a PS19 brain. Microglia, astrocyte, and oligodendrocyte glial populations were subsetted and further subclustered for deeper analysis. Data visualizations were generated using ggplot2 3.4.4 [33].

#### Spatial proteomics data analysis

Spatial proteomics data were filtered using the AtoMx Protein Quality Control module to flag unreliable cells based on segmented cell area, negative probe expression, and overly high/low protein expression. Mean fluorescence intensity data were hyperbolic arcsine transformed with the AtoMx Protein Normalization module. Cell types were automatically annotated based on marker gene expression using the CELESTA algorithm [34]. Cell proportions were calculated and percentages normalized as described for spatial transcriptomics data. Protein expression was aggregated and scaled for each cell type to show overall differences in protein expression across the four genotypes. In the PS19;*ApoeCh* dataset, aggregate expression data from the *ApoeCh* genotype (*n* = 2) was multiplied by 1.5 to normalize to the other genotypes (*n* = 3). Data visualizations were generated using ggplot2 [33].

### Single-nucleus RNA-sequencing data analysis

Published and publicly available snRNA-seq data from the *PSEN1*-E280A heterozygote; *APOE3*Ch homozygous individual [35] and PSEN1-E280A controls [36] were downloaded from the NCBI Gene Expression Omnibus (GSE206744; GSE222494). FASTQ files from the *PSEN1*-E280A heterozygote; *APOE3*Ch homozygous individual (*n* = 1) were aligned to the pre-built 10X human GRCh38 reference transcriptome and run through CellRanger to obtain a raw counts matrix. Data from the *PSEN1*-E280A heterozygous controls (*n* = 8) were downloaded directly as a raw counts matrix. To keep controls as representative as possible, three outlier samples were removed. Sample E280A 6 was removed due to carrying one copy of *APOE3*Ch. Samples E280A 7 and E280A 8 were removed due to falling outside of the upper outlier boundary for age at death (> 68), resulting in 5 remaining PSEN1-E280A controls. To match brain regions between batches, only frontal cortex samples were used. Each human dataset contained ~ 38,000 human genes, and was subsetted to contain only genes present in the CosMx 1000-plex mouse neuroscience panel, resulting in 975 overlapping genes between the human snRNA-seq data and the mouse single-cell spatial transcriptomics data. After subsetting, each dataset was processed through the standard Seurat pipeline using SCTransform as described above. Datasets were then merged and integrated using reciprocal PCA to account for batch effects, yielding an integrated dataset of 13,643 cells (Fig. 9h). DGE analysis was performed between the *PSEN1*-E280A;*APOE3*Ch homozygous individual and *PSEN1*-E280A controls for each cell type using MAST (Microglia shown in Fig. 9j). Log-twofold-change values were calculated for the *PSEN1*-E280A;*APOE*Ch vs. *PSEN1*-E280A, as well as for the two mouse comparisons 5xFAD;*ApoeCh* vs. 5xFAD and PS19;*ApoeCh* vs. PS19. Genes with an adjusted *p*-value < 0.05 were considered statistically significant. Genes that were positively correlated (same direction of fold-change; upregulated in red, downregulated in blue) between the three comparisons are shown (Fig. 9k).

### Bulk proteomics

#### Tissue homogenization and protein digestion

Samples were homogenized in 8 M urea lysis buffer (8 M urea, 10 mM Tris, 100 mM NaH_2_PO_4_, pH 8.5) with HALT protease and phosphatase inhibitor cocktail (ThermoFisher) using a Bullet Blender (NextAdvance). The lysates were sonicated for 2 cycles consisting of 5 s of active sonication at 30% amplitude, followed by 15 s on ice. Samples were then centrifuged for 10 min at 8,000 rpm and the supernatant transferred to a new tube. Protein concentration was determined by bicinchoninic acid (BCA) assay (Pierce). For protein digestion, 200 μg of each sample was aliquoted and volumes normalized with additional lysis buffer. Samples were reduced with 5 mM dithiothreitol (DTT) at room temperature for 30 min, followed by 10 mM iodoacetamide (IAA) alkylation in the dark for another 30 min. Lysyl endopeptidase (Wako) at 1:25 (w/w) was added, and digestion allowed to proceed overnight. Samples were then sevenfold diluted with 50 mM ammonium bicarbonate. Trypsin (Promega) was then added at 1:25 (w/w) and digestion proceeded overnight. The peptide solutions were acidified to a final concentration of 1% (vol/vol) formic acid (FA) and 0.1% (vol/vol) trifluoroacetic acid (TFA) and desalted with a 10 mg HLB column (Oasis). Each HLB column was first rinsed with 1 mL of methanol, washed with 1 mL 50% (vol/vol) acetonitrile (ACN), and equilibrated with 2 × 1 mL 0.1% (vol/vol) TFA. The samples were then loaded onto the column and washed with 2 × 1 mL 0.1% (vol/vol) TFA. Elution was performed with 2 volumes of 0.5 mL 50% (vol/vol) ACN. The eluants were aliquoted out to 3 aliquots: 25% (50ug), 65% (130ug) and 10% (20ug). The 25% and 65% aliquots were dried down separately while the 10% aliquot (for each set of mice) was mixed to create a global internal standard. The global internal standard was then aliquoted out to match the 50ug aliquots and dried down.

#### Isobaric Tandem Mass Tag (TMT) peptide labeling

Each sample was re-suspended in 100 mM TEAB buffer (25 μL). The TMT labeling reagents (5 mg) were equilibrated to room temperature, and anhydrous ACN (200μL) was added to each reagent channel. Each channel was gently vortexed for 5 min, and then 5 μL from each TMT channel was transferred to the peptide solutions and allowed to incubate for 1 h at room temperature. The reaction was quenched with 5% (vol/vol) hydroxylamine (2 μl) (Pierce). All channels were then combined and dried by SpeedVac (LabConco) to approximately 100 μL and diluted with 1 mL of 0.1% (vol/vol) TFA, then acidified to a final concentration of 1% (vol/vol) FA and 0.1% (vol/vol) TFA. Labeled peptides were desalted with a 30 mg HLB column (Oasis). Each HLB column was first rinsed with 1 mL of methanol, washed with 1 mL 50% (vol/vol) acetonitrile (ACN), and equilibrated with 2 × 1 mL 0.1% (vol/vol) TFA. The samples were then loaded onto the column and washed with 2 × 1 mL 0.1% (vol/vol) TFA. Elution was performed with 2 volumes of 0.5 mL 50% (vol/vol) ACN. The eluates were then dried to completeness using a SpeedVac.

#### High-pH Off-line fractionation

Dried samples were re-suspended in high pH loading buffer (0.07% vol/vol NH_4_OH, 0.045% vol/vol FA, 2% vol/vol ACN) and loaded onto a Water’s BEH 1.7 um 2.1 mm by 150 mm. A Thermo Vanquish was used to carry out the fractionation. Solvent A consisted of 0.0175% (vol/vol) NH_4_OH, 0.01125% (vol/vol) FA, and 2% (vol/vol) ACN; solvent B consisted of 0.0175% (vol/vol) NH_4_OH, 0.01125% (vol/vol) FA, and 90% (vol/vol) ACN. The sample elution was performed over a 25 min gradient with a flow rate of 0.6 mL/min. A total of 96 individual equal volume fractions were collected across the gradient and subsequently pooled by concatenation into 60 fractions and dried to completeness using a SpeedVac.

#### Liquid chromatography mass spectrometry

All fractions were resuspended in an equal volume of loading buffer (0.1% FA, 0.03% TFA, 1% ACN) and analyzed by liquid chromatography coupled to tandem mass spectrometry. Peptide eluents were separated on Water's CSH column (1.7um resin 150um by 15 cm) by a Vanquish Neo (ThermoFisher Scientific). Buffer A was water with 0.1% (vol/vol) formic acid, and buffer B was 99.9% (vol/vol) acetonitrile in water with 0.1% (vol/vol) formic acid. The gradient was from 3 to 35% solvent B over 16 min followed by column wash and equilibration for a total of 20 min. Peptides were monitored on a Orbitrap Astral spectrometer (ThermoFisher Scientific) fitted with a high-field asymmetric waveform ion mobility spectrometry (FAIMS Pro) ion mobility source (ThermoFisher Scientific). Three compensation voltages (CV) of −35, −50 and −65 were chosen for the FAIMS. Each cycle consisted of one full scan acquisition (MS1) with an m/z range of 400–1500 at 120,000 resolution and standard settings and as many tandem (MS/MS) scans in 1.5 s. The Astral higher energy collision-induced dissociation (HCD) tandem scans were collected at 35% collision energy with an isolation of 0.5 m/z, an AGC setting of 100%, and a maximum injection time of 20 ms. Dynamic exclusion was set to exclude previously sequenced peaks for 30 s within a 5-ppm tolerance window.

#### Database search

All raw files were converted to MZML using proteowizard (version 3.0.24230) and searched using FragPipe (version 22) with MSFragger (version 4.1) using a uniprot curated mouse database supplemented with genotype protein variants. The database contained a total of 17,207 reference protein entries. Default parameters were used: 20 ppm search tolerance on both the MS1 and MS2 levels and 1% FDR for protein level.

#### Protein quantitation and quality control

Protein abundances were normalized by scaling total protein signal within each channel for each specific case sample to the maximum channel-specific total signal. We then used a tunable median polish approach, TAMPOR, to remove technical batch variance in the proteomic data, as previously described [37]. TAMPOR is utilized to remove intra-batch and inter-batch variance while preserving meaningful biological variance in protein abundance values, normalizing to the median of selected samples within batch. This approach is robust to outliers and columns with up to 50% values missing. If a protein had more than 50% samples with missing values, it was removed from the matrix. No imputation of missing values was performed for any cohort. For the current data, TAMPOR leverages the median protein abundance from the pooled Global Internal Standard (GIS) TMT channels as the denominators in both factors to normalize sample-specific protein abundances across the 6 batches of samples. The abundance matrix was then subjected to bootstrap regression to further remove present batch variance.

#### Differential expression analysis

One-way ANOVA with Tukey post-hoc pair-wise tests for significance of the paired groupwise differences across genotype groups was performed in R (version 4.1.2) using an open-source set of R functions documented on https://www.github.com/edammer/parANOVA (parANOVA). Comparisons were performed across various age ranges and genotypes, with Tukey *p* values reported for each individual comparison, along with the log2 fold changes across the comparison groups.

### Statistics

Every reported *n* represents the number of independent biological replicates. The sample sizes are similar to those found in prior studies conducted by MODEL-AD and were not predetermined using statistical methods [25]. Electrophysiology, immunohistochemical, and biochemical data were analyzed using Student’s t-test or two-way ANOVA via Prism v.9 (GraphPad, La Jolla, CA). Tukey’s post-hoc tests were utilized to examine biologically relevant interactions from the two-way ANOVA. For sex-separated data analyses, comparisons with 2 genotypes (i.e. WT, *ApoeCh)*, multiple comparisons between all groups were performed; while those with 4 genotypes (i.e. WT, *ApoeCh*, 5xFAD, and 5xFAD;*ApoeCh),* only comparisons between the genotypes separated by sex were analyzed and reported. Outlier tests were performed via Prism v.9 where relevant and any datapoints removed from the analyses acknowledged in the relevant figure legend. *p ≤ 0.05, **p ≤ 0.01, ***p ≤ 0.001, ****p ≤ 0.0001. Statistical trends were accepted at *p* < 0.10 (# denotes trending significance). Data are presented as raw means and standard error of the mean (SEM).

## Results

### Generation of ApoeCh mice

The three common human variants of APOE (APOE2, APOE3 and APOE4) are distinguished by the presence of either arginine (R) or cysteine (C) at positions 112 and 158. In APOE4, which is now considered causative for AD in a homozygous state [38], arginine residues are found at both sites. Although mice only express one common form of APOE, murine APOE (mAPOE) also contains arginine at positions 112 and 158. However, mAPOE and hAPOE show only partial homology between these sites (Fig. 1a), suggesting there could be differences in how this region of APOE functions between the species. Notably, an HSPG and LDLR binding site in the APOE sequence is conserved in mice (blue boxed area, AA136-150), including arginine 136, which is replaced by serine in the Christchurch (Ch) variant [12, 39]. Given that APOE is a ligand for HSPG and LDLR and is involved in Aβ aggregation and tau uptake, we investigated the effect of introducing the Christchurch variant (R136S) into mAPOE on development of AD-like pathology, independent of human APOE (hAPOE). To accomplish this, we generated *ApoeCh* mice by utilizing CRISPR Cas-9 to introduce an R- > S substitution at amino acid 128 in mAPOE, corresponding to R136S in hAPOE (Fig. 1a). DNA sequence was verified by sequencing of the targeted region (Supp. Figure 1a-b).

We conducted an off-target analysis of other possible CRISPR cut sites that might have been targeted during the generation of the *ApoeCh* variant allele. *ApoeCh* CRISPR G0 founder mice were backcrossed with wild-type B6J animals for three generations before being used to generate homozygous animals for this study, making it unlikely that a mutation caused by an off-target effect of CRISPR/Cas9 would be present on a chromosome other than chromosome 7, i.e., the location of *Apoe* (B6J; Chr7; 48.6 Mb, GRCm39, Ensembl release 108). Potential CRISPR/Cas9 off-target sites with up to four mismatches using crRNA TMF1648 were screened for using Cas-OFFinder (http://www.rgenome.net/cas-offinder/; [40]). Twelve potential off-target sites on mouse chromosome 7 were identified (Supp. Table 1). Two potential off-target sites were within loci encoding lncRNAs, three were within introns, while one was in a 5`UTR, with the remaining six located within intergenic regions. To screen for evidence of CRISPR/Cas9 RNP activity at non-intergenic sites, DNA from a wild-type and homozygous *ApoeCh* mouse was amplified by PCR using the primers listed (Supplementary Table 2) then sequenced across the potential off-target region at six of the loci (Supp. Figure 1d-j). None of the potential off-target sites showed a difference in sequence between WT and homozygous *ApoeCh* mice.

As with the human individual heterozygous for PSEN1*E280A who was homozygous for the *APOE3Ch* variant, and mice with a humanized APOE3*R136S allele, a phenomenon also observed in approximately 10% of *APOE2* homozygotes, *ApoeCh* mice exhibited elevated plasma cholesterol (CHOL), mainly contributed by males (Fig. 1b) [15, 41]. However, we observed no change in plasma triglyceride (TRIG) or very low-density lipid (VLDL) in our mouse model (Supp. Figure 2a, b). Male mice had higher plasma CHOL (Fig. 1b), TRIG (Supp. Figure 2a), and VLDL (Supp. Figure 2b) than females, as previously reported [42].

To investigate whether introduction of the Christchurch variant into mAPOE affects development of AD-like pathologies, while distinguishing between amyloid- and tau-dependent effects, we independently crossed *ApoeCh* mice with the 5xFAD amyloidosis mouse model [25, 43, 44] (Fig. 1c), and the PS19 tauopathy mouse model [45–47] (Fig. 1d; data from Figs. 4, 5, 6, 7 and 8). For crosses with the 5xFAD mouse, we generated four groups: (i) WT, (ii) *ApoeCh* homozygotes (*ApoeCh*), (iii) 5xFAD hemizygotes (5xFAD) and (iv) 5xFAD; *ApoeCh* (5xFAD; *ApoeCh*). Similarly, we generated 4 groups for the tauopathy model: (i) WT, (ii) *ApoeCh,* (iii) PS19 hemizygous (PS19), and (iv) PS19; *ApoeCh* (PS19; *Apoe*Ch). To capture age- and disease progression-dependent changes in phenotypes, we investigated animals at 4 and 12 mo old for 5xFAD cohort and 5 and 9 mo old for the PS19 cohort, corresponding to early and late stages of disease, respectively (Fig. 1c, d).

We verified that the Christchurch variant did not cause a significant change in the level of Apoe mRNA and protein in WT and *ApoeCh* mice from both 5xFAD and PS19 cohorts by quantifying mRNA and protein using spatial transcriptomic and proteomic data from the older cohorts (Fig. 1e-h; described later). Consistent with *Apoe* as a marker for disease-associated microglia (DAM), in the spatial omics data we observed the expected increase in mRNA and protein APOE level with the presence of amyloid but not tau pathology (Fig. 1e, g) [48]. Using ELISA and dot blot we observed no significant difference in mouse APOE in soluble and insoluble protein fractions between WT and *ApoeCh* mice, respectively (Fig. 1i-n). Verification of expected increases in APOE protein in 5xFAD vs WT mice was supported via ELISA and dot blots (Fig. 1i, k).

### ApoeCh variant reduces dense-core Aβ plaque load and plaque-induced neuronal damage

In 5xFAD; *ApoeCh* cohort animals, we observed no significant change in body weight between genotypes at 4 mo, and a small reduction in 5xFAD;*ApoeCh* compared to 5xFAD at 12 mo (Supp. Figure 2c, d). As the 5xFAD mouse model has been reported to exhibit sex-specific differences in phenotype, data separated by sex of significant differences are included in Supp. Figure 3, 4i-x, 6 g-l [25, 49]. Open-field analyses revealed no consistent change in motor function between genotypes at either age (Supp. Figure 2e, h). 5xFAD mice show atypical behavior in the elevated plus maze test, with aged 5xFAD mice preferring the open arms over the closed arms, compared to WT mice. No change was detected in elevated plus maze in the 4-mo groups between any genotype, when pathology is in its early stages in 5xFAD hemizygous mice [25]. At 12 mo, both 5xFAD and 5xFAD;*ApoeCh* mice spent more time in the open arms compared to controls although no significant difference was found between 5 and 5xFAD;*ApoeCh* mice (Supp. Figure 2i, j).

**Fig. 2:**
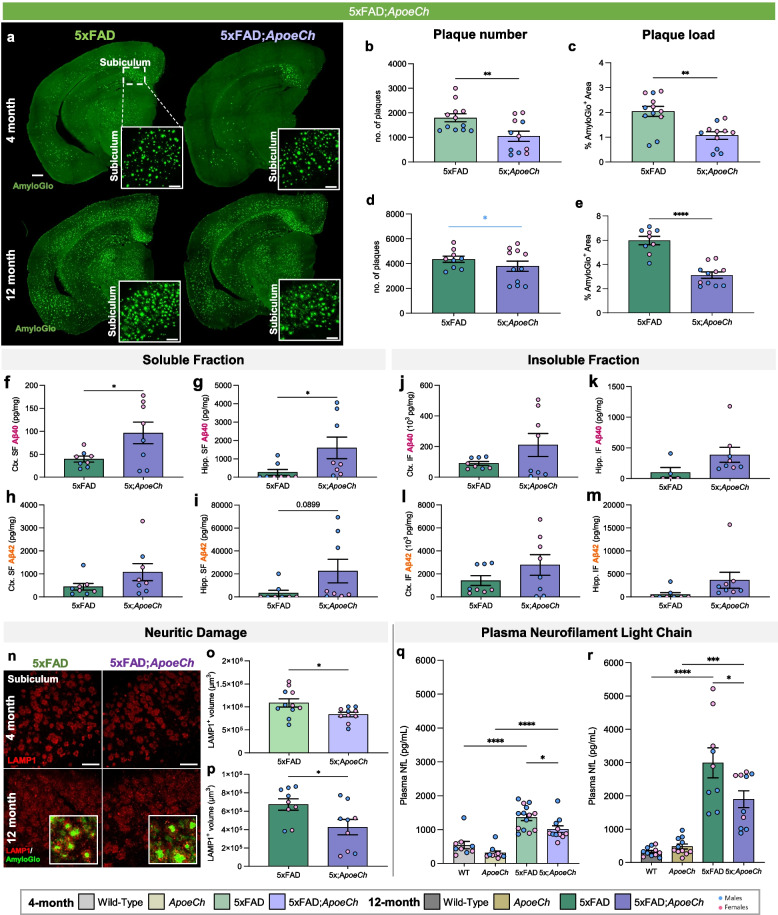
*ApoeCh* variant ameliorates Aβ plaque burden and plaque-induced damage in 5xFAD mice. **a** Representative hemispheric coronal brain images of 4-mo-old (top) and 12-mo-old (bottom) 5xFAD and 5xFAD;*ApoeCh* stained for dense-core plaques using AmyloGlo (green) Scale bar = 500 µm. Insets of 20 × magnification images of the subiculum. Scale bar = 100 µm. **b-e** Quantification of AmyloGlo^+^ plaques (4 month—**b**; 12 month—**d**) and percent AmyloGlo^+^ area coverage (4 month—**c**; 12 month—**e**) of 5xFAD and 5xFAD;*ApoeCh* mice. Blue bar denotes statistical significance only in males. **f**-**m** Quantification of soluble (**f**-**i**) and insoluble (**j**-**m**) Aβ in micro-dissected cortices (**f**, **h**, **j**, **l**) and hippocampi (**g**, **i**, **k**, **m**) of 12 month-old 5xFAD and 5xFAD;*ApoeCh* mice. **n** Representative confocal images of subiculum in 4-mo-old (top) and 12-mon-old (bottom) wild-type, *ApoeCh*, 5xFAD, and 5xFAD;*ApoeCh* mice immunolabeled for LAMP1 (red) for dystrophic neurites quantified in **o** and **p**. Insets show higher magnification images with LAMP1 (red) and AmyloGlo for dense-core plaques (green). Scale bar = 100 µm. Student’s t-test. **q-r** Measurement of plasma NfL in WT, *ApoeCh*, 5xFAD, and 5xFAD;*ApoeCh* mice at 4- (**q**) and 12-mo (**r**). *n* = 4–6 mice/sex/genotype. In panels with graphs, sex of individual animals is denoted by pink (female) or blue (male) circles. Data are represented as mean ± SEM. Two-way ANOVA followed by Tukey’s post hoc tests to examine biologically relevant interactions. Statistical significance is denoted by *p < 0.05, **p < 0.01, ***p < 0.001, ****p < 0.0001

After behavioral testing, brains were extracted and prepared for histological, biochemical, and transcriptomic analyses. AmyloGlo staining revealed a reduced brain-wide dense core plaque load in 5xFAD;*ApoeCh* compared to 5xFAD mice at 4-mo, driven by female mice, and 12 mo of age (Fig. 2a-e, Supp. Figure 3f-i). The overall decrease in plaque load was accompanied by increased soluble and insoluble Aβ40 and Aβ42 in the cortex and hippocampus via MULTI-ARRAY assay (Meso Scale Discovery) at 4 mo of age in 5xFAD;*ApoeCh* compared to 5xFAD mice, which is partially resolved by 12 mo (Fig. 2f-m, Supp. Figure 4). Plaque-induced neuritic damage was assessed by staining for lysosomal-associated membrane protein 1 (LAMP1), which accumulates in dystrophic neurites around plaques [50]. Reduced LAMP1 volume was observed in 5xFAD;*ApoeCh* compared to 5xFAD mice at both time points, with the effect driven mainly by female mice at 4 mo, which is consistent with lowered amyloid plaque load (Fig. 2n-p, Supp. Figure 3j, k). Plasma neurofilament light chain (NfL), an established marker for axonal degeneration that is highly correlated with AD progression [51], was elevated in 5xFAD mice at both 4 and 12 mo compared to WT mice. 5xFAD mice with *ApoeCh* mutation exhibited lower plasma NfL compared to their 5xFAD age-matched controls (Fig. 2q-r, Supp. Figure 3 l, m), suggesting a protective effect of *ApoeCh* on neurodegenerative processes. However, we observed no significant change in synaptic density between 5xFAD;*ApoeCh* compared to 5xFAD mice at 12 mo (Supp. Figure 5). Together, these results show that the presence of the *ApoeCh* variant in the 5xFAD mice reduces plaque pathology as well as amyloid-associated downstream damage.

### Spatial proteomics reveal increased disease associated microglia in response to plaques with ApoeCh

**Fig. 3:**
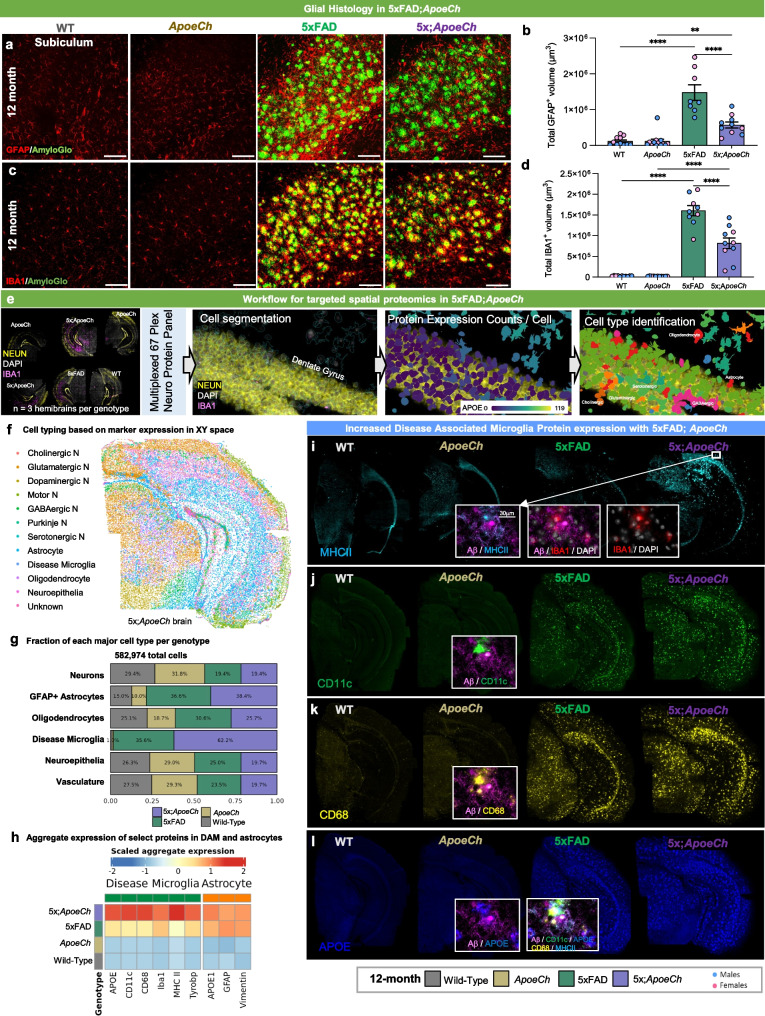
*ApoeCh* increases disease-associated microglia number in response to plaques. **a** Representative confocal images of the subiculum stained for dense-core plaques with AmyloGlo (green) and immunolabeled for GFAP (red, **a**) and IBA1 (red, **c**) of 12-mo-old WT, *ApoeCh*, 5xFAD, and 5xFAD;*ApoeCh* mice. Scale bar = 100 µm. **b, d** Quantification of total volume of GFAP^+^ cells (**b**) and IBA1^+^ cells (**d**). Sex of individual animals is denoted by pink (female) or blue (male) circles. *n* = 4–6 mice/sex/genotype. Data are represented as mean ± SEM. Two-way ANOVA followed by Tukey’s post hoc tests to examine biologically relevant interactions. Statistical significance is denoted by *p < 0.05, **p < 0.01, ***p < 0.001, ****p < 0.0001.**e** Workflow for targeted 67-plex single-cell spatial proteomics. Fields-of-view (FOVs) are first imaged with GFAP, NEUN, RPS6, and IBA1 markers for cell segmentation. Protein abundance is determined by counting the number of fluorescently-labelled oligos in each cell. Cell types are identified with the CELESTA algorithm, which classifies cells based on marker protein expression. **f** Cell types in XY space. CELESTA classifies cells into 12 different cell types, which can then be plotted in space to confirm accurate identification. Non-DAM microglia are unable to be identified using CD11b as a marker; only DAM are shown. **g** Proportions of 5xFAD;*ApoeCh*, 5xFAD, *ApoeCh*, and WT cells for each major cell type. **h** Aggregate expression of the top differentially expressed proteins in DAMs and astrocytes across the four genotypes. **i-l** Immunofluorescence images of MHCII, CD11c, CD68, and APOE for representative brains of WT, *ApoeCh*, 5xFAD, and 5xFAD;*ApoeCh* mice

As APOE proteins are produced primarily by astrocytes and reactive microglia in the CNS, we next examined *ApoeCh*-associated changes in glial cells by histology. Reactive astrocytes, detected by glial fibrillary acidic protein (GFAP), were increased in the subiculum of both 4- and 12-mo-old 5xFAD mice compared to WT. Consistent with lower plaque load, there was decreased astrocyte volume in 5xFAD;*ApoeCh* mice compared to 5xFAD at both ages (Fig. 3a-b; Supp. Figure 6b, g, h). Microglia were detected through immunostaining for ionized calcium binding adaptor molecule 1 (IBA1; Fig. 3c; Supp. Figure 6c) and increased IBA1 + staining was detected in the subiculum of 5xFAD mice at both 4- and 12-mo of age, where abundant plaque load develops, compared to WT. Notably, concomitant with lower plaque load, overall microglial load was significantly reduced at 12 mo in 5xFAD;*ApoeCh* mice compared to age-matched 5xFAD mice in both total IBA1 + volume (Fig. 3d; Supp. Figure 6d, i, j) or IBA1 + cell counts (Supp. Figure 6f, l).

To study the effects of *ApoeCh* on the brain’s response to plaques, we next employed spatial proteomics utilizing a multi-plex 67-protein neuroscience mouse panel (Nanostring CosMx spatial molecular imager; Fig. 3e). This technique maintains the original structure of brain tissue and enables detailed analysis based on protein markers. The panel includes numerous markers for reactive microglia and astrocytes, enabling evaluation of their response to plaques. For each of the four genotypes, data was acquired from three 10 µm coronal slices from 14-mo-old hemibrains, yielding a dataset of 582,974 cells. Cell segmentation was automated using histone, GFAP, and DAPI markers, and cells were categorized by protein expression (Fig. 3e). Examples of cell segmentation are shown in Supp. Figure 7a, illustrating effective cellular identification across dense neuronal areas such as CA1 and dentate gyrus. Plotting and visualization of these cells in XY space confirmed accurate cell identification (Fig. 3f; Supp. Figure 7b). Using this approach, we identified astrocytes, microglia, oligodendrocytes, neuroepithelial cells, and seven neuronal subtypes. We could identify disease-associated microglia (DAM) based on protein expression of CD11c, but not homeostatic microglia. Quantification of cell proportion normalized by total number of cells per genotype revealed a significant increase in the number of DAMs in 5xFAD;*ApoeCh* (~ 62%) compared to 5xFAD (~ 36%) mice (Fig. 3g; Supp. Figure 9e), despite the reduction in plaque load and reduced overall microglial volume (Fig. 2a-d, Fig. 3c-d). Unlike DAM, the number of astrocytes was similar in both 5xFAD and 5xFAD;*ApoeCh* groups compared to WT and *ApoeCh* mice, respectively. We detected no apparent changes in neuronal, neuroepithelial, or vascular populations between WT and 5xFAD mice or 5xFAD and 5xFAD;*ApoeCh* mice (Fig. 3g). These data indicate that introduction of the *ApoeCh* variant into 5xFAD mice increases the proportion of microglia that are identified as DAM.

To explore the microglial response to plaques, we next visualized and measured intensity of several of the DAM markers included in the panel, including MHCII, CD11c, CD68, and APOE (Fig. 3i-l; inserts show localization of these markers around plaques), and found that each marker was upregulated in microglia surrounding plaques. Aggregated expression of these proteins was plotted as a heatmap across the four genotypes, as well as IBA1 and TYROBP, showing significantly increased protein expression of microglial and DAM proteins in 5xFAD;*ApoeCh* compared to 5xFAD brains (Fig. 3h). Plotting protein levels of APOE, GFAP, and VIM in astrocytes shows increases in both 5xFAD genotypes but no difference between 5xFAD;*ApoeCh* and 5xFAD brains (Fig. 3h). Taken together, these data provide evidence that despite reducing plaque load and overall microglial numbers, the presence of *ApoeCh* robustly increases the proportion of DAMs in response to amyloid plaques, as well as upregulates DAM protein expression levels.

### Exploration of the CNS response to amyloid pathology using spatial transcriptomics

**Fig. 4:**
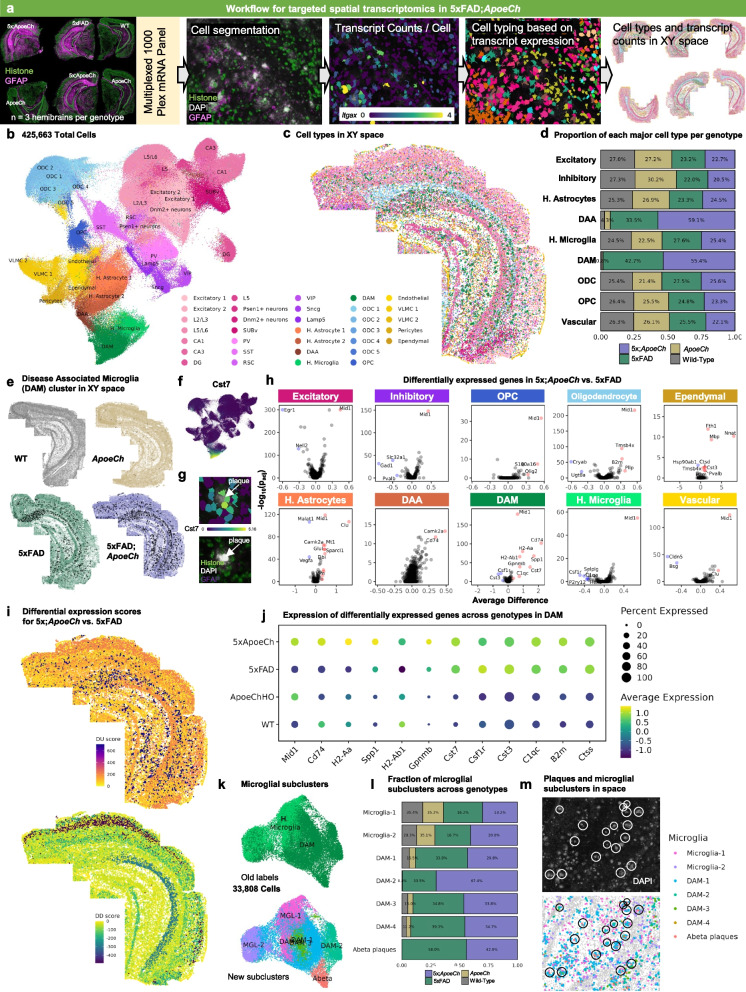
*ApoeCh* enhances microglial response to plaques confirmed by spatial transcriptomics.** a** Workflow for targeted 1000-plex single-cell spatial transcriptomics. FOVs were selected in hippocampus and cortex of each section, then imaged with DNA, rRNA, Histone, and GFAP markers for cell segmentation. Transcript counts for each gene were acquired per cell. **b** UMAP of 425,663 cells across 12 hemibrains (*n* = 3/genotype). Clustering at 1.0 resolution yielded 33 clusters, which were annotated manually based on gene expression and anatomical location in space. **c** 33 clusters plotted in XY space. **d** Proportion of the number of cells in each major cell type, grouped by genotype. Percentages normalized for the total number of cells in each genotype. **e** DAM cluster (black dots) plotted in XY space for each genotype. **f** Feature plot of *Cst7* expression in UMAP space. **g** (top) *Cst7*-expressing cells (i.e., DAMs) surrounding an amyloid-beta plaque. (bottom) DAPI (grey) highlights the same plaque from top panel. Histone marker (green) highlights nucleosomes in cells surrounding the plaque. GFAP + (purple) cell processes surround the plaque.** h** Volcano plots showing DEGs between 5xFAD;*ApoeCh* and 5xFAD among major cell types. **i** Differential upregulation (DU) and differential downregulation (DD) scores for 5xFAD;*ApoeCh* vs. 5xFAD in each cluster plotted in space in a 5xFAD;*ApoeCh* brain. **j** Pseudo-bulk expression of top DEGs between 5xFAD;*ApoeCh* and 5xFAD across genotypes. **k-m** Microglial subclustering analysis. **k** UMAP of 33,808 subsetted microglia. (top) UMAP annotated with old labels from **b**. (bottom) Cells were re-clustered at 0.2 resolution to yield 7 new subclusters. **l** Proportion of the number of cells in each microglial subcluster, grouped by genotype. **m** Microglial subclusters in XY space in a 5xFAD;*ApoeCh* brain. (top) Amyloid-beta dense core plaques, shown by the presence of DAPI + aggregates (grey), circled in white. (bottom) Microglial subclusters plotted in space. Plaques from top panel circled in black

To understand the effects of *ApoeCh* on the brain’s response to plaques, we investigated transcriptional changes in microglia and other CNS cell types using spatial transcriptomics. This method enables single-cell level gene expression analysis in microglia, overcoming limitations associated with single-cell and nucleus RNA-seq, such as technique-induced expression changes [52–56]. Additionally, it provides information on genes at distinct spatial locations within the tissue, facilitating in situ counting and profiling of all cell populations within a brain slice. We conducted our study with single-cell resolution using multiplexed error-robust fluorescence in situ hybridization (MERFISH; Nanostring CosMx spatial molecular imager) and employed a 1000-plex RNA mouse neuroscience panel (Nanostring). Three coronal hemibrain slices per genotype (adjacent to the sections used for spatial proteomics) were processed capturing the hippocampus and cortical regions, resulting in a dataset encompassing 425,663 total cells from 12 brains. Cell segmentation was performed based on histone and ribosome staining (sample cell segmentation examples shown in Supp. Figure 8a), and transcript counts per cell for each of the 1000 genes were then calculated with an average of 754 transcripts per cell for a total of 426,231 cells (workflow shown in Fig. 4a). Cell clustering was performed using a community detection approach on a k-nearest neighbor graph, followed by dimensionality reduction via Uniform Manifold Approximation and Projection (UMAP). Clusters were manually annotated based on gene expression and spatial location. Thirty-three total clusters were identified corresponding to 11 clusters of excitatory neurons, 6 clusters of inhibitory neurons, 3 astrocyte clusters, 6 oligodendrocyte and oligodendrocyte precursor clusters, 2 microglial clusters, as well as 5 endothelial and vascular-related clusters (Fig. 4b). As cell clustering relies purely on gene expression and not spatial coordinates, we verified that cell clustering correctly identified anatomical locations of the cell populations by plotting cells in XY space (Fig. 4c, with all 12 brains shown in Supp. Figure 8b). Cell counts for each of the 33 clusters per genotype were calculated (Supp. Figure 9e), then condensed into major clusters encompassing similar cell types (e.g., ODC1-5 were combined into a broader “oligodendrocyte” cluster). Top gene expression per major cluster are shown in Supp. Figure 9d. The proportion of cells in each major cluster were then plotted per genotype (Fig. 4d). We observe an increased proportion of cells in the disease associated astrocyte (DAA) and disease-associated microglia (DAM) clusters in both 5xFAD and 5xFAD;*ApoeCh* mice, although no change was found among the other cell types. Concordant with spatial proteomics, we observe increases in DAMs in 5xFAD;*ApoeCh* compared to 5xFAD mice, despite their reduced plaque load (Fig. 4d). Plotting the DAM cluster in XY space reveals that DAMs are distributed in 5xFAD and 5xFAD;*ApoeCh* slices in plaque-laden areas (i.e., the subiculum of the hippocampus, throughout the cortex; Fig. 4e). Visualization of cellular expression of the DAM gene *Cst7* (Fig. 4f) shows that *Cst7*-expressing cells cluster around plaques (yellow-green *Cst7* expressing cells surround a white plaque; Fig. 4g), as expected.

To understand the impact of plaque deposition on the different cell types of the CNS, we next conducted differential gene expression (DGE) analysis on 5xFAD and WT brains. Volcano plots show changes in gene expression within each cell population between these two groups (Supp. Figure 10a, c). Consistent with previous sequencing studies, all microglia (combined DAM and microglia clusters, as there are no DAMs in WT and *ApoeCh* genotypes) appear strongly impacted by plaques, as seen by the highest number of DEGs (with 48 DEGs, defined as p_adj_ < 0.05 and absolute average difference > 0.3) between 5xFAD and WT mice. In line with other studies, we observe an upregulation in DAM genes (i.e., *Cst7*, *Apoe*, *Ctsb*, *Ctsd*, *Ctss*, and *Psen1*) and downregulation in homeostatic genes (*P2ry12*, *Tmem119*, and *Csf1r)* in 5xFAD compared to WT mice [48, 57, 58]. In exploring the effects of plaques on neuronal-related genes, we see consistent downregulation of *Egr1*, involved in learning and memory, in 5xFAD compared to WT mice [59]. Our analysis does not detect many genetic changes in grouped excitatory or inhibitory neuronal clusters (4 DEGs in excitatory and 4 in inhibitory; Supp. Figure 10c) but does identify several larger genetic changes when examined in specific neuronal populations (i.e., 23 DEGs in CA1 and 13 DEGs in Subiculum (SUBv)); Supp. Figure 10a). These data illustrate the importance of performing non-aggregate DGE analyses, especially while examining genetic changes within neuronal populations.

Next, we investigated how the presence of *ApoeCh* impacted the brains’ response to plaques. Non-aggregate DGE analysis of 5xFAD;*ApoeCh* vs. 5xFAD brains was performed and revealed changes in gene expression across many of the 33 cell clusters (Supp. Figure 10b), as well as in the aggregate major cell clusters (Fig. 4h), particularly DAM and DAA. To visualize these genetic changes (across all cell types) with spatial resolution, we first generated a DEG score, which represents the magnitude of genetic change between any two animal groups for each cell cluster. DEG score was calculated by summing the product of the average difference in gene expression between two genotypes and the negative log 10 *p*-value for each gene. The calculation gave each cluster a value which is then plotted in XY space (DU score represents the DEG score of all upregulated genes and DD score represents the DEG score of all downregulated genes; Fig. 4i). Focusing on downregulated genes, visualization of the DD score implicates regional specificity of genetic changes induced by introduction of *ApoeCh* into 5xFAD mice within L2/L3 cortical and CA1 neurons (Fig. 4i). Replotting of DG, CA1, and L2/L3 clusters in XY space confirmed correct cell type clustering with high spatial specificity (Supp. Figure 11a). To visualize gross genetic changes across the different genotypes, we next plotted the DEGs from each of these clusters as pseudo-bulked expression values across the four animal groups (Supp. Figure 11b). For L2/L3, several genes were upregulated in 5xFAD mice relative to WT, and then downregulated with 5xFAD;*ApoeCh* (i.e., *Malat1*, *Olfm1*, *Nell2*, and *Snap25*). Many of these genes are involved in cell migration, and axon growth and guidance [60–63]. For CA1, most of the changes appeared in *ApoeCh* mice regardless of 5xFAD transgene array, with similar effects seen in both *ApoeCh* and 5xFAD;*ApoeCh* brains (i.e., *Nell2*, *Penk*, *Mid1*, *Cck*, *Dnm1*, *Nnat*, *Gap43*, and *Atp2b1*). Several of these genes implicate *Apoe*Ch in modulating synaptic function and plasticity.

### Spatial transcriptomics confirms increased disease-associated gene expression in microglia in response to plaques with ApoeCh

APOE is involved in the regulation of DAMs and the microglial response to amyloid [57, 64–66]. Among all the cell types, microglia surrounding plaques (i.e., DAMs) exhibited the greatest differential upregulation (DU) score between 5xFAD;*ApoeCh* and 5xFAD, implicating an important effect of *ApoeCh* on the DAM response to plaques (Fig. 4i). Consistent with the protein expression data, DAM genes were further upregulated in DAMs in 5xFAD:*ApoeCh* (i.e., *Cd74*, *Cst7*, *Gpnmb*, *Spp1*, *H2-Aa*, and *H2-Ab1;* Fig. 4h) while homeostatic microglial genes were further downregulated (i.e., *Csf1r*, *Cst3*). Pseudo-bulk analysis of top DEGs between 5xFAD;*ApoeCh* and 5xFAD in DAMs was performed across the four genotypes, highlighting again that upregulated genes in 5xFAD relative to WT microglia are further upregulated with *ApoeCh* (Fig. 4j). To gain a deeper understanding of microglial populations, we captured all 33,808 microglia and DAM cells across the four groups and reclustered them into seven new subclusters (Fig. 4k). Among the subclustered microglial cells, we identified a subcluster that appeared to represent the epicenter or core of Aβ dense core plaques, as validated by aligning the coordinates of this subcluster with DAPI-positive plaques from CosMx cell segmentation imaging (Fig. 4m). We demonstrated that many “cells” in this cluster were anucleate and did not express histone markers (Supp. Figure 12b). As expected, these presumed plaque core subclusters were only present in 5xFAD;*ApoeCh* and 5xFAD genotypes (Fig. 4l). Compared to other microglial subclusters, this Aβ plaque core subcluster contained a high level of *Iapp*, which encodes the amyloidogenic hormone amylin, along with RNAs for genes involved in microglial activation (i.e., *Cast*, *Pde7b*, *Ltbp1;* Supp. Figure 12c).

Surrounding these plaque core subclusters in space were subclusters DAM-1 and DAM-2, representing plaque-associated microglia. DAM-1 is characterized by high expression of genes involved in antigen presentation and the complement system (i.e., *Ctss*, *Ctsb*, *Ctsd*, *C1qa*, *C1qb*). By contrast, DAM-2 shows high expression of classic DAM marker genes such as *Cst7*, *Trem2*, *Cd74*, *Itgax*, and *Apoe* (Supp. Figure 12c). Cell proportions were calculated for each microglial subcluster across the four genotypes (Fig. 4l). While there was no difference in cell proportions of homeostatic microglia between 5xFAD;*ApoeCh* and 5xFAD, the number of cells (i.e., plaques) in the Aβ plaque core subcluster was reduced in 5xFAD;*ApoeCh* compared to 5xFAD, in line with our IHC findings that the presence of *ApoeCh* reduces plaque load in 5xFAD mice. Furthermore, while numbers of DAM-1 were unchanged between 5xFAD;*ApoeCh* and 5xFAD, DAM-2 (Fig. 4l) was significantly increased in 5xFAD;*ApoeCh* compared to 5xFAD, supporting the increased DAM response in 5xFAD;*ApoeCh* seen in our proteomics data. Together, these data show that the presence of *ApoeCh* in 5xFAD mice upregulates the response of a *Cst7*-expressing DAM population around plaques and reduces plaque load.

### ApoeCh has minimal effects on phosphorylated tau accumulation in the PS19 model

Having shown that the presence of *ApoeCh* promotes a specific DAM response that is neuroprotective against plaque pathology, we next investigated the effect of *ApoeCh* on development of tau pathology. To do so, we crossed *ApoeCh* mice with the PS19 model of tauopathy along with additional crosses for controls, generating four animal groups: (i) WT, (ii) *ApoeCh* HO (*ApoeCh*)*,* (iii) PS19 hemizygous (PS19), and (iv) *ApoeCh* HO; PS19 (PS19;*Apoe*Ch) and aged two cohorts to 5 or 9 mo of age (Fig. 1d). As anticipated, weight loss was observed in both PS19 and *ApoeCh*;PS19 mice at 9 mo of age while no difference was observed between *ApoeCh* and WT mice (Supp. Figure 13a, b). Scoring of hindlimb clasping reflex, a marker of neurodegenerative disease progression, was assessed with impairment recorded in both PS19 and PS19;*ApoeCh* mice at 9 mo of age (Supp. Figure 13c, d) [26]. Open field analyses showed no difference between genotypes at either 5 or 9 mo (Supp. Figure 13e-h), but both PS19 and PS19;*ApoeCh* mice exhibited impairment during elevated plus maze testing at both ages (Supp. Figure 13i, j). These data show that the *ApoeCh* variant was unable to prevent development of motor and anxiety-related impairments in PS19 mice, based on the assays used.

We next measured plasma NfL, as a surrogate for axonal damage, and as expected found a dramatic elevation in NfL in PS19 compared to WT mice at 9 mo of age, although no difference was found between PS19 and PS19;*ApoeCh* mice (Supp. Figure 13 k, l).

**Fig. 5:**
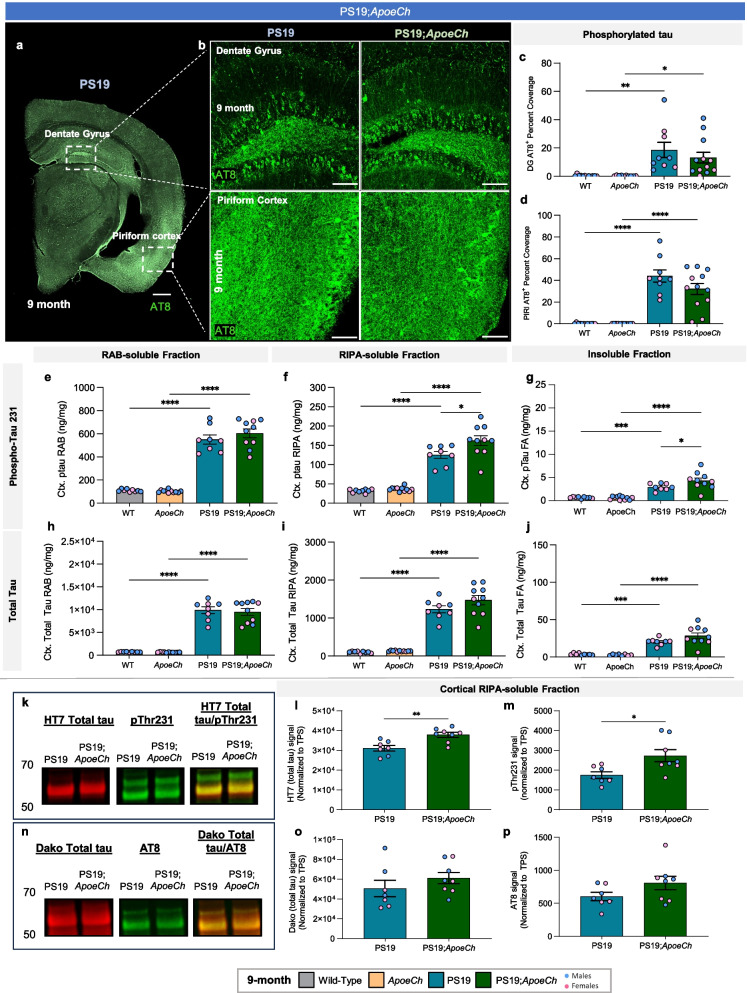
*ApoeCh* has minimal impact on phosphorylated tau accumulation.** a** Representative 10 × image of hemispheric coronal brain image of a PS19 mouse immunolabeled for phosphorylated tau using AT8 (green) highlighting brain regions with high pathological manifestation, dentate gyrus (DG) and piriform cortex (PIRI). Scale bar = 500 µm **b** Representative 20 × confocal images of dentate gyrus (top) and piriform cortex (bottom) from 9-mo old PS19 and PS19;*ApoeCh* mice immunolabeled for AT8 (green). Scale bar = 100 µm. **c-d** Percentage of AT8^+^ area coverage per FOVs of 9 mo-old WT, *ApoeCh*, PS19, and PS19;*ApoeCh* mice in the DG (**c**) and PIRI (**d**). **e-j** Phosphorylated tau T231 (**e**-**g**) and total tau (**h**-**j**) measured via MSD in RAB, RIPA, and formic acid fraction of the micro-dissected cortices of 9-mo-old WT, *ApoeCh*, PS19, and PS19;*ApoeCh* mice. **k** Western blot images of HT7-detected total tau and pThr231 tau in cortical RIPA-soluble fraction of 9-mo PS19 and PS19;*ApoeCh* mice. **l**, **m** HT7 (**l**) and pThr231 signal (**m**) normalized to total protein stain (LPS). **n** Western blot images of total tau detected with Dako antibody and p-tau by AT8 in cortical RIPA-soluble fraction of 9-mo PS19 and PS19;*ApoeCh* mice. **o**, **p** Dako (**o**) and AT8 (**p**) signal normalized to TPS. *n* = 3–6 mice/sex/genotype. In panels with graphs, sex of individual animals is denoted by pink (female) or blue (male) circles. Data are represented as mean ± SEM. Student’s t-test, unpaired. Two-way ANOVA followed by Tukey’s post hoc tests to examine biologically relevant interactions. Statistical significance is denoted by *p < 0.05, **p < 0.01, ***p < 0.001, ****p < 0.0001

Following completion of behavioral testing, brains were extracted and processed for histology, biochemistry, and transcriptomic analyses. Immunostaining for AT8, which detects the phosphorylation of serine 202 threonine 205 of tau, revealed prominent AT8 + pathology in the dentate gyrus (DG) and piriform cortex (PIRI) of in the PS19 mouse model. However, no significant difference was observed between PS19 and PS19;*ApoeCh* mice at both 5 and 9 mo of age (Fig. 5a-d, Supp. Figure 14a-c) [45, 67–69]. Similarly, no difference was observed in numbers of whole brain MC1 + inclusions between PS19 and PS19;*ApoeCh* mice at 9 mo (Supp. Figure 14d-e).

Sequential biochemical extractions in high salt reassembly buffer (RAB), lysis radioimmunoprecipitation assay buffer (RIPA), and 70% formic acid (FA) were performed to obtain extracellular soluble, intracellular detergent soluble, and highly insoluble tau proteins, respectively. Utilizing these brain lysates, we quantified total and phosphorylated tau (Thr231) in 9-mo-old mice via MULTI-ARRAY assay (Meso Scale Discovery). Phospho-tau 231 was increased in all three fractions in both the hippocampus and cortex of PS19;*ApoeCh* mice compared to PS19 mice (Fig. 5e-j; Supp. Figure 14f-h). Total tau was increased in the insoluble hippocampal fraction PS19;*ApoeCh* mice compared to PS19 mice. No significant change was detected between these mice in the cortex or other lysate fractions (Fig. 5h-j; Supp. Figure 14i-k). Additionally, western blot analyses confirmed the increase in pThr231 tau level in the cortical RIPA-soluble fraction in PS19;*ApoeCh* compared to PS19 mice at 9 months while no significant change was detected using AT8, which recapitulates the histology findings (Fig. 5m, p). While we observed an increase in HT7-detected (recognizes human tau AA159-163) total tau level in PS19;*ApoeCh* vs PS19, no difference was observed using DAKO tau antibodies, which recognizes the 4-repeat section of tau (Fig. 5l, o; see Supp. Figure 15 for full blot images). Together, these data indicate that the presence of *ApoeCh* increases the phosphorylation of tau, at Thr231 but does not profoundly alter the accumulation of total tau in PS19 mice.

### ApoeCh suppresses the glial response to tauopathy

**Fig. 6:**
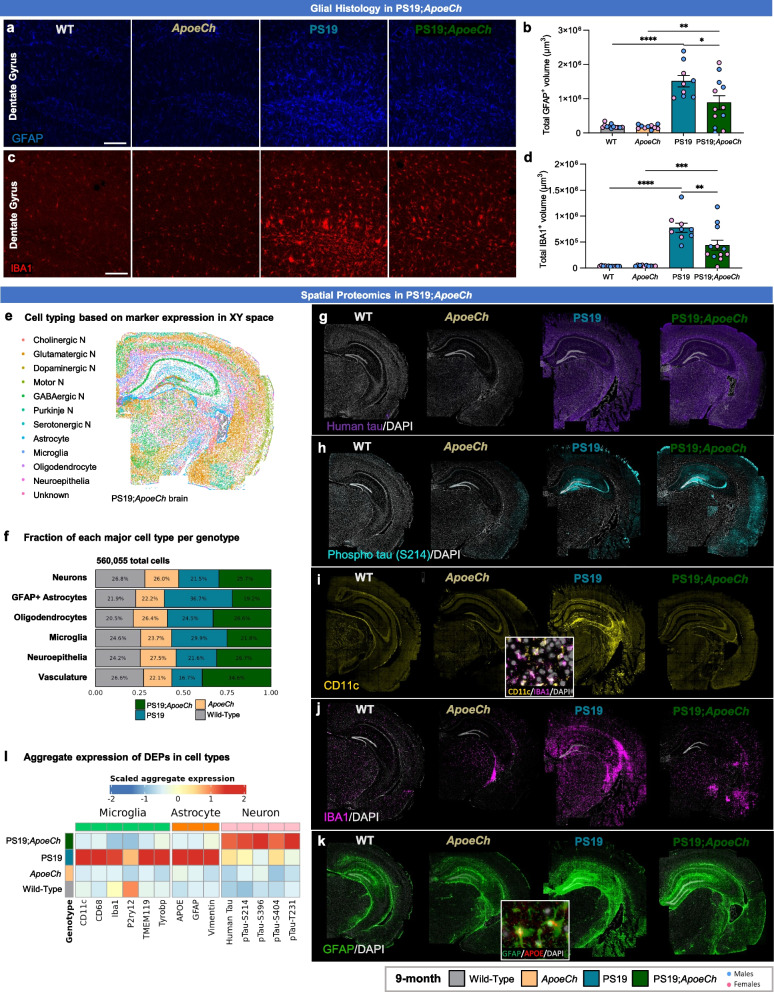
Reduced microglial and astrocytic response to tau induced by *ApoeCh*. **a, c** Representative confocal images of immunostained dentate gyrus for GFAP (blue, a) and IBA1 (red, c) of 9-mo-old WT, *ApoeCh*, PS19, and PS19;*ApoeCh* mice. Scale bar = 100 µm. **b,d** Quantification of total volume of GFAP^+^ cells (**b**) and IBA1^+^ cells (**d**), *n* = 4–6 mice/sex/genotype. Sex of individual animals is denoted by pink (female) or blue (male) circles. Data are represented as mean ± SEM. Two-way ANOVA followed by Tukey’s post hoc tests to examine biologically relevant interactions. Statistical significance is denoted by *p < 0.05, **p < 0.01, ***p < 0.001, ****p < 0.0001. **e** Cell types plotted in XY space. **f** Proportion of the number of cells in each major cell type, grouped by genotype. **g-k** Immunofluorescence images for Human Tau, Phospho-Tau (S214), CD11c, IBA1, and GFAP in representative brains of WT, *ApoeCh*, PS19, and PS19;*ApoeCh* mice. **l** Aggregate expression of differentially expressed proteins (DEPs) in microglia, astrocytes, and neurons across the four genotypes. Expression is normalized for the number of brains in each genotype

Because the introduction of *ApoeCh* into 5xFAD mice promoted the microglial response to plaques, we next explored if this variant would have similar effects on the glial response to tauopathy. We focused our analyses on the 9-mo cohort as no overt pathology was seen at 5-mo (Supp. Figure 16a-e). Immunohistology of the dentate gyrus revealed a dramatic increase in tau-induced astrocytic reactivity visualized by GFAP in PS19 mice that was suppressed in PS19;*ApoeCh* mice (Fig. 6a, b). Similarly, immunostaining for microglia revealed increased total volume of IBA1 + microglia in PS19 mice relative to WT. In PS19;*ApoeCh* mice*,* IBA1 + microglia total volume was decreased*,* despite no change in overall microglia number (Fig. 6c, d, Supp. Figure 16f), suggesting an altered microglial response to the pathology. Thus, despite increases in phosphorylated tau T231, we observed a robust decrease in tau-induced glial responses in the PS19;*ApoeCh* mice. This contrasts with the effects observed of *ApoeCh* on microglia in the presence of plaques.

Given the paradoxical observations that *ApoeCh* promotes the microglial response to plaques, but appears to suppress the microglial response to tauopathy, we used spatial proteomics to further investigate the cellular response. As with the 5xFAD study, we acquired data from 10 µm coronal hemibrain slices (9-mo-old mouse samples, *n* = 3 for WT, PS19 and PS19;*ApoeCh* brains, and *n* = 2 for *ApoeCh*) yielding a dataset of 560,055 cells. Cell segmentation was automated using histone, GFAP, and DAPI markers (Supp. Figure 17a), then automatically assigned to cell types using marker proteins. Plotting these cell types in XY space confirmed accurate cell identification (Fig. 6e; all imaged brains shown in Supp. Figure 17b). Here, the “microglia” category includes both homeostatic and disease-associated microglia. Quantification of cell proportions between genotypes revealed that microglia and astrocyte populations were expanded in PS19 brains, but unchanged in WT, *ApoeCh*, and PS19;*ApoeCh* brains. No change was observed in the proportions of neurons overall across the four genotypes suggesting no neuronal loss is observed in PS19 mice at this age (Fig. 6f).

Next, we plotted the aggregate expression of select proteins across microglia, astrocyte, and neuronal populations (Fig. 6l). Sample images from the proteomic analyses are shown for neuronal (human Tau and p-Tau S214), microglial (CD11c and IBA1), and astrocyte (GFAP) markers (Fig. 6g-k). In microglia, we observed a significant reduction in DAM-related proteins (i.e., CD11c, CD68, TYROBP) in PS19;*ApoeCh* compared to PS19, indicating that microglia have a reduced response to tauopathy in the presence of *ApoeCh*. The reduction in CD68 whole-brain coverage in 5xFAD;*ApoeCh* vs 5xFAD mice was independently supported via immunostaining for CD68 (Supp. Figure 16g-h). Similarly, we observed that astrocyte-related proteins GFAP, APOE, and VIM are reduced in PS19;*ApoeCh* compared to PS19, supporting results of histology (Fig. 6a, b). Given the biochemical findings that levels of phosphorylated tau (pTau) were altered in hippocampal and cortical tissue lysates, we next focused on pTau expression in neurons. Aggregated protein expression data in neurons showed increased pTau-T231 in PS19;*ApoeCh* brains compared to PS19, consistent with our biochemical data (Fig. 6l). Additionally, we identified that aggregate expression of other forms of pTau (i.e., pTau-S396, pTau-S214, and pTau-S404) were also increased in PS19;*ApoeCh* brains compared to PS19 (Fig. 6l). These findings show that despite elevated phosphorylated tau accumulation in neurons, there is a marked suppression of both microglial and astrocytic responses in the presence of *ApoeCh*.

### Spatial transcriptomics reveals PS19 transgene-induced changes in gene expression in glial populations are prevented by ApoeCh

**Fig. 7:**
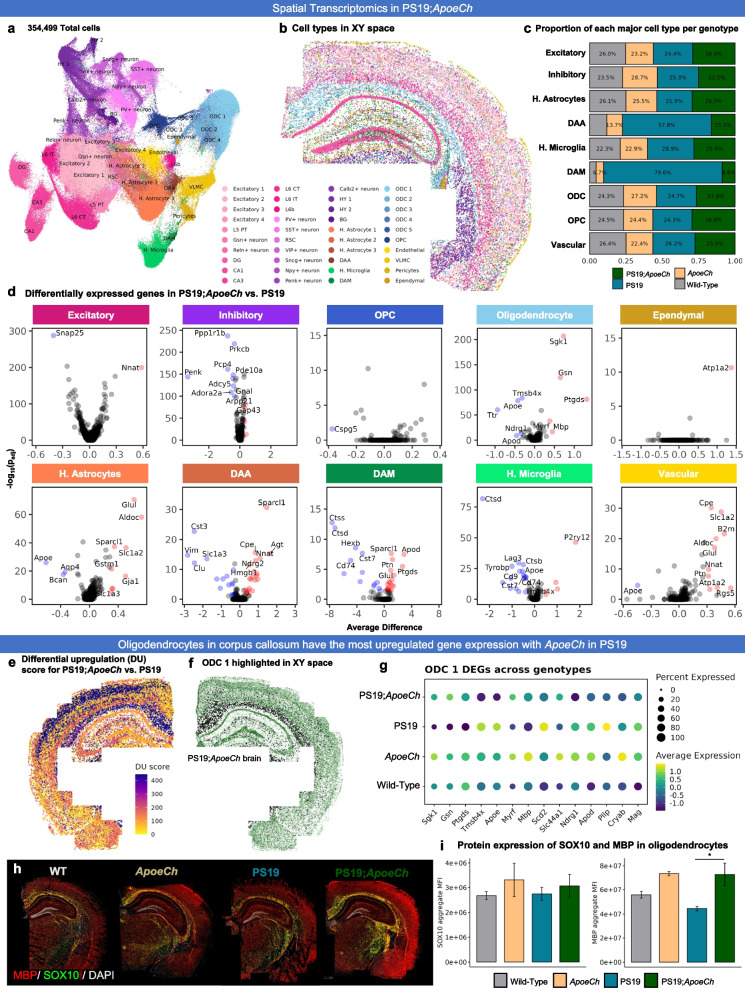
*ApoeCh* prevents tau-induced myelin loss via changes in oligodendrocyte transcriptomics. **a** UMAP of 354,499 cells across 11 hemibrains (*n* = 3/genotype, except *n* = 2 for *ApoeCh*). Clustering at 1.0 resolution gave rise to 40 clusters, which were then manually annotated based on gene expression and anatomical location in space. **b** 40 clusters plotted in XY space. Common legend between **a**, **b**. **c** Proportion of the number of cells in each major cell type, grouped by genotype. Percentages were normalized for the total number of cells in each genotype (i.e., to account for differences in the number of samples per genotype). **d** Volcano plots of DEGs between PS19;*ApoeCh* and PS19 for each major cell type. **e** DU score for PS19;*ApoeCh* vs. PS19 in a PS19;*ApoeCh* brain. **f** ODC 1 cluster highlighted (black) in XY space in a PS19;*ApoeCh* brain. **g** Pseudo-bulk expression of the top DEGs between PS19;*ApoeCh* and PS19 in the ODC 1 cluster across all four genotypes. **h** Immunofluorescence images of MBP (red) and SOX10 (green) in representative brains from each genotype from spatial proteomics dataset. DAPI nuclear stain shown in light grey. **i** Bar plots showing mean fluorescent intensity of SOX10 and MBP in oligodendrocytes, aggregated per sample. Error bars indicate the standard error of the mean (SEM). Adjusted *p*-value for the comparison between PS19;*ApoeCh* and PS19 for MBP was obtained using two-way ANOVA followed by Tukey’s HSD post-hoc test to determine specific pairwise differences. * p_adj_ < 0.05

To further interrogate the role of *ApoeCh* in the tauopathy murine brain, we performed single-cell spatial transcriptomics analysis, imaging coronal hemibrain slices adjacent to the sections used for spatial proteomics, with data acquired from the cortex and hippocampus. 354,499 cells were processed, clustered, and manually annotated based on gene expression and spatial location to yield a total of 40 clusters, corresponding to 13 clusters of excitatory neurons, 11 inhibitory neurons, 4 astrocytes, 6 oligodendrocytes and oligodendrocyte precursors, 2 microglia, and 4 endothelial and vascular-related clusters (Fig. 7a). Sample cell segmentation is shown in Supp. Figure 18a. Plotting clusters in space again confirmed accurate cell typing (Fig. 7b, and all imaged brains are shown in Supp. Figure 18b). Top marker genes per major cell type were calculated to validate cell annotation (Supp. Figure 19d), and cell numbers per cluster per genotype are plotted in Supp. Figure 19e. Calculating the proportions of cells within each major cell type normalized by genotype revealed a marked decrease in the populations of DAAs and DAMs in PS19;*ApoeCh* mice compared to PS19, supporting the reduced glial response seen in PS19;*ApoeCh* from IHC and spatial proteomics data (Fig. 7c). Viewing the UMAP split by genotype similarly shows a prominent DAM cluster in PS19 mice that is absent in the other three genotypes (Supp. Figure 19c).

To understand how the presence of tau pathology affects the 40 cell clusters identified in the brain, we performed DGE analysis between PS19 and WT mice. Volcano plots for each of the cell types are shown in Supp. Figure 20a. Because DAAs and DAMs were not present in WT mice, these cells were grouped into “All astrocytes” and “All microglia” clusters for the PS19 vs. WT comparison. To further simplify interpretation of results, cells from individual clusters were grouped into major cell types and volcano plots generated for each major cell type (Supp. Figure 20c). Overall, we observed large numbers of significantly upregulated genes in inhibitory neurons (5 upregulated DEGs, defined as p_adj_ < 0.05 and average difference > 0.3), astrocytes (14 upregulated), and microglia (27 upregulated), while oligodendrocytes exhibited both up- (8 upregulated) and down-regulated (4 downregulated) genes. Of all clusters, we detected the most differences in microglia (32 total DEGs) between PS19 and WT brains, with PS19 showing an upregulation in inflammatory-related genes such as *Cd74*, *C1qa*, *C1qb*, *C1qc*, *Ctss*, *Ctsd*, *Cst7*, as well as *Apoe*. Astrocytes in PS19 mice also showed upregulation in reactive astrocytic markers *Vim*, *Clu*, *Aqp4*, and *Ctsb* compared to WT mice (Supp. Figure 20a). When grouped together, excitatory neurons showed few significant changes, and inhibitory neurons showed upregulation of *Gsk3b*, a kinase implicated in tau phosphorylation, along with *Penk*, *Pcp4*, *Ppp1r1b*, and *Adora2a* (Supp. Figure 20c). However, when analyzed separately, DGE analysis revealed significant changes in many neuronal populations, notably *Lamp5* + , *Calb2* + , L6b, and hypothalamic (HY) neurons, again reinforcing the utility of analyzing discrete neuronal clusters rather than aggregated data analysis.

Next, we performed DGE analysis between PS19;*ApoeCh* and PS19 mice to investigate the brains’ response to tau laden neurons in the presence of *ApoeCh*. Volcano plots for all 40 cell clusters are shown in Supp. Figure 20b, and broad collapsed cell clusters shown in Fig. 7d. PS19;*ApoeCh* mice exhibit downregulated genes in inhibitory neurons (9 downregulated), DAM (13 downregulated), and microglia (17 downregulated), while genes in astrocytes (9 upregulated), and vascular cells (10 upregulated) were mostly upregulated compared to PS19 mice. In PS19;*ApoeCh* mice, oligodendrocytes (5 upregulated and 5 downregulated) and DAA (22 upregulated and 15 downregulated) cells exhibit gene expression changes in the form of both up- and down-regulated genes. DGE analysis of DAMs revealed significant downregulation of genes related to lysosomal function, including *Ctss*, *Ctsd*, *Cst3*, and *Hexb* (Fig. 7d). Notably, many of the genes that were upregulated in microglia for the PS19 vs. WT comparison (i,e., *Ctss*, *Ctsd*, *C1qa*, *C1qb*, *C1qc*, *Cst3*, *Apoe*, *Tyrobp*) were instead downregulated in the PS19;*ApoeCh* vs. PS19 comparison. A similar reciprocal pattern was observed in astrocytes, neurons, and oligodendrocytes. For example, genes that were upregulated in astrocytes in the PS19 vs. WT comparison, including reactive astrocytic markers *Vim* and *Clu*, were instead downregulated in the PS19;*ApoeCh* vs. PS19 comparison. These data provide evidence that some of the genetic changes induced in different cell populations by the PS19 transgene, such as those associated with glial activation, are suppressed by the presence of the *ApoeCh* variant.

### PS19-induced changes in oligodendrocyte gene expression in the corpus callosum and level of MBP are prevented by ApoeCh

To visualize patterns of DGE in space, we plotted DU scores between PS19;*ApoeCh* and PS19. Most upregulation in genes in PS19;*ApoeCh* vs. PS19 mice occurs in white matter tracts (ODC 1) and L2/L3 neurons in cortex (Excitatory 1; Fig. 7e). Cluster ODC 1 is highlighted in a PS19;*ApoeCh* brain in Fig. 7f and localizes to the corpus callosum. We performed pseudo-bulk analysis of the top DEGs between PS19;*ApoeCh* and PS19 mice in the ODC 1 cluster, which are displayed as a dot plot across the four genotypes. These data reveal that several ODC 1 cluster genes altered by PS19 (compared to WT) show a reverse in directionality of expression upon introduction of the *ApoeCh* variant. These genes include *Sgk1*, *Gsn*, and *Ptgds* (downregulated in PS19 compared to WT and upregulated in PS19;*ApoeCh* compared to PS19)*, Tmsb4x*, *Apoe*, *Scd2*, and *Pllp* (upregulated in PS19 compared to WT and reduced in PS19;*ApoeCh*). We also found several ODC 1 genes were upregulated by *ApoeCh,* independent of PS19 background, including *Mbp*, *Myrf* and *Slc44a1*, of which *Mbp* was restored to WT levels in PS19;*ApoeCh* mice. Given the restoration of ODC 1 *Mbp,* and other oligodendrocyte-associated genes, we used the spatial proteomics dataset to analyze the level of myelin basic protein (MBP) and SOX10 in oligodendrocytes. Consistent with gene expression data, aggregate MBP protein expression in oligodendrocytes was higher in *PS19;ApoeCh* compared to PS19 mice (Fig. 7h, i). Conversely, levels of SOX10, a transcription factor that plays a role in oligodendrocyte differentiation, were not significantly altered across genotypes. Together, these data indicate that *ApoeCh* induces changes in myelination (measured by MBP) without altering oligodendrocyte differentiation (measured by SOX10), in response to tau pathology.

### PS19-induced changes in CA1 neuron gene expression and CA1 synaptic densities are prevented with ApoeCh

**Fig. 8:**
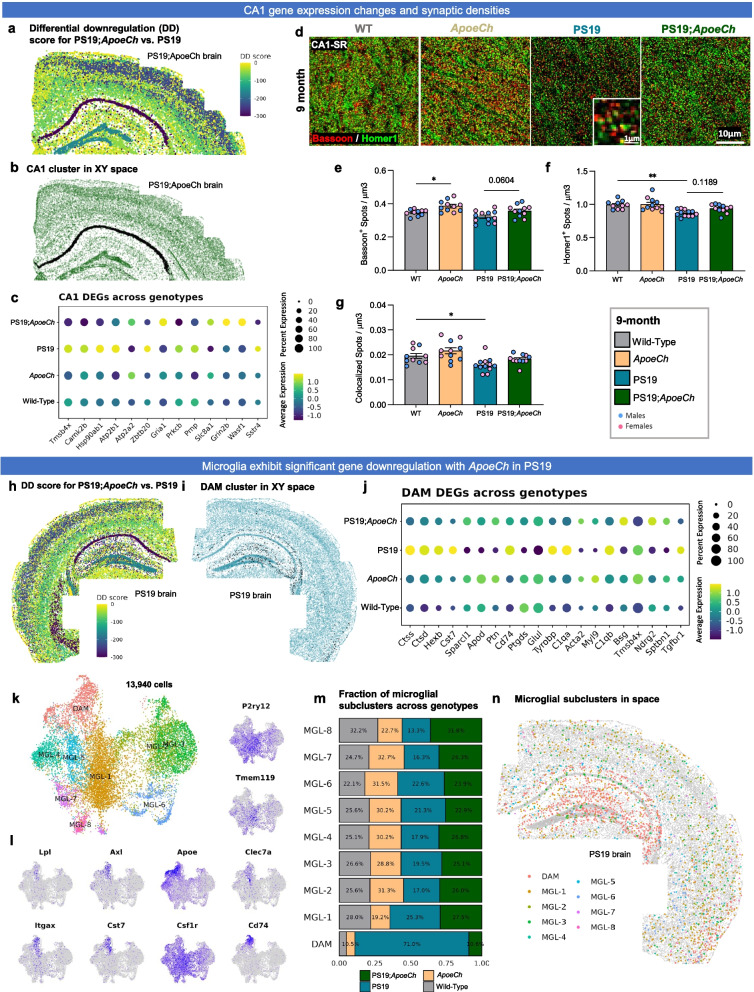
*ApoeCh* suppresses microglial response to tau and partially suppresses tau-induced synaptic loss in the PS19 mouse model. **a** DD score for PS19;ApoeCh vs. PS19 in a PS19;*ApoeCh* brain**. b** CA1 cluster highlighted (black) in XY space in a PS19;*ApoeCh* brain. **c** Pseudo-bulk expression of the top DEGs between PS19;*ApoeCh* and PS19 in the CA1 cluster across the four genotypes. **d** Representative super-resolution images of Bassoon and Homer1 synaptic markers for WT, *ApoeCh*, PS19, and PS19;*ApoeCh* mice at 9 mo of age. Scale bar = 10 μm. Insert scale bar = 1 µm **e–g** Quantification of Bassoon + spots per μm^3^, Homer1 + spots per µm^3^ and colocalized Bassoon + /Homer1 + synaptic spots per µm^3^ showing decreased synaptic puncta in PS19 mice compared to WT mice. Three images per mouse and *n* = 5–6 mice/sex/genotype. Data are represented as mean ± SEM. Two-Way ANOVA followed by Tukey’s post hoc test to examine biologically relevant interactions. *p < 0.05, **p < 0.001. **h** DD score for PS19;*ApoeCh* vs. PS19 in a PS19 brain. **i** DAM cluster highlighted (black) in XY space in a PS19 brain. **j** Pseudo-bulk expression of the top 20 DEGs between PS19;*ApoeCh* and PS19 in the DAM cluster across the four genotypes. **k-n** Microglial sub-clustering analysis. **k** UMAP of 13,940 microglial cells in space. Clustering at 0.4 resolution yielded 9 subclusters, excluding the smallest subcluster that had fewer than 100 cells. **l** Feature plots of common microglial and DAM marker genes in UMAP space. **m** Proportion of the number of cells in each microglial subcluster, grouped by genotype. Proportions are normalized for the total number of microglia in each genotype. **n** Microglial subclusters plotted in XY space in a PS19 brain

Having shown and explored the changes associated with differential gene upregulation, we next plotted differential downregulation scores between PS19;*ApoeCh* and PS19 (Fig. 8a). These data highlight DAM, microglia, DAA, and CA1 as the clusters with the most downregulated genes in PS19;*ApoeCh* compared to PS19 mice (DD scores were plotted in a PS19;*ApoeCh* brain, which has limited DAM). We focused initially on the changes in the CA1 cluster, which localized spatially exclusively in the CA1 (Fig. 8b). Pseudo-bulk analysis was performed for the top DEGs in the CA1 cluster across the four genotypes (Fig. 8c). These data show that many genes that were upregulated in PS19 relative to WT mice (i.e., *Tmsb4x*, *Camk2b*, *Hsp90ab1*, *Zbtb20*, *Atp2b1*, *Sstr4*) were subsequently downregulated in PS19;*ApoeCh* compared to PS19 mice. Conversely, genes that were downregulated in PS19 mice relative to WT (i.e., *Atp2a2*, *Slc8a1)*, genes involved in calcium homeostasis and neuronal signaling, were upregulated in PS19;*ApoeCh* compared to PS19 mice. There were also several genes (i.e., *Gria1*, *Grin2b*, *Wasf1*), involved in synaptic plasticity, synaptic transmission, and long-term potentiation that showed low expression in WT, *ApoeCh*, and PS19, but which were upregulated in PS19;*ApoeCh* (Fig. 8c) [70–73].

To examine the functional changes occurring in CA1, we immunostained for presynaptic marker Bassoon and postsynaptic marker Homer1 in 9-mo-old mouse brains and imaged synaptic puncta via super resolution microscopy (Fig. 8d). Interestingly, the *ApoeCh* variant increased Bassoon presynaptic puncta independently of PS19 transgene (Fig. 8e). Significant reductions in both Homer1 postsynaptic puncta and colocalized Homer-Bassoon pre- and post-synaptic puncta were observed in PS19 mice compared to WT, but no reduction was observed between *ApoeCh* and PS19;*ApoeCh* brains (Fig. 8f, g), suggesting that the *ApoeCh* variant can partially suppress the synaptic loss seen in the PS19 mouse model.

### Spatial transcriptomics reveal a suppressed transition to disease-associated microglia in response to tauopathy with ApoeCh

DAMs and microglia had the greatest differential downregulation score in the PS19;*ApoeCh* vs. PS19 comparison (Fig. 8h; −336.2 for DAM, −378.5 for microglia, −279.8 for CA1). We plotted DAMs in a PS19 brain (Fig. 8i), showing that these cells localize to areas of the hippocampus and piriform cortex where tau pathology is seen. We performed pseudo-bulk analysis of the top 20 DEGs between PS19;*ApoeCh* and PS19 DAMs and plotted their expression levels across the four genotypes in DAMs (Fig. 8j). As expected, many genes upregulated in PS19 mice relative to WT (i.e., *Ctss, Ctsd, Hexb, Cst7, Cd74, Tyrobp, C1qa, C1qb,* and *Tgfbr1*) are not upregulated in PS19;*ApoeCh* mice. Similarly, several genes downregulated in PS19 compared to WT (i.e., *Sparcl1*, *Apod*, *Ptn*, *Ptgds*, *Glul*, *Acta2*, *Myl9*, *Bsg*, *Ndrg2*, *Sptbn1*) are expressed at WT level in PS19;*ApoeCh*.

To further investigate how microglial cells are impacted by the introduction of *ApoeCh* on the PS19 background, we clustered all the microglia and DAMs from the four genotypes (13,940 total cells) into 9 new subclusters (Fig. 8k). Feature plots of microglial marker genes are shown in Fig. 8l, separating DAMs from homeostatic microglia. We identified 8 subclusters of homeostatic microglia (MGL1-8) and one subcluster of disease-associated microglia (DAM). Quantification of the proportion of cells in each of these subclusters reveals the distinct changes in each of these subclusters due to PS19 and the introduction of *ApoeCh*. PS19 mice show a reduction in the proportion of several homeostatic microglial clusters compared to WT (MGL-2,3,4,7,8) and a marked increase in DAMs. All microglial cluster proportions appear to be at WT levels in PS19;*ApoeCh* mice (Fig. 8m). Plotting these microglial subclusters in space identifies that while homeostatic microglia are expressed throughout the brain, cells in the DAM cluster are localized to the hippocampus in PS19 mice (Fig. 8n).

### Comparison of gene expression effects of ApoeCh in 5xFAD and PS19 mice

**Fig. 9:**
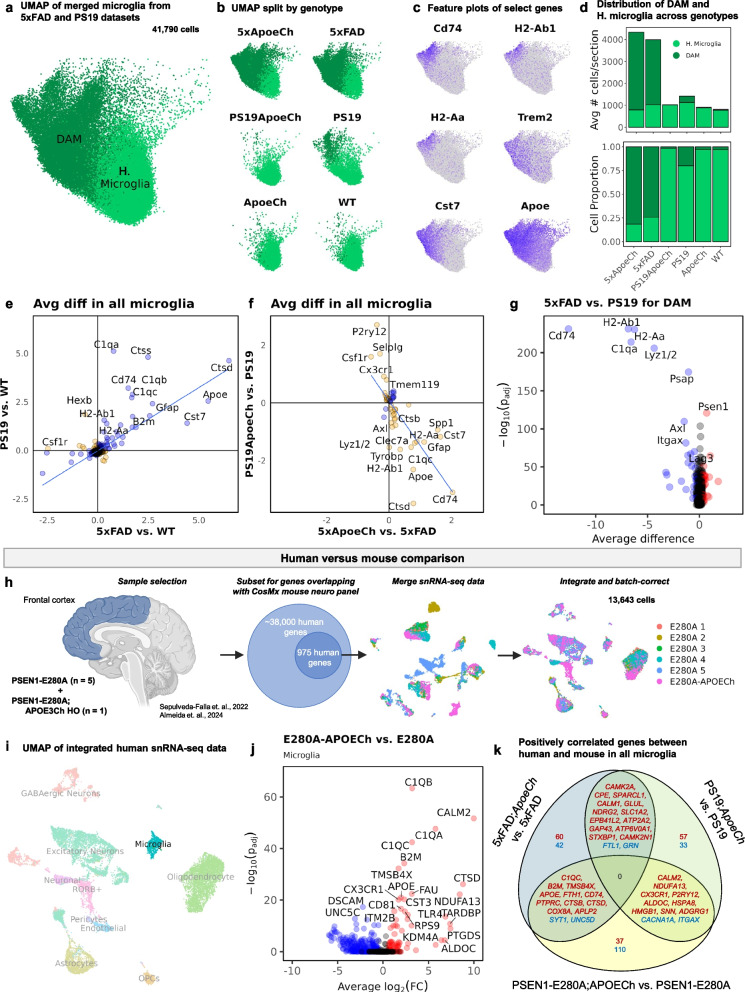
Microglia have differential responses to plaque and tau pathology. **a-g** Comprehensive analysis of all microglia (i.e., disease-associated and homeostatic) across both 5xFAD and PS19 cohorts.** a** UMAP of 41,790 microglia merged and integrated across both 5xFAD;*ApoeCh* and PS19;*ApoeCh* datasets (*n* = 3 for 5xFAD;*ApoeCh*, 5xFAD, PS19;*ApoeCh*, and PS19; *n* = 5 for *ApoeCh*; *n* = 6 for WT). Cells are labelled using their annotations from original datasets prior to merging. **b** UMAP of merged microglia split by genotype. DAMs in PS19 genotype are localized to one side of the DAM cluster in UMAP space. **c** Feature plots of DAM markers *Cd74*, *H2-Ab1*, *H2-Aa*, *Trem2*, *Cst7*, and *Apoe* in all microglia. **d** Distribution of DAMs and homeostatic microglia across genotypes. (d., Upper) Average microglial cell counts per section across each of the six genotypes. Cell counts for each genotype were divided by the total number of samples in that genotype. (d., Lower) Proportion of the number of cells in each genotype, grouped by DAM versus homeostatic microglia. **e** Scatterplot of the average difference in all microglia for all significant genes (i.e., p_adj_ < 0.05) between 5xFAD vs. WT and PS19 vs. WT comparisons. Directly correlated genes (blue) occur in the same direction for both comparisons, while inversely correlated genes (orange) occur in opposite directions for each comparison. Linear regression line showing the relationship between the two comparisons plotted in blue. **f** Scatterplot of the average difference in all microglia for all significant genes between 5xFAD;*ApoeCh* vs. 5xFAD and PS19;*ApoeCh* vs. PS19. **g** Volcano plot of DEGs between 5xFAD and PS19 DAMs. **h–k** Comparison of mouse single-cell spatial transcriptomics with human snRNA-seq data. **h** Schematic of data processing for snRNA-seq. Frontal cortex samples from the *PSEN1*-E280A heterozygous;*APOE3*Ch homozygous individual (*n* = 1) and *PSEN1*-E280A heterozygote controls (*n* = 5) were first reduced to 975 genes that overlapped with the CosMx mouse neuroscience panel, then processed through the standard Seurat pipeline with SCTransform. The two datasets were then merged and integrated using reciprocal PCA to account for batch effects between runs for a resulting dataset of 13,643 cells. **i** UMAP of integrated snRNA-seq data, with microglia highlighted. **j** DEGs between PSEN1-E280A;APOE3Ch vs. PSEN1-E280A in all microglia. Genes with p_adj_ < 0.05 are colored red (upregulated) or blue (downregulated). **k** Venn diagram of positively correlated genes between three comparisons: PSEN1-E280A;APOE3Ch vs. PSEN1-E280A (yellow oval), 5xFAD;*ApoeCh* vs. 5xFAD (blue oval), and PS19;*ApoeCh* vs. PS19 (green oval). Genes were considered positively correlated if the log-twofold-change of the average expression between the two groups was the same sign for both comparisons, and if the adjusted *p*-value was less than 0.05 for both comparisons. Upregulated genes are written in red text, while downregulated genes are written in blue text

We have shown that the *ApoeCh* variant has a differential impact on the microglial response dependent on disease pathology, enhancing the glial response to amyloid plaques in 5xFAD mice but tempering the response to tau pathology in PS19 mice. To gain insight into the molecules and underlying mechanisms of this differential impact of the *ApoeCh* variant on microglia, we first merged our spatial transcriptomic datasets from both the 5xFAD;*ApoeCh* and PS19;*ApoeCh* mice, yielding 41,790 Microglia/DAM cells (Fig. 9a). Next, we visualized microglial cell counts and proportions in each of the distinct animal lines (Fig. 9b-c). These data show the pronounced number and proportion of DAMs that make up the microglial population in 5xFAD (~ 75%), which appears expanded in 5xFAD;*ApoeCh* (~ 82%) mice, indicating that the *ApoeCh* variant encourages a DAM-like response or promotes the transition of more homeostatic microglia cells into DAMs in the presence of plaques, as we have described. In PS19 mice, we observe a much smaller number and proportion of DAMs (~ 20%), which are nearly absent in PS19*;ApoeCh* mice (Fig. 9b-c). These data confirm the opposing effects on numbers of DAMs in response to either plaques or tau pathology in the presence of *ApoeCh*.

To gain insight into the different genes and pathways involved in the differential *ApoeCh*-mediated microglial response to plaques and tauopathy, we compared how microglia respond to each of these pathologies. To capture this, all DEGs between 5xFAD vs. WT and PS19 vs. WT in all microglia and DAMs were plotted using the average difference of each gene among the two comparisons (Fig. 9e). Notably, many gene expression changes were shared and correlated between the two responses. In response to amyloid and tau, microglia exhibit down regulation in similar homeostatic genes, and an upregulation in many DAM genes (i.e., *Ctsd*, *Apoe*, *Cst7*, etc.). However, several genes showed differential regulation between the responses. For example, *C1qa-c, Ctss, Cd74, H2-Ab1, and H2-Aa* were more upregulated in the PS19 vs. WT response compared to the 5xFAD vs. WT response. Conversely, *Apoe* and *Cst7* were more upregulated in the 5xFAD vs. WT response. Homeostatic genes *Hexb* and *Csf1r* were downregulated in 5xFAD mice compared to WT, but unchanged or upregulated in PS19 vs. WT mice. Thus, while the overall response is similar (i.e., 5xFAD and PS19 microglia upregulate DAM genes in response to pathology), there are differences in related groups of genes in the magnitude of their response. For example, DAMs make up 74.2% of all microglia in 5xFAD brains compared to 20.0% in PS19 brains. Despite this difference, the overall response is remarkably similar in magnitude (i.e., average difference) in 5xFAD vs. WT mice compared to PS19 vs. WT mice, suggesting that microglia in PS19 have highly elevated DAM gene expression on a per-microglia basis compared to those in the 5xFAD brain. We plotted the average difference for all the DEGs from 5xFAD;*ApoeCh* vs. 5xFAD and PS19;*ApoeCh* vs. PS19 comparisons (Fig. 9f). As expected, gene expression changes were inversely correlated to each other, with most genes being upregulated in 5xFAD;*ApoeCh* vs. 5xFAD microglia/DAM, but downregulated in PS19;*ApoeCh* vs. PS19 microglia/DAM, reinforcing the opposing effects of *ApoeCh* on the microglial response to the two pathologies.

To directly compare the DAM response to either plaques or tauopathy, we plotted DEGs between DAMs in 5xFAD vs. PS19 mice (Fig. 9g). Consistent with fewer PS19 DAMs exhibiting the same magnitude in overall gene expression changes as the far greater number of 5xFAD DAMs, we observed that many genes were upregulated in PS19 DAMs compared to 5xFAD. Antigen presentation-associated genes were highly upregulated in PS19 compared to 5xFAD DAMs, including *Cd74, H2Ab1,* and *H2-Aa* (Fig. 9g). On the other hand, several genes were more upregulated in DAMs from 5xFAD brains compared to DAMs from PS19 brains, including *Cst7* and *Trem2* (Fig. 9g). Splitting the UMAP by genotype (Fig. 9b) reveals that PS19 mice only express a subset of DAMs that are found in 5xFAD mice. Visualization of *Cd74, H2-Ab1*, and *H2-Aa* on the UMAP show that DAMs in PS19 are specifically enriched in these genes, while *Trem2* and *Cst7* are enriched in DAMs found in 5xFAD mice (Fig. 9c). These results indicate that the responses of microglia to either plaques or tauopathy are fundamentally different from one another, providing a potential explanation as to how the *ApoeCh* variant can produce such contrasting effects.

### Comparison of mouse ApoeCh with human APOECh data

To compare our spatial transcriptomics findings in mouse with human studies, we integrated two publicly available human snRNA-seq datasets from frontal cortex of the *PSEN1*-E280A;*APOE*Ch homozygous individual (*n* = 1) and *PSEN1*-E280A controls (*n *= 5) and compared these with our mouse data (Fig. 9h; GSE206744, GSE222494) [35, 36]. As the greatest magnitude of difference was seen in microglia with *ApoeCh* for both 5xFAD and PS19 mice, we focused on the same cell type in human. DGE analysis of all microglia (i.e., homeostatic and disease-associated) in the human dataset revealed elevated microglial reactivity in the *PSEN1*-E280A heterozygote, *APOE*Ch homozygote compared to *PSEN1*-E280A heterozygote individuals, with upregulation of complement and inflammatory genes such as *C1QA*, *C1QB*, *C1QC*; CTSD, B2M; and *APOE* (Fig. 9j), in line with our findings of an increased inflammatory response to plaques with *ApoeCh*. Many of these genes correlated positively with those upregulated with *ApoeCh* in 5xFAD mice, including *C1QC*, *CD74*, *CTSD*, and *APOE* (Fig. 9k, Supp. Figure 21b). Several genes also correlated positively with those upregulated with *ApoeCh* in PS19 mice, including *CX3CR1*, *ALDOC*, *CALM2*, but as a whole there was no statistical correlation between the human data and PS19 data (Fig. 9k, Supp. Figure 21c). Given that the PSEN1-E280A;APOECh individual developed relatively limited tau pathology but had extensive plaque load, we view these findings to be consistent with our findings in mouse.

### Unbiased proteomics show differential effects of ApoeCh in 5xFAD vs. PS19 mice

**Fig. 10:**
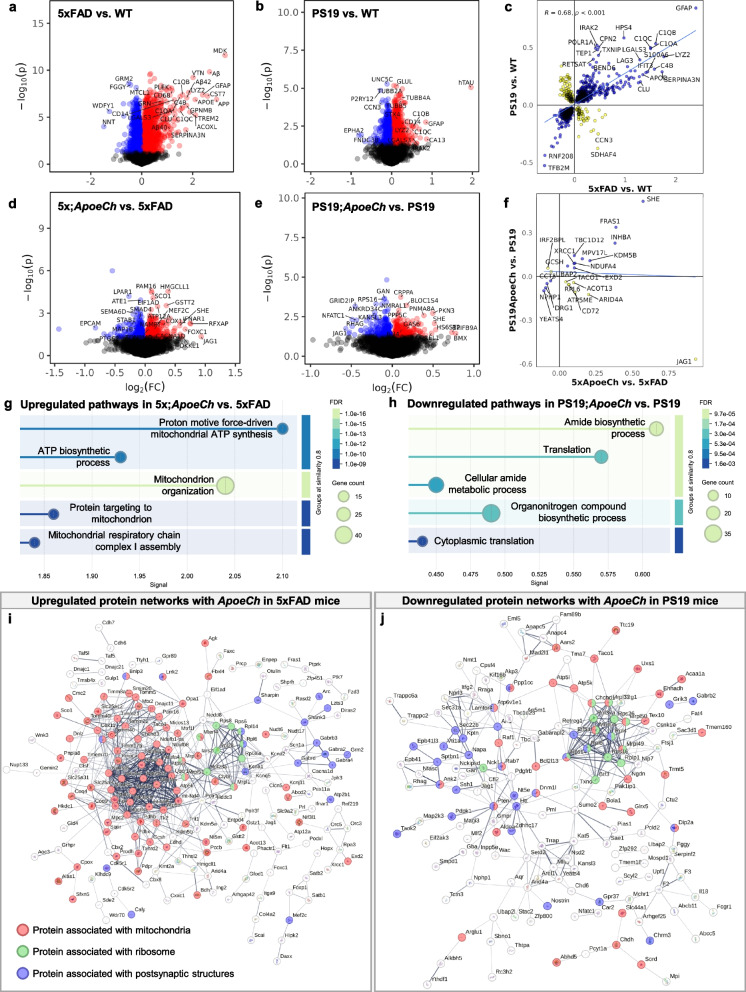
Unbiased proteomics reveal differential effects of *ApoeCh* in 5xFAD versus PS19 mice.** a** DAPs between 5xFAD vs. WT mice. Log-twofold-change on the x-axis, -log-10 *p*-value on the y-axis. Proteins with *p*-value < 0.05 are colored in red (upregulated) or blue (downregulated. **b** DAPs between PS19 vs. WT mice. **c** Scatterplot of DAPs from comparisons in **a** and **b**. Log-twofold-change values of all proteins with *p*-value < 0.05 are plotted, with 5xFAD vs. WT comparison on the x-axis and PS19 vs. WT on the y-axis. Correlation coefficient R = 0.68, *p* < 0.001. **d** DAPs between 5xFAD;*ApoeCh* vs. 5xFAD mice. **e** DAPs between PS19;*ApoeCh* vs. PS19 mice. **f** Scatterplot of DAPs from comparisons in **d** and **e**. 5xFAD;*ApoeCh* vs. 5xFAD on x-axis, PS19;*ApoeCh* vs. PS19 on y-axis. **g** Pathway analysis of all significantly upregulated proteins in the 5xFAD;*ApoeCh* vs. 5xFAD comparison. **h** Pathway analysis of all significantly downregulated proteins in the PS19;*ApoeCh* vs. PS19 comparison. **i** Protein–protein interaction (PPI) network of all significantly upregulated proteins in the 5xFAD;*ApoeCh* vs. 5xFAD comparison. Nodes are colored according to function, including mitochondrial associated (red), ribosome associated (green), and postsynaptic structure proteins (blue). The thickness of the line connecting two nodes indicates the degree of confidence in the prediction of the PPI. **j** PPI network of all significantly downregulated proteins in the PS19;*ApoeCh* vs. PS19 comparison

To obtain an unbiased view of how the presence of *ApoeCh* influences the proteome, we performed bulk proteomics on cortical samples from the 12-month cohort of 5xFAD mice and 9-month cohort of PS19 mice. Differential abundant proteins (DAPs) were calculated for 5xFAD vs. WT and PS19 vs. WT cortices (Fig. 10 a,b; Supp. Figure 22). Upregulated proteins in 5xFAD brains were highly concordant with known increases in gene expression, with many inflammatory proteins overexpressed, including GFAP, CST7, TREM2, complement proteins C1QA-C, APOE, LGALS3 and GPNMB. MDK was the most overexpressed protein in 5xFAD mice and has recently been identified as also highly overexpressed in human AD brains [74]. PS19 mice also show robust increases in inflammatory proteins, such as GFAP, LGALS3, C1QB, and C1Q. To directly compare the responses in 5xFAD mice vs. PS19 mice we selected all proteins that were significantly altered (*p* < 0.05) in both comparisons and plotted them by log-twofold-change (Fig. 10c). A strong positive correlation was seen between the 5xFAD and PS19 mice in the upregulation of inflammatory proteins, including complement proteins C1QA, C1QB, C1QC, C4B; antigen presentation proteins H2-K1, H2-D1, B2M, TAP2; apolipoproteins APOE, APOD, CLU; microglial proteins CSF1R, HEXB, CD68, ITGB2; cathepsins CTSS, CTSZ and reactive astrocyte markers GFAP and SERPINA3N (top right quadrant, Fig. 10c). There was a strong positive correlation of downregulated proteins, including neuronal and synaptic proteins GRIK1, NGB, KCNS2; mitochondrial proteins TFB2M, TMA16, LYRM2; and protein folding/regulation proteins TNF208, TOR2A, DNAJC4 (bottom left quadrant). Conversely, there were several proteins differentially upregulated in the 5xFAD comparison that were downregulated in the PS19 comparison, such as neuronal and synaptic proteins NPTXR, CPNE6, KIRREL3, TMEFF2, SPON1; and P2Y receptor proteins P2RY12 and P2RY13 (bottom right quadrant). There were also proteins differentially upregulated in the PS19 comparison that were downregulated in 5xFAD, notably beta-tubulin isotypes TUBB1, TUBB2A, TUBB3, TUBB4A, TUBB5, TUBB6, TUBAL3; other microtubule proteins MAPRE3, DPYSL2, DPYSL3; and myelin proteins MOG, MAG, CNP (top left quadrant).

Next, we examined the effects of *ApoeCh* on the proteome in either 5xFAD (Fig. 10d) or PS19 (Fig. 10e) mice, as well as a comparison of the changes common to both (Fig. 10f). Indeed, there are few overlapping DAPs with *ApoeCh* in the two mouse models, with concordant upregulation in SHE, the extracellular matrix protein FRAS1, the TGF-beta family member INHBA, and the lysine demethylase KDM5B among others, and concordant downregulation in the NuA4 histone acetyltransferase complex component YEATS4, and microtubule associated proteins NPHP1 and DRG1. Notably, the notch signaling ligand JAG1 was highly discordant, being highly upregulated by *ApoeCh* in 5xFAD mice, but highly downregulated by *ApoeCh* in PS19 mice. JAG1 has been shown to regulate microglial function and inflammatory responses [75]. Overall, few inflammatory proteins were significantly impacted by *ApoeCh* in either model and the vast number of changes detected were in metabolism associated proteins, and these showed highly differential effects in 5xFAD mice vs. PS19 mice. For example, pathway analyses show a large upregulation of a network of mitochondrial associated proteins with *ApoeCh* in 5xFAD mice (Fig. 10g, i), while mitochondrial and ribosomal/translation associated proteins are downregulated in PS19 mice (Fig. 10h, j). Synapse associated proteins were also overrepresented in both comparisons. Protein pathways and interacting networks are shown for downregulated proteins in 5xFAD mice (Supp. Figure 22a, c), and upregulated proteins in PS19 mice (Supp. Figure 22b, d), with *ApoeCh*, but no strong networks emerge. Due to the nature of bulk tissue proteomics, we cannot determine in which cell types these changes in metabolism are occurring, but it is known that disease associated microglia upregulate mitochondrial complex 1 activity to sustain the response to pathology [75], in line with our pathway analyses of *ApoeCh* in 5xFAD mice. Thus, bulk proteomics reveals differential effects of *ApoeCh* in 5xFAD and PS19 cortices, again supporting the notion that APOECh has highly context dependent effects on the brain’s response to pathologies.

## Discussion

The recent identification of an ADAD *PSEN1*-E280A carrier who was homozygous for the rare *APOE* Christchurch variant (R136S, *APOECh*) and who was resistant to the onset of ADAD dementia until her 70’s was a remarkable discovery [15]. Due to the inherent nature of a single human case report, it was formally unknown whether the APOECh variant was responsible for the resilience to AD observed in this person. For this reason, we used genetically modified mouse models to investigate whether introduction of the Christchurch variant into mouse APOE could modify development of amyloid and tau-related pathologies. Using spatial transcriptomics and proteomics, we show that the mouse *ApoeCh* variant enhances plaque-associated microgliosis in 5xFAD mice but suppresses the glial response to tauopathy in PS19 mice. Additionally, we also report differential changes in mitochondria and translation associated protein networks between 5xFAD and PS19 with the presence of the mAPOECh variant, via unbiased proteomics. These results support that the APOECh variant could contribute to the resilience to AD observed in the PSEN1-E280A carrier in the case report and offer insight into the potential mechanisms for this effect. While this report was being prepared, independent studies reported similar findings [41, 76, 77]. We discuss our results and conclusions in the context of those studies.

Mouse models are powerful systems to explore mechanisms underlying the development of pathology and the brain’s response to it. While no single mouse model effectively recapitulates human AD, the field routinely utilizes mice that have been engineered to develop either extensive amyloid pathologies (e.g., the 5xFAD model) or tauopathy (e.g., the PS19 model). Both models rely on transgene overexpression and familial mutations (such as ADAD mutations in 5xFAD mice and frontotemporal dementia mutations in PS19 mice) and are not well suited for studying the causative events that lead to the development of pathology in sporadic AD [44–47]. However, they are useful for examining the brain's response to Aβ- and tau-associated pathology, in this instance in the context of the APOE Christchurch variant.

We decided to analyze the effect of introducing the Christchurch variant into the mouse APOE protein (mAPOE) rather than the human protein (hAPOE), as there is overall limited sequence homology between the two species (~ 70%). However, mAPOE and hAPOE have a conserved LDLR and HSPG binding motif between AA136 to 150 (human numbering) [11, 12, 39], which includes the R136S Christchurch variant. Thus, modifying the mouse *Apoe* locus within this conserved binding motif allows us to explore how it regulates the brain’s response to pathology while excluding the potential confound associated with interaction of hAPOE with murine receptors.

Three other APOE Christchurch mouse models have recently been described, all with Christchurch introduced into human sequences (including both hAPOE3 and hAPOE4) with some contrasting results [41, 76, 77]. Introduction of hAPOE3Ch into APP/PS1 mice (with and without injection of human AD-tau) resulted in decreased plaque load and AD-tau seeding and spreading, alongside increased microglial reactivity around plaques [41]. In PS19 mice, a homozygous R136S mutation rescued APOE4-dependent tau pathology and hippocampal atrophy, with a reduction in microgliosis [76]. In addition, the R136 mutation in PS19 mice (on a hAPOE3 background) resulted in protection against tau pathology and tau-induced synaptic deficits while suppressing interferon microglial activation [77]. Thus, the impact of the Christchurch variant appears to be dependent on the stimulus – i.e., plaques or tau-laden neurons. In mice homozygous for the Christchurch variant, these studies indicate that microglial activation states are suppressed in PS19 mice (tau-associated pathology), promoting a homeostatic or “disease protective” response, whereas they are enhanced in APP/PS1 mice (amyloid-associated pathology), promoting a “disease-associated” state. To dissect the differential microglial responses to pathology, we generated *ApoeCh* homozygous mice independently with both 5xFAD and PS19 lines, which enabled us to directly compare the impact of *ApoeCh* on the response to amyloid and tau pathology individually. The results we obtained complements, validates, and helps reconcile these previous findings.

Our study reveals that introduction of *ApoeCh* into the 5xFAD mouse model reduces plaque load and reactive astrocytic and microglial presence, as well as ameliorates plaque-induced neuritic and axonal damage, consistent with the study involving humanized *APOE3Ch* knock-in mice crossed to APP/PS1 mice [41]. Despite a reduction in glial numbers in the pathology rich subiculum, spatial proteomics and transcriptomics reveal a significant brain wide upregulation of disease-associated microglia and astrocytes in 5xFAD;*ApoeCh* compared to 5xFAD mice. These observations are likely explained by regional differences (i.e., whole brain quantification vs. subiculum), and that *ApoeCh* may increase the likelihood of a microglia transitioning to a DAM state—spatial proteomics shows only minor changes in IBA1 expression, but strong upregulation of DAM marker proteins such as APOE and CD11C. In agreement with Chen et al. [41] who showed increased myeloid cell phagocytosis in humanized APOE3Ch, we show that *ApoeCh* produces upregulation of several genes associated with autophagy/endocytosis and antigen presentation (*Mid1, Cd74, H2-Aa, H2-Ab1, Spp1, Gpnmb*, *Cst7*) in 5xFAD mice. A recent report provides evidence that autophagy plays an important role in regulating microglial activation (i.e., DAM phenotype induction) and that deficiency in autophagy leads to senescence, reduces DAMs, impairs Aβ clustering around plaques, and aggravates AD pathology [78]. Given the reduction in amyloid-associated damage, these data implicate a neuroprotective role of enhanced DAM/microglial reactivity to amyloid pathology with the introduction of *ApoeCh* in 5xFAD mice.

Crossing *ApoeCh* mice to PS19 mice reveals the divergent impact of *ApoeCh* on pathology and microglia in a mouse model of tauopathy. We show that introduction of *ApoeCh* reduces microglia and reactive astrocytes, similar to 5xFAD mice, but is accompanied by a reduction in DAM and DAA as well as associated proteins and genes, thereby promoting a more homeostatic state, and counter to changes in models of amyloidosis. Contrary to studies in humanized mice [76, 77], our study does not show that *ApoeCh* rescues tau pathology or functional deficits. Chen et al. [41] report no difference in tau with introduction of humanized *APOE3Ch* in the absence of amyloidosis. In line with humanized *APOECh* observations, we demonstrate a diminished DAM and astrocyte response to tau [76, 77]. Moreover, we provide evidence that *ApoeCh* results in the rescue of tau-induced synaptic and myelin loss in PS19, associated with the rescue of transcription changes in oligodendrocytes and neurons [76, 77]. Together, our results provide insight into the multiple effects of the Christchurch variant, which enhances DAM reactivity against plaques while promoting a more homeostatic or “disease protective” response to tau.

Given the multifaceted roles of APOE and its receptors in AD pathologies, the protective effects conferred by the Christchurch variant likely involve multiple mechanisms. Previous studies provide evidence that the Christchurch variant reduces AD pathology and associated CNS damage at least in part through alterations in receptor binding to LRP1, LDLR and/or HSPGs [15, 41, 76]. Consistent with data indicating that *APOE3Ch* decreases the binding affinity to these molecules [41, 79], our data shows that introduction of *ApoeCh* causes elevated plasma cholesterol. However, our data also indicates that the Christchurch variant may influence Aβ pathology by directly affecting microglial programming. 5xFAD;*ApoeCh* mice show changes in DAM-associated gene expression that are heavily implicated in inflammation, particularly autophagy and phagocytosis. Krasemann et al. showed that the DAM (DAM/MGnD) signature is dependent on the TREM2-APOE pathway [57]. Thus, triggering receptor expressed on myeloid cells 2 (TREM2), a microglia cell surface receptor involved in phagocytosis and regulation of inflammation, could also in part contribute to protective *ApoeCh*-associated amyloid changes. APOE-TREM2 interactions have been found to facilitate phagocytosis of apoptotic neurons and increased uptake of Aβ [80–83].

HSPGs are responsible for neuronal uptake of tau and the R136S mutation causes impaired binding affinity of APOE to HSPGs, leading to reduced tau pathology [15, 41, 76, 84, 85]. We show that introduction of *ApoeCh* in PS19 mice leads to a downregulation in DAM and disease associated astrocytes (DAA), coupled with the upregulation of oligodendrocyte-related genes and rescue of myelin loss. Shi et al. [86] report that overexpression of LDLR in PS19 mice suppresses microglial activation, enlarges pools of oligodendrocyte progenitor cells, and enhances preservation of myelin integrity. Thus, introduction of *ApoeCh* in tauopathy murine models might also promote or alter LDLR signaling. These data also suggest that *ApoeCh* may also modulate the interactions or crosstalk between microglia, astrocytes, and oligodendrocytes. Activated microglia, via C1q release, have been implicated as potential mediators of neurotoxic effects, resulting in reactive astrocyte-mediated neuronal and oligodendrocyte death [87]. Notably, we also observed reduction in *C1q* expression in PS19;*ApoeCh* mice. Our spatial transcriptomics data also reveals that PS19 mice exhibit an upregulation in several MHC II-antigen presentation-related genes, which is dampened by *ApoeCh*. A prior investigation highlighted the higher inflammatory reactivity (i.e., microglial MHC II + proteins and T cells) associated with tau-induced neurodegeneration, but not those with amyloid deposition [88]. Moreover, *Apoe* deletion rescued brain atrophy and altered microglia and T cells, tying together the role of APOE and the innate and adaptive immune responses to tau vs. plaques via microglia [88]. In line with this, we report that *ApoeCh* produces an overall decrease in inflammatory response to tau, suggesting the impaired binding of APOE to its receptors plays an important role in neuroinflammation.

The role of microglia in the development and progression of AD is a hotly debated topic [89–92]. Genetic association data implicate microglial expressed genes, and hence microglia, with altered risk for developing the disease, but the underlying mechanisms are not yet fully understood [93–95]. Further, the microglial responses to plaques, tangles, and dying neurons are prominent in the human AD brain as well as in animal models, while aberrant microglial activity is associated with synaptic and neuronal loss [96–98]. The current consensus is that the microglial response to plaques confers protection. Indeed, approaches are underway to further induce this microglial response through TREM2 agonists and other approaches [99, 100]. Conversely, the microglial response to tauopathy appears to drive neurodegeneration. For instance, microglial elimination from PS19 mice or suppression of inflammation prevents tau-induced neurodegeneration [101–103], while APOE2 or *Apoe* KO protects against, and APOE4 exacerbates, tauopathy-induced neurodegeneration [29]. Understanding how changes in microglial biology, triggered by different stimuli or pathological states, can induce either protection or damage should yield insights for the timing of therapeutic interventions. Introduction of the *ApoeCh* variant represents a therapeutic strategy that presents a nuanced approach to modulating distinct microglial responses in the presence of amyloid vs. tau pathology. Understanding the interactions of APOE and how the R136S variant alters those interactions may provide small molecule opportunities for targeted glial therapeutics. Outside of small molecules that target the R136 region of APOE, introduction of *APOE3Ch* into humans may offer another avenue for lifelong protection, whether through genome editing or other means. In this study, we employed single-cell spatial transcriptomics imaging to examine the transcriptional changes induced by the introduction of the *ApoeCh* variant. This technique offers a powerful advantage by providing both transcriptomic and spatial information, which is lacking in traditional single-cell/single-nucleus approaches. However, it is limited by the pre-selected mouse neuroscience 1000-plex probe set, which constrains the level of exploratory or unbiased investigation compared to the aforementioned techniques. Although the current probe set broadly covers major biological pathways, it restricts in-depth investigations due to the limited selection of RNA targets.

We note that the pathological effects observed in the transgenic mouse models should be carefully interpreted, taking into account the transgene and APOE isoform-specific effects on plaque and tau pathology. Introducing the Christchurch variant into the mouse *Apoe* locus helps mitigate potential issues related to humanized proteins interacting with murine receptors. However, mAPOE has been shown to have different effects on Aβ plaque metabolism and formation compared to human APOE [104, 105]. It will be important to compare and contrast the results from our murine APOECh model to those produced on humanized APOE backgrounds to understand the caveats and relevance of each approach. Furthermore, while crossing the *ApoeCh* mice with amyloidosis and tauopathy models in parallel provides important insights into how the variant independently affects Aβ plaques and tau, it is important to investigate the effect of the *ApoeCh* variant in a mouse model that develops both Aβ and tau pathologies in an age appropriate manner, ideally not produced via classical transgenes. This approach should offer a more comprehensive understanding of the role and mechanism of APOECh in Alzheimer's disease.

## Conclusions

The R136S mutation occurs within a conserved HSPG/LDLR binding region of APOE, and its introduction in the murine genome/proteome allows us to study its function in its natural interactome and complement findings from humanized APOECh mouse models. By independently crossing with a model of amyloidosis and a model of tauopathy, we show that the murine APOECh variant effects the microglial response to both pathologies, but in opposite directions. Murine APOECh promotes the response of microglia to plaques, but suppresses the response to tauopathy, seemingly providing protection in both situations. Thus, murine APOECh appears to provide a nuanced and context-dependent modulation of the microglial response, highlighting the importance of microglial biology and APOE to the development and progression of AD.

## Supplementary Information

Supplementary Material 1. Supp. Table 1 - Location of potential off-target sites for crRNA TMF1648 on mouse chromosome 7. The desired target site within *Apoe* locus is listed, plus the sequence of each of the 12 potential off-target sites on mouse chromosome 7 (GRCm38/mm10 nucleotide numbering). Green text denotes the 11-nucleotide seed region proximal to the PAM site. Mismatches to the guide are indicated by lowercase bold letters and the number of mismatches (including the number within the seed region) is shown. No difference was found in sequence between the C57BL/6J WT and *Apoe*^*em1Aduci*^ alleles at the six potential off-target sites analyzed (Supplementary Fig. 1b).

Supplementary Material 2. Supp. Table 2: Primers for PCR amplification and sequencing for off-target analysis. Forward (For) and reverse (Rev) primer sequences are listed for each potential off-target site. The off-target code corresponds to the panels in Supplementary Fig. 1.

Supplementary Material 3. Supp. Figure 1: Sequence of the *Apoe*^*em1Aduci*^ (*ApoeCh*) allele and off-target site analysis for crRNA TMF1648 on mouse chromosome 7. (a) Amino acid (aa) sequence alignment between human and mouse APOE and DNA sequence of the wildtype and *ApoeCh* alleles in the region of the APOE R136S (R128S in mouse mature APOE). Red-colored nucleotides denote the missense codon while the green nucleotides denote synonymous base changes introduced to prevent recutting of the targeted site. (b) Chromatograms of DNA sequence in wildtype and *ApoeCh* heterozygous mice, with colored asterisks corresponding to the colored nucleotides in the above sequence. (c-i) Chromatograms of N3F2 wildtype and *ApoeCh* homozygous offspring at six potential off-target sites on mouse chromosome 7. No difference was found in sequence between the B6J WT and *ApoeCh*homozygotes at each of the six potential off-target sites analyzed. The black underline denotes the crRNA target sequence while the blue underline denotes the NGG PAM site. Supp. Figure 2: Behavioral analysis of 4- and 12-mo-old WT, *ApoeCh*, 5xFAD, 5xFAD;*ApoeCh* mice. a,b Plasma triglyceride (a) and VLDL (b) level in 4 mo WT and *ApoeCh* HO mice. c,d Weight of 4-mo-old (c) and 12-mo-old (d) mice taken at euthanizing day. e, f Total time mice spent in the center of open field behavioral assay of 4-mo-old (e) and 12-mo-old (f) in the 5 min recording time. g,h Mean velocity mice traveled in the center of the open field in 5 min of 4-mo (g) and 12-mo-old mice (h). i, j Total time 4-mo (i) and 12-mo-old (j) mice spent in open arms of the elevated plus maze behavior test. *n *= 4-6 mice/sex/genotype. Data are represented as mean ± SEM. Student’s t-test, unpaired. Two-way ANOVA followed by Tukey’s post hoc tests to examine biologically relevant interactions. Statistical significance is denoted by *p<0.05,**p<0.01, ***p<0.001, ****p<0.0001. # denotes trending significance. Supp. Figure 3: Sex-specific differences in pathology in the 5xFAD mice. In each panel, sex of individual animals is denoted by pink (female) or blue (male) circles. a-c Sex-separated plasma cholesterol (a), triglycerides (b), and VLDL (c) in 4-mo-old WT and *ApoeCh *mice. d-e Weights of 4-mo (d) and 12-mo-old WT, *ApoeCh*, 5xFAD, and 5xFAD;*ApoeCh *mice separated by sex. f-i Quantification of Amyloglo+ plaque numbers in 4-mo (f) and 12-mo (h); and percent plaque coverage in 4-mo (g) and 12-mo (i) in whole brain hemisphere images of 5xFAD and 5xFAD;*ApoeCh* mice separated by sex. j-k Sex-separated LAMP1+ dystrophic neurites volume in 4-mo (j) and 12-mo (k) 5xFAD and 5xFAD;*ApoeCh *subiculum. l-m Plasma NfL levels of 4-mo (l) and 12-mo (m) WT, *ApoeCh*, 5xFAD, and 5xFAD;*ApoeCh *mice separated by sex. *n *= 4-6 mice/sex/genotype. Data are represented as mean ± SEM. Two-way ANOVA followed by Tukey’s post hoc tests to examine biologically relevant interactions. In comparisons with 2 genotypes (i.e. 5xFAD, and 5xFAD;*ApoeCh)*, multiple comparisons between all groups were performed. Comparisons with 4 genotypes (i.e. WT, *ApoeCh*, 5xFAD, and 5xFAD;*ApoeCh), *only comparisons between genotypes were analyzed and reported. Statistical significance is denoted by *p<0.05, **p<0.01, ***p<0.001, ****p<0.0001. # denotes trending significance. Supp. Figure 4: Quantification of insoluble and soluble Aβ in micro-dissected hippocampi and cortices of 4-mo-old 5xFAD, 5xFAD;*ApoeCh* mice. In each panel, sex of individual animals is denoted by pink (female) or blue (male) circles. a, d Cortical and hippocampal soluble Aβ40 (a,b) and Aβ42 (c,d) level measured via MSD of 4-mo-old mice. e-h Cortical and hippocampal soluble Aβ42 (e,f) and Aβ42 (g,h) level measured via MSD of 4-mo-old mice. i-p Sex-separated data of 4-mo cortical and hippocampal Aβ levels from a-h. q-x Sex-separated data of 12-mo cortical and hippocampal Aβ levels from Figure 2f-m. *n *= 4-6 mice/sex/genotype. Data are represented as mean ± SEM. Student’s t-test, unpaired. Two-way ANOVA followed by Tukey’s post hoc tests to examine sex and biologically relevant interactions. Statistical significance is denoted by *p<0.05, **p<0.01, ***p<0.001, ****p<0.0001. Supp. Figure 5: *ApoeCh *mutation induces no change in synapses of the 5xFAD mice. a Representative super-resolution images of Bassoon and Homer1 synaptic markers for WT, *ApoeCh*, 5xFAD, and 5xFAD;*ApoeCh* mice at 12 mo of age. Scale bar = 10µm. Insert scale bar = 1µm. b-d Quantification of Bassoon+ spots per µm^3^, Homer1+ spots per µm^3^ and colocalized Bassoon+/Homer1+ synaptic spots per µm^3^. e-g Quantification of Bassoon+ spots per µm^3^, Homer1+ spots per µm^3^ and colocalized Bassoon+/Homer1+ synaptic spots per µm^3^ separted by sex. Three images per mouse and *n* = 5-6 mice/sex/genotype. In each panel, sex of individual animals is denoted by pink (female) or blue (male) circles. Data are represented as mean ± SEM. Two-Way ANOVA followed by Tukey’s post hoc test to examine biologically relevant interactions. Comparisons with 4 genotypes (i.e. WT, *ApoeCh*, 5xFAD, and 5xFAD;*ApoeCh), *only comparisons between genotypes were analyzed and reported. Statistical significance is denoted *p<0.05, **p<0.001. Supp. Figure 6: Quantification of astrocyte and microglia in 4-mo-old WT, *ApoeCh*, 5xFAD, 5xFAD;*ApoeCh* mice. a, c Representative 20x confocal images of the subiculum stained for dense-core plaque with AmyloGlo (green) and immunolabeled for astrocyte with GFAP (red, a) and IBA1 for microglia (red, c). Scale bar=100µm. In each panel, sex of individual animals is denoted by pink (female) or blue (male) circles. b, d Total GFAP+ (b) and IBA1+ volume (d) in the subiculum per FOV of 4-mo-old WT, *ApoeCh*, 5xFAD, 5xFAD;*ApoeCh* mice. e, f Total number of IBA1+ cells per FOV of 4 mo (e) and 12 mo (f) WT, *ApoeCh*, 5xFAD, 5xFAD;*ApoeCh* mice in the subiculum. g-h Sex-separated 4-mo (g) and 12-mo (h) quantification of GFAP+ astrocyte volume in the subiculum. i-l Sex-separated 4-mo IBA1+ total volume (i) and cell number per FOV (j); and 12-mo IBA1+ total volume (k) and cell number per FOV (l) in the subiculum of WT, *ApoeCh*, 5xFAD, 5xFAD;*ApoeCh* mice. *n* = 4-6 mice/sex/genotype. Data are represented as mean ± SEM. Two-way ANOVA followed by Tukey’s post hoc tests to examine biologically relevant interactions. For sex-stratified comparisons*, *only comparisons between genotypes were analyzed and reported. Statistical significance is denoted by *p<0.05, **p<0.01, ***p<0.001, ****p<0.0001. # denotes trending significance. Supp. Figure 7: Cell segmentation and annotation in 5xFAD;*ApoeCh* spatial proteomics data. a Representative images of cell segmentation (teal outline) in cortex, dentate gyrus (i.e., high cell density region), and white matter tracts for 5xFAD;*ApoeCh* spatial proteomics dataset. DAPI nuclear stain is shown in light grey. White dotted line demarcating anatomical landmarks for dentate gyrus and white matter tracts. Scale bar = 50 µm. b 12 CELESTA cell types in XY space for all 12 hemibrains (*n *= 3/genotype). Supp. Figure 8: Cell segmentation and annotation in 5xFAD;*ApoeCh* spatial transcriptomics data. a Representative images of cell segmentation (teal outline) in white matter tracts, CA1, and cortex for 5xFAD;*ApoeCh* spatial transcriptomics dataset. DAPI nuclear stain shown in light grey, and histone marker shown in green. White dotted lines demarcating anatomical landmarks for white matter tracts and CA1 of hippocampus. Scale bar = 50 µm. b 33 cell types in XY space for all 12 sections (*n *= 3/genotype). Supp. Figure 9: Distribution of cell types across genotypes in 5xFAD;*ApoeCh* dataset. a-b Data quality is comparable across samples. a Number of unique transcripts per cell, grouped by sample. b Number of unique genes (features) per cell, grouped by sample. c UMAP of cells from 5xFAD;*ApoeCh* spatial transcriptomics dataset split by genotype (5xFAD;*ApoeCh*, 5xFAD, *ApoeCh*, WT). DAM (dark green) and DAA (dark orange) clusters are primarily present in 5xFAD;*ApoeCh* and 5xFAD genotypes, but not *ApoeCh* and WT genotypes. d Heatmap of the top five marker genes per major cell type. DGE analysis was performed between each major cell type compared to all other major cell types to identify the top 5 genes expressed for each major cell type. e Stacked bar plot of cell counts of all 33 clusters, split by genotype. Clusters are grouped by their major cell type (i.e., ODC 1-5 and OPC are grouped together on the bar plot), and then within each major cell type plotted by size in descending order. Supp. Figure 10: Differential gene expression analysis between 5xFAD vs. WT and 5xFAD;*ApoeCh* vs. 5xFAD across major cell types and all cell types. a Volcano plots showing DEGs between 5xFAD and WT across each cluster. Because DAMs and DAAs are present in 5xFAD but not in WT mice, DAMs and microglia were grouped together into “All microglia”, and DAAs and astrocytes were grouped together into “All astrocytes” for the 5xFAD vs. WT comparison. Each gene represents a point on the volcano plot. Red and blue colored points represent significantly up- and downregulated genes, respectively (i.e., p_adj_ < 0.05 and absolute average difference > 0.3). Black points represent unsignificant genes (i.e., p_adj_> 0.05 or absolute average difference < 0.3). Only the top 20 genes are labelled for legibility. b Volcano plots showing DEGs between 5xFAD;*ApoeCh* and 5xFAD across each cluster. Dotted red outlines highlight clusters CA1 and L2/L3, which are shown to have the greatest (most negative) DD score in the 5xFAD;*ApoeCh* vs. 5xFAD comparison (Fig. 4i). c Volcano plots showing DEGs between 5xFAD and WT across each major cell type. DAMs and microglia are grouped into “All microglia”, and DAAs and astrocytes into “All astrocytes”. Supp. Figure 11: Differential gene expression changes in L2/L3 of cortex and CA1 of hippocampus. a Examples of high spatial specificity for dentate gyrus, CA1, and L2/L3 clusters in a 5xFAD;*ApoeCh* section. b Dot plot showing pseudo-bulk expression of the top DEGs in the 5xFAD;*ApoeCh* vs. 5xFAD comparison across all four genotypes in L2/L3 and CA1 clusters. Supp. Figure 12: Microglial subclustering in 5xFAD;*ApoeCh* dataset. a Feature plots for common microglial and DAM marker genes. b Example of cell segmentation and cell annotation surrounding an amyloid beta plaque in a 5xFAD;*ApoeCh* brain. (top) White arrows pointing to two cells annotated as amyloid beta plaques. Many cells in this plaque cluster were anucleate (i.e., did not contain histone marker). DAPI nuclear stain (light grey), histone marker (green). (bottom) Cell type annotation for all microglia. Cells annotated as Abeta plaques (red) are surrounded by 5 DAM-2 cells (turquoise), 2 DAM-1 cells (blue), and a Microglia-1 (magenta). c Heatmap of the top 10 marker genes for each microglial subcluster. DGE analysis was performed for each microglial subcluster compared to all other microglial subclusters to obtain the top 10 genes expressed in each microglial subcluster. Supp. Figure 13: Behavioral analysis of 5- and 9-mo-old WT, *ApoeCh*, PS19, PS19;*ApoeCh* mice. In each panel, sex of individual animals is denoted by pink (female) or blue (male) circles. a, b Weight of 5-mo-old (a) and 9-mo-old (b) mice taken at euthanizing day. c, d Violin plot of hindlimb clasping score of 4-mo-old (c) and 12-mo-old (d) mice. e, g Total time mice spent in the center of open field behavioral assay of 5-mo-old (e) and 9-mo-old (g) in the 5 min recording time. f, h Mean velocity mice traveled in the center of the open field in 5 mins of 5-mo (f) and 9-mo-old mice (h). i, j Total time 5-mo (i) and 12-mo-old (j) mice spent in open arms of the elevated plus maze behavior test. k, l Measurement of plasma NfL level in WT, *ApoeCh*, PS19, and PS19;*ApoeCh* mice at 5- (k) and 9-mo (l). *n *= 4-10 mice/sex/genotype. Data are represented as mean ± SEM. Two-way ANOVA followed by Tukey’s post hoc tests to examine biologically relevant interactions. Statistical significance is denoted by *p<0.05, **p<0.01, ***p<0.001,****p<0.0001. # denotes trending significance. Supp. Figure 14: Immunohistochemical and biochemical analyses of tau in 5 and 9-mo-old WT, *ApoeCh*, PS19, PS19;*ApoeCh* mice. In each panel, sex of individual animals is denoted by pink (female) or blue (male) circles. a Representative 20x confocal images of the dentate gyrus (top) and piriform cortex (bottom) immunolabeled with AT8 for phosphorylated tau (green). Scale bar = 100µm. b, c Percent coverage of AT8+ area per FOV in the dentate gyrus (b) and piriform cortex (c). d Representative whole hemisphere stitched images of MC1 immunolabeled 9-mo WT, PS19, and PS19;*ApoeCh *mice with zoomed-in inset of MC1+ inclusion in the cortex. Scale bar = 500µm e Quantification of MC1+ inclusions number in the hemisphere stitched image of 9-mo PS19 and PS19;ApoeCh mice. f-k Measurement of total tau in RAB, RIPA, and formic acid fraction of the micro-dissected hippocampi (f-h) and cortices (i-k) of 9-mo-old WT, *ApoeCh*, PS19, and PS19;*ApoeCh* mice. *n *= 4-6 mice/sex/genotype. Data are represented as mean ± SEM. Student’s t-test, unpaired comparison. Two-way ANOVA followed by Tukey’s post hoc tests to examine biologically relevant interactions. Statistical significance is denoted by *p<0.05, **p<0.01, ***p<0.001, ****p<0.0001. Supp. Figure 15: Western blot images of total tau and p-tau in cortical soluble protein fraction of 9-mo PS19 mice. a-b Whole western blot images from left to right: total protein stain, total tau detected by Dako antibody, p-tau detected by AT8, merged blot of Dako- and AT8-detected protein. c-d Whole western blot images from left to right: total protein stain, total tau detected by HT7 antibody, p-tau detected by pThr231, merged blot of HT7- and pThr231-detected protein. Sample genotypes on blots: 1= WT Female, 2= PS19 Female, 3= PS19;*ApoeCh* Female, 4= PS19 Male, 5= PS19;*ApoeCh* Male, 6= PS19 Female, 7= PS19;*ApoeCh* Female, 8= PS19 Male, 9= PS19;*ApoeCh* Male, 10 = WT Male, 11= MAPT KO Female. Red arrows highlight rows of bands of interest. White boxes highlight portions of blots used as represented images in Figure 5k-p. *n *= 4 mice/sex/genotype. Supp. Figure 16: Immunohistochemical analysis of 5 and 9-mo-old WT, *ApoeCh*, PS19, PS19;*ApoeCh* mice. a, c Representative 20x confocal images of the dentate gyrus immunolabeled with IBA1 for microglia (red, a) and GFAP for astrocytes (blue, c). Scale bar = 100µm. In each panel, sex of individual animals is denoted by pink (female) or blue (male) circles. b, d Total volume of IBA1+ (b) and GFAP+ (d) cells per FOV of the dentate gyrus. e, f IBA1+ microglia number per FOV of 5-mo-old (e) and 9-mo-old (f) mice in the dentate gyrus. g Representative whole hemisphere stitched images immunostained for CD68. Scale bar = 500µm. h Percent CD68+ area covered in the whole hemisphere brain sections of 9-mo-old WT, *ApoeCh, *PS19, and PS19*;ApoeCh* mice*.* *n *= 4-6 mice/sex/genotype. Data are represented as mean ± SEM. Student’s t-test, unpaired. Two-way ANOVA followed by Tukey’s post hoc tests to examine biologically relevant interactions. Statistical significance is denoted by *p<0.05, **p<0.01, ***p<0.001, ****p<0.0001. Supp. Figure 17: Cell segmentation and annotation in PS19;*ApoeCh* spatial proteomics data. a Representative images of cell segmentation (teal outline) in dentate gyrus, choroid plexus, and cortex for PS19;*ApoeCh* spatial proteomics dataset. DAPI nuclear stain is shown in light grey. White dotted line demarcates anatomical landmarks for dentate gyrus. Scale bar = 50 µm. b CELESTA cell types in XY space for 11 hemibrains (*n *= 3/genotype, except *n *= 2 for *ApoeCh*). Supp. Figure 18: Cell segmentation and annotation in PS19;*ApoeCh* spatial transcriptomics data. a Representative images of cell segmentation (teal outline) in cortex, dentate gyrus, and white matter tracts for PS19;*ApoeCh* spatial transcriptomics dataset. DAPI nuclear stain (light grey), histone marker (green). White dotted line demarcates anatomical landmarks for dentate gyrus, white matter tracts, and CA1 of hippocampus. Scale bar = 50 µm. b 40 clusters in XY space for all 11 sections (*n *= 3/genotype, except *n *= 2 for *ApoeCh*). Supp. Figure 19: Distribution of cell types across genotypes in PS19;*ApoeCh* dataset. a-b Data quality is comparable across samples. a Number of unique transcripts per cell, grouped by sample. b Number of unique genes (features) per cell, grouped by sample. c UMAP of cells from PS19;*ApoeCh* spatial transcriptomics dataset split by genotype (PS19;*ApoeCh*, PS19, *ApoeCh*, and WT). DAM (dark green) cluster is present in PS19 genotypes, but not in PS19;*ApoeCh*, *ApoeCh*, or WT genotypes. d Heatmap of the top 5 marker genes per major cell type. DGE analysis was performed between each major cell type compared to all other major cell types to identify the top 5 genes expressed for each major cell type. e Stacked bar plot of each of the 40 clusters, split by genotype. Clusters are grouped by their major cell type (e.g., Astrocyte 1-3 and DAA are grouped together) and plotted in order from largest to smallest cluster. Supp. Figure 20: Differential gene expression analysis between PS19 vs. WT and PS19;*ApoeCh* vs. PS19 across major cell types and all cell types. a Volcano plots of DEGs between PS19 and WT for all clusters. Because DAA and DAM clusters are present in very low numbers in WT mice, DAA and astrocytes are combined into “All astrocytes”, and DAM and microglia are combined into “All microglia”. b Volcano plots of DEGs between PS19;*ApoeCh* and PS19 for all 40 clusters. c Volcano plots of DEGs between PS19 and WT for each major cell type. All astrocytes and all microglia are grouped together. Supp. Figure 21: Differential gene expression between PSEN1-E280A;APOECh vs. PSEN1-E280A in human snRNA-seq data. a UMAP of integrated human snRNA-seq data comprising 13,643 cells by 975 genes. Cell type annotations were derived from metadata from the publicly available datasets. b Heatmap of the top 5 marker genes in each cell type. c DEGs between PSEN1-E280A;APOE3Ch vs. PSEN1-E280A in astrocytes. d Scatterplot of log-2 fold-change of the average expression between the two groups for all statistically significant genes (p_adj_ < 0.05) in the human comparison (PSEN1-E280A;APOE3Ch vs. PSEN1-E280A; x-axis) vs. 5xFAD comparison (5xFAD;*ApoeCh* vs. 5xFAD; y-axis) in all microglia. Point color represents direction of correlation; positive (blue) and negative (yellow). Regression line in blue. e Scatterplot of log2FC of average expression for human comparison vs. PS19 comparison (PS19;*ApoeCh* vs. PS19; y-axis) in all microglia. d-e While the magnitude of difference appears greater for the human data, it should be noted that the raw transcript counts obtained from imaging-based single-cell spatial transcriptomics (hybridization-based) are on a different scale from those obtained from snRNA-seq (sequencing-based. Thus, interpretation is limited to the direction and statistical significance of these changes. All positively correlated genes (blue) from d and e are listed in the intersections of the Venn diagram in Fig. 9k. Supp. Figure 22: Unbiased proteomics show differential effects of *ApoeCh* in 5xFAD versus PS19 mice. a-d These results complement Fig. 10g-j by presenting the opposite regulation patterns. a Pathway analysis of all significantly downregulated proteins in the 5xFAD;*ApoeCh* vs. 5xFAD comparison. b Pathway analysis of all significantly upregulated proteins in the PS19;*ApoeCh *vs. PS19 comparison. c Protein-protein interaction (PPI) network of all significantly downregulated proteins in the 5xFAD;*ApoeCh* vs. 5xFAD comparison. The thickness of the line connecting two nodes indicates the degree of confidence in the prediction of the PPI. d PPI network of all significantly upregulated proteins in the PS19;*ApoeCh* vs. PS19 comparison.

## Data Availability

Protocols, data, and results are available via the AD Knowledge Portal (https://adknowledgeportal.synapse.org). The AD Knowledge Portal is a platform for accessing data, analyses, and tools generated by the Accelerating Medicines Partnership (AMP-AD) Target Discovery Program and other National Institute on Aging (NIA)-supported programs to enable open-science practices and accelerate translational learning. The data, analyses and tools are shared early in the research cycle without a publication embargo on secondary use. Data is available for general research use according to the following requirements for data access and data attribution (https://adknowledgeportal.org/DataAccess/Instructions). Data can be accessed in an interactive matter at UCI Mouse Mind Explorer (admodelexplorer.org). Single-cell spatial transcriptomics and proteomics datasets are available on the Dryad data repository (10.5061/dryad.m63xsj4ck). Each dataset is provided as a .RDS file which includes raw and corrected counts for the RNA data and mean fluorescent intensity for the protein data, along with comprehensive metadata. Metadata include mouse genotype, sample ID, cell type annotations, sex (for PS19;*ApoeCh* dataset, as sexes were mixed), and X–Y coordinates of each cell. Results from differential gene expression analysis are also available through Dryad as .CSV files. Bulk proteomics analysis results are available through Synapse (https://www.synapse.org/Synapse:syn63940117). The *ApoeCh* model is available from The Jackson Laboratory (Stock # 039301) without restrictions on its use by both academic and commercial users. The content is solely the responsibility of the authors and does not necessarily represent the official view of the National Institutes of Health.

## Abbreviations

AD: Alzheimer’s disease
ADAD: Autosomal Dominant Alzheimer's Disease
APOE: Apolipoprotein E
ApoeCh: Apoe Christchurch
APP: Amyloid precursor protein
Aβ: Amyloid-beta
CNS: Central nervous system
DAA: Disease-associated astrocyte
DAM: Disease-associated microglia
DEGs: Differentially expressed genes
DGE: Differential gene expression
EOAD: Early-Onset Alzheimer's Disease
hAPOE: Human APOE protein
HSPGs: Heparan sulfate proteoglycans
LDLR: Low-density lipid receptor
LOAD: Late-onset Alzheimer’s Disease
LRP1: Lipoprotein-related receptor 1
mAPOE: Mouse APOE protein
MERFISH: Multiplexed error-robust fluorescence in situ hybridization
NfL: Neurofilament light chain
PBS: Phosphate buffered saline
PFA: Paraformaldehyde
PSEN: Presenilin
UMAP: Uniform Manifold Approximation and Projection
WT: Wild-type
