# Supplementary material for: APOE Christchurch enhances a disease-associated microglial response to plaque but suppresses response to tau pathology
Source: Mol Neurodegener. 2025 Jan 22;20:9. doi: 10.1186/s13024-024-00793-x (PMC11752804; doi:10.1186/s13024-024-00793-x)
Supplement: Supplementary file 1 — Supplementary Material 1. Supp. Table 1 - Location of potential off-target sites for crRNA TMF1648 on mouse chromosome 7. The desired target site within Apoe locus is listed, plus the sequence of each of the 12 potential off-target sites on mouse chromosome 7 (GRCm38/mm10 nucleotide numbering). Green text denotes the 11-nucleotide seed region proximal to the PAM site. Mismatches to the guide are indicated by lowercase bold letters and the number of mismatches (including the number within the seed region) is shown. No difference was found in sequence between the C57BL/6J WT and Apoeem1Aduci alleles at the six potential off-target sites analyzed (Supplementary Fig. 1b). [file 13024_2024_793_MOESM1_ESM.pdf]

Supplemental Table 1 - Location of potential off-target sites for crRNA TMF1648 on mouse chromosome 7

Genomic DNA (11 base seed)	Position	Strand	Mismatches (in seed)	Nearest gene	Off-target code	Analyzed ?
GCACAGAGGAGATACGGGCG|CGG	19696877	-	0	***Apoe* exon 4 - correct target**		
GCACAGAGcAGATgaGGGaG|GGG	45859181	-	4 (3)	*Grin2d* - intron 2	**A**	**YES**
GgACAaAGGAGATACcGGCc|TGG	51963361	-	4 (2)	*Gas2* - intron 7	**B**	**YES**
aCAtAGAGGAaATAaGGGCG|AGG	70552648	-	4 (2)	*Gm35842* lncRNA	**C**	**YES**
GCgCAGAGGAGAcgCGGcCG|GGG	81859324	+	4 (3)	*Tm6sf1 - 5' UTR*	**D**	**YES**
aCACAGAGtAGATACtGGtG|TGG	88598839	+	4 (2)	intergenic - non-conserved	**E**	no
GtACAGAGGAGATACtGGgG|TGG	100149385	-	3 (2)	intergenic - non-conserved	**F**	no
GCACAGAGGAGATggGtGgG|GGG	122836707	-	4 (4)	intergenic - non-conserved	**G**	no
GgAgAGAGGAGATAgGGGCt|AGG	125326739	+	4 (2)	*4933440M02Rik*	**H**	**YES**
aCACAGAGGAGAaAgGGGaG|GGG	132361823	+	4 (3)	*Fgfr2*-217 - intron 2 (2.9Mb)	**I**	**YES**
cCACAGAGGAGAacCGGGCa|AGG	136762831	+	4 (3)	intergenic - non-conserved	**J**	no
GCACAcAGGAGcTACaGGCa|AGG	137604947	-	4 (3)	intergenic - non-conserved	**K**	no
tCACAGAGGAGAcAgGGGaG|GGG	137681077	-	4 (3)	intergenic - non-conserved	**L**	no
