# Supplementary material for: APOE Christchurch enhances a disease-associated microglial response to plaque but suppresses response to tau pathology
Source: Mol Neurodegener. 2025 Jan 22;20:9. doi: 10.1186/s13024-024-00793-x (PMC11752804; doi:10.1186/s13024-024-00793-x)
Supplement: Supplementary file 2 — Supplementary Material 2. Supp. Table 2: Primers for PCR amplification and sequencing for off-target analysis. Forward (For) and reverse (Rev) primer sequences are listed for each potential off-target site. The off-target code corresponds to the panels in Supplementary Fig. 1. [file 13024_2024_793_MOESM2_ESM.pdf]

Supplementary Table 2: Primers for PCR amplification and sequencing for off-target analysis. Forward (For) and reverse (Rev) primer sequences are listed for each potential off-target site. The off-target code corresponds to the panels in **Supplementary Fig. 1**.

Off-target code	Primer Name	Primer Sequence (5' - 3')	Product size	Chromosome 18 locus
A	A For	AACCCATCTCTCATCTTGAACC	616 bp	*Grin2d*, intron 2
	A Rev	ATGGAGCTGAGGCTAGAAGAC		
B	B For	CACAGACTAACGTCCTTAGCAC	945 bp	*Gas2*, intron 7
	B Rev	CGTATCACCAAGGGAGCTATG		
C	C For	CCTCACCAACTTTCCTCTACAC	442 b	*Gm35842*, lncRNA
	C Rev	CAAACTCTCAGGTGGCTGTAAC		
D	D For	CCATTAAAGCAAGCGTCCTG	467 bp	*Tm6sf1*, 5' UTR
	D Rev	GAAGCTACCCAGTCCCAAAG		
H	H For	AGTGTCCACCTATCCTTTATCG	1044 bp	*4933440M02Rik* , lncRNA
	H Rev	TTTCTGGCTCTCAGCACTAC		
I	I For	AGTCCGTACACAAACCCAAG	829 bp	*Fgfr2-217* - intron 2 (2.9Mb)
	I Rev	GCAGCATGGTGGATGAATTTG		
