# Supplementary material for: APOE Christchurch enhances a disease-associated microglial response to plaque but suppresses response to tau pathology
Source: Mol Neurodegener. 2025 Jan 22;20:9. doi: 10.1186/s13024-024-00793-x (PMC11752804; doi:10.1186/s13024-024-00793-x)
Supplement: Supplementary file 3 — Supplementary Material 3. Supp. Figure 1: Sequence of the Apoeem1Aduci (ApoeCh) allele and off-target site analysis for crRNA TMF1648 on mouse chromosome 7. (a) Amino acid (aa) sequence alignment between human and mouse APOE and DNA sequence of the wildtype and ApoeCh alleles in the region of the APOE R136S (R128S in mouse mature APOE). Red-colored nucleotides denote the missense codon while the green nucleotides denote synonymous base changes introduced to prevent recutting of the targeted site. (b) Chromatograms of DNA sequence in wildtype and ApoeCh heterozygous mice, with colored asterisks corresponding to the colored nucleotides in the above sequence. (c-i) Chromatograms of N3F2 wildtype and ApoeCh homozygous offspring at six potential off-target sites on mouse chromosome 7. No difference was found in sequence between the B6J WT and ApoeChhomozygotes at each of the six potential off-target sites analyzed. The black underline denotes the crRNA target sequence while the blue underline denotes the NGG PAM site. Supp. Figure 2: Behavioral analysis of 4- and 12-mo-old WT, ApoeCh, 5xFAD, 5xFAD;ApoeCh mice. a,b Plasma triglyceride (a) and VLDL (b) level in 4 mo WT and ApoeCh HO mice. c,d Weight of 4-mo-old (c) and 12-mo-old (d) mice taken at euthanizing day. e, f Total time mice spent in the center of open field behavioral assay of 4-mo-old (e) and 12-mo-old (f) in the 5 min recording time. g,h Mean velocity mice traveled in the center of the open field in 5 min of 4-mo (g) and 12-mo-old mice (h). i, j Total time 4-mo (i) and 12-mo-old (j) mice spent in open arms of the elevated plus maze behavior test. n = 4-6 mice/sex/genotype. Data are represented as mean ± SEM. Student’s t-test, unpaired. Two-way ANOVA followed by Tukey’s post hoc tests to examine biologically relevant interactions. Statistical significance is denoted by *p<0.05,**p<0.01, ***p<0.001, ****p<0.0001. # denotes trending significance. Supp. Figure 3: Sex-specific differences in pathology in the 5xFAD m [file 13024_2024_793_MOESM3_ESM.pdf]

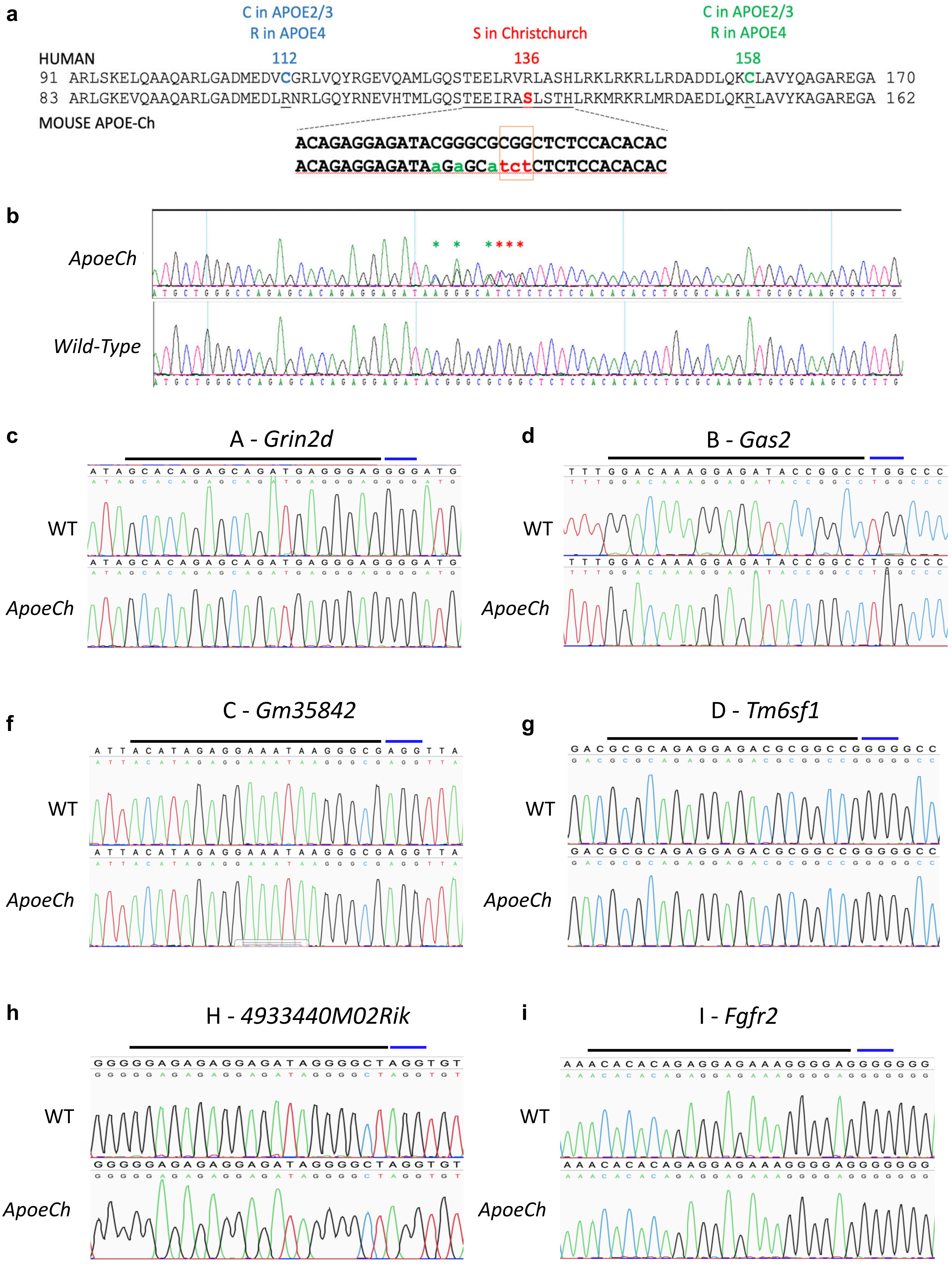

Supplemental Figure 1

Supplemental Figure 2

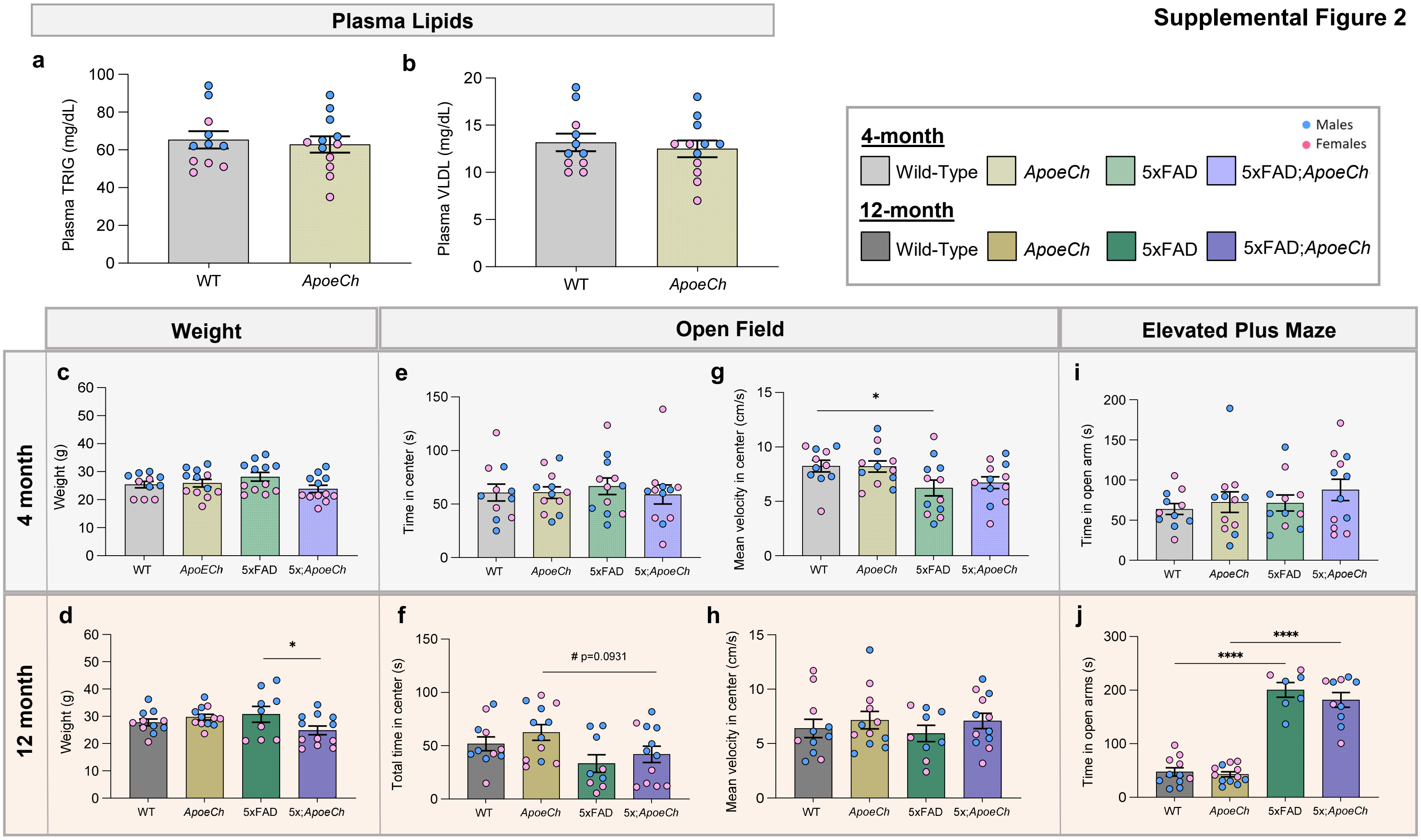

Supplemental Figure 3

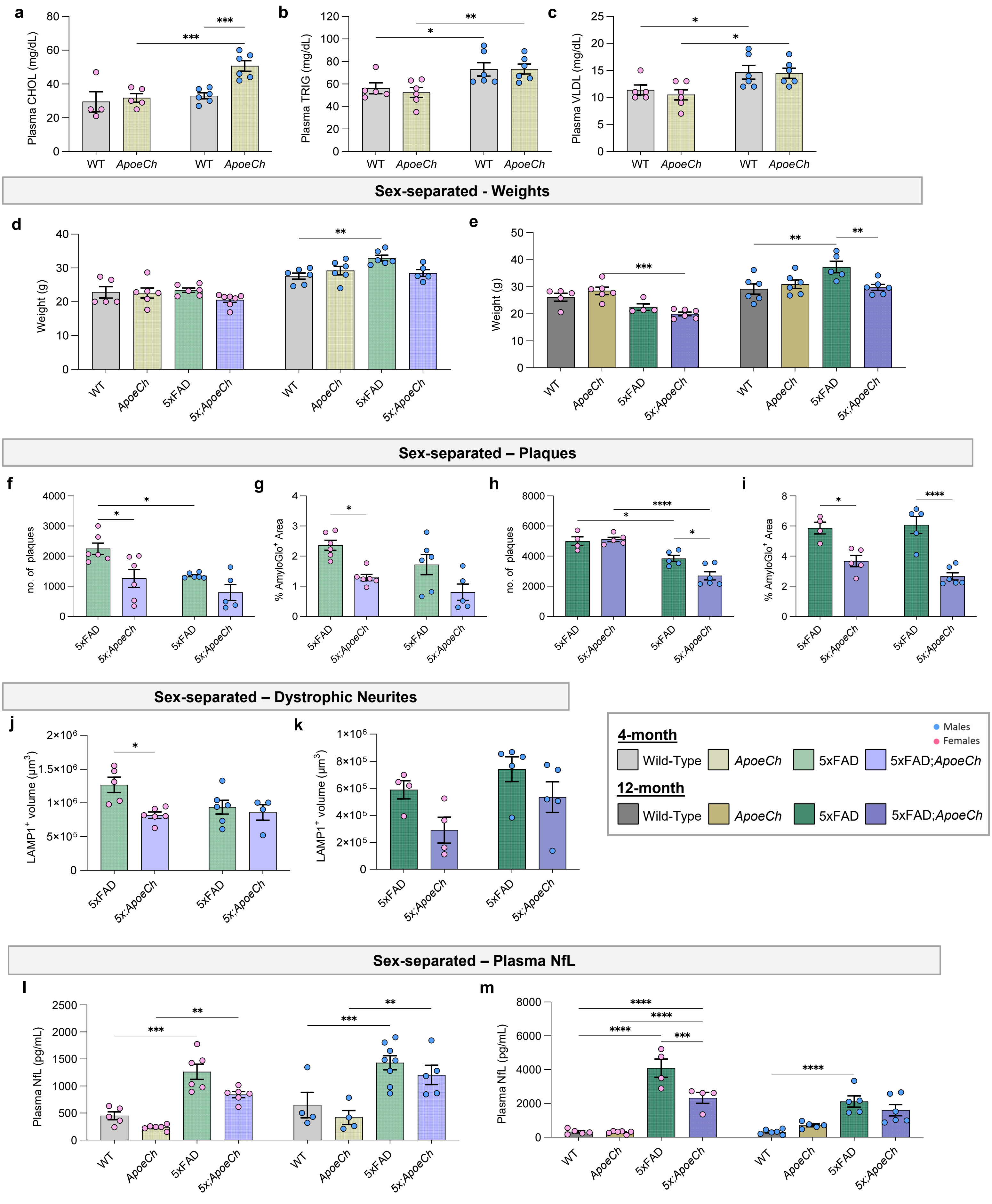

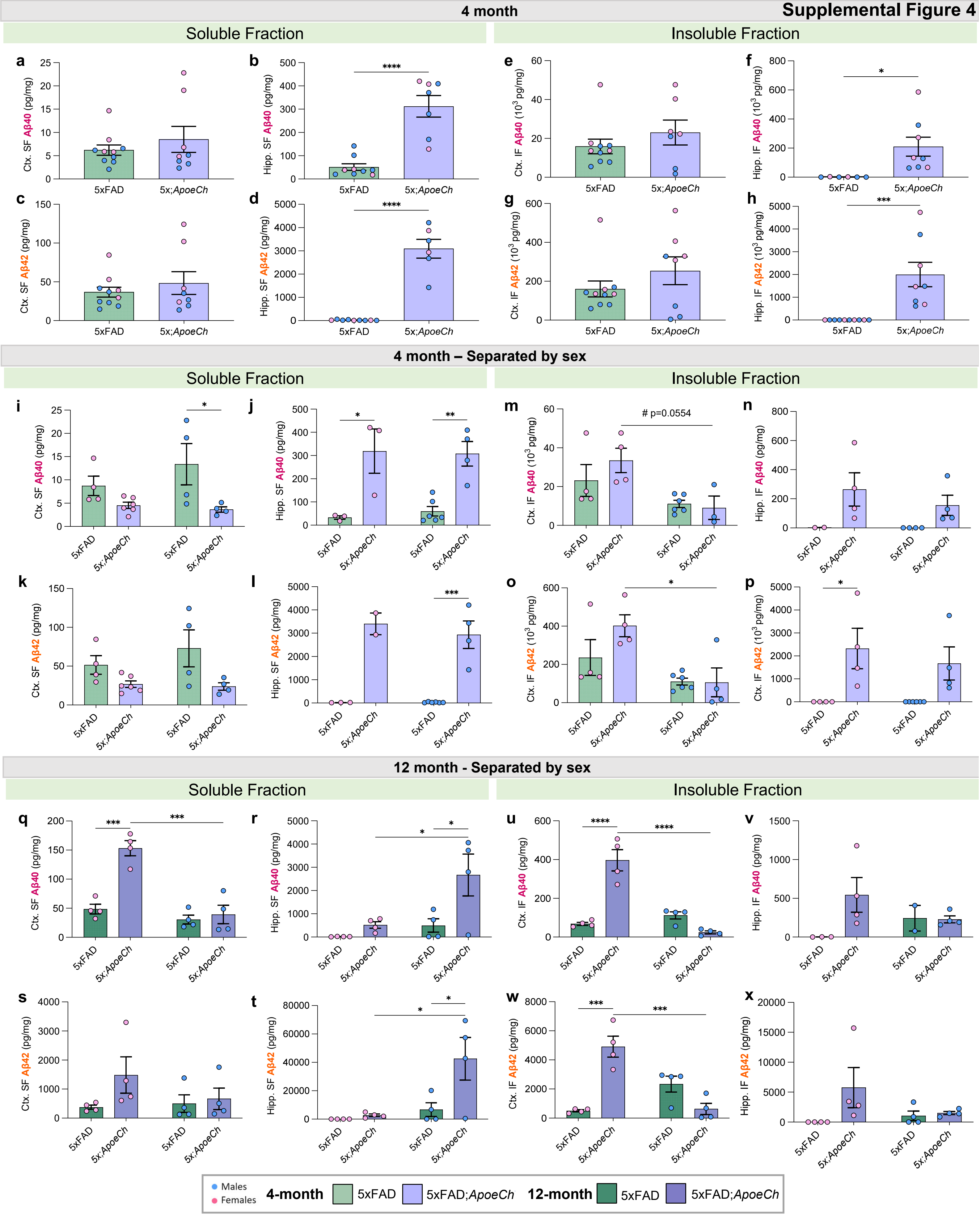

Supplemental Figure 4
4 month
Soluble Fraction
Insoluble Fraction
4 month – Separated by sex
12 month - Separated by sex
Ctx. SF Aβ40 (pg/mg)
Hipp. SF Aβ40 (pg/mg)
Ctx. IF Aβ40 (10^3 pg/mg)
Hipp. IF Aβ40 (10^3 pg/mg)
Ctx. SF Aβ42 (pg/mg)
Hipp. SF Aβ42 (pg/mg)
Ctx. IF Aβ42 (10^3 pg/mg)
Hipp. IF Aβ42 (10^3 pg/mg)
Hipp. IF Aβ40 (pg/mg)
Ctx. IF Aβ40 (pg/mg)
Ctx. IF Aβ42 (pg/mg)
Hipp. IF Aβ42 (pg/mg)
5xFAD
5x;ApoeCh
# p=0.0554
Males
Females
4-month
12-month
5xFAD;ApoeCh

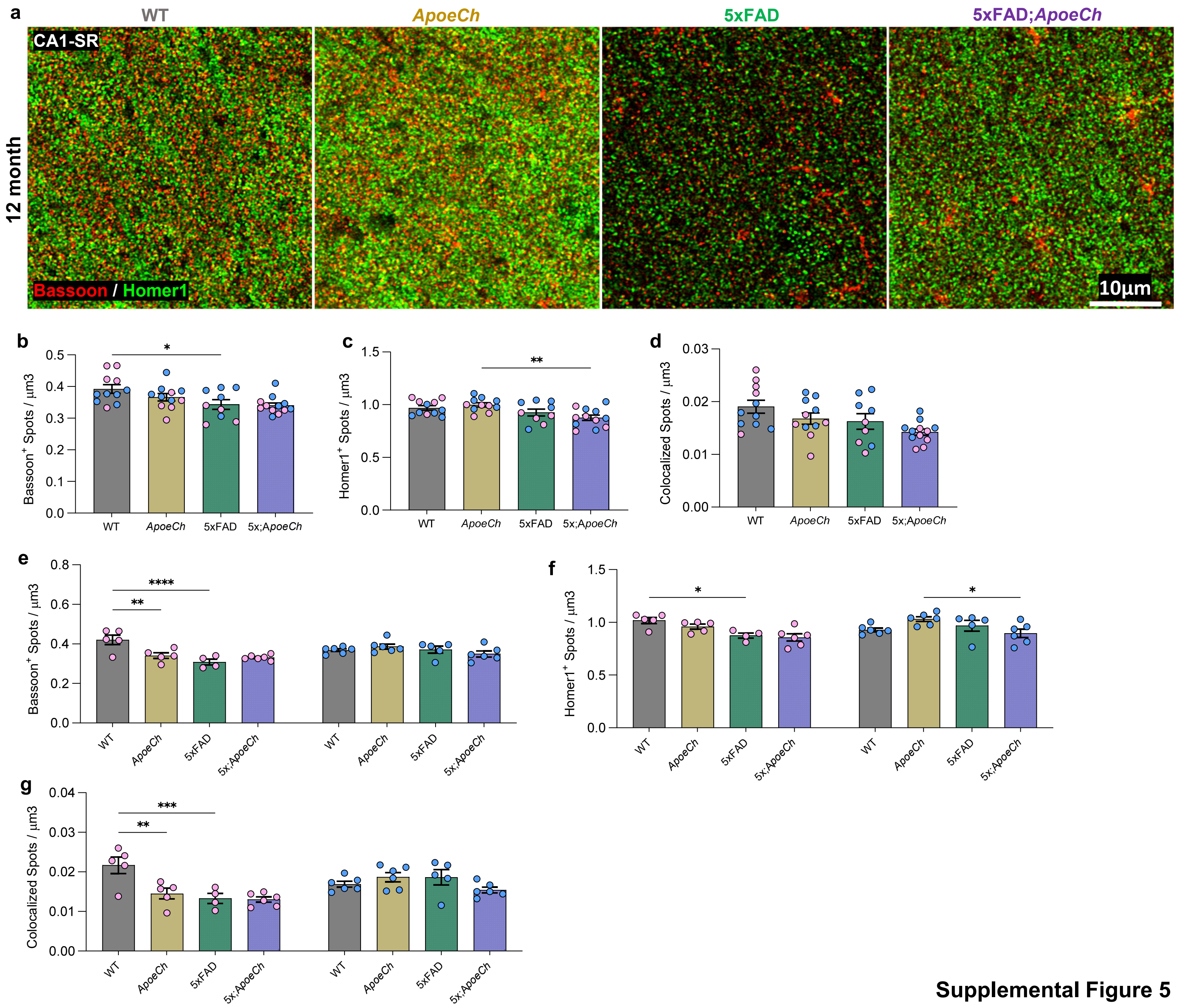

Supplemental Figure 5

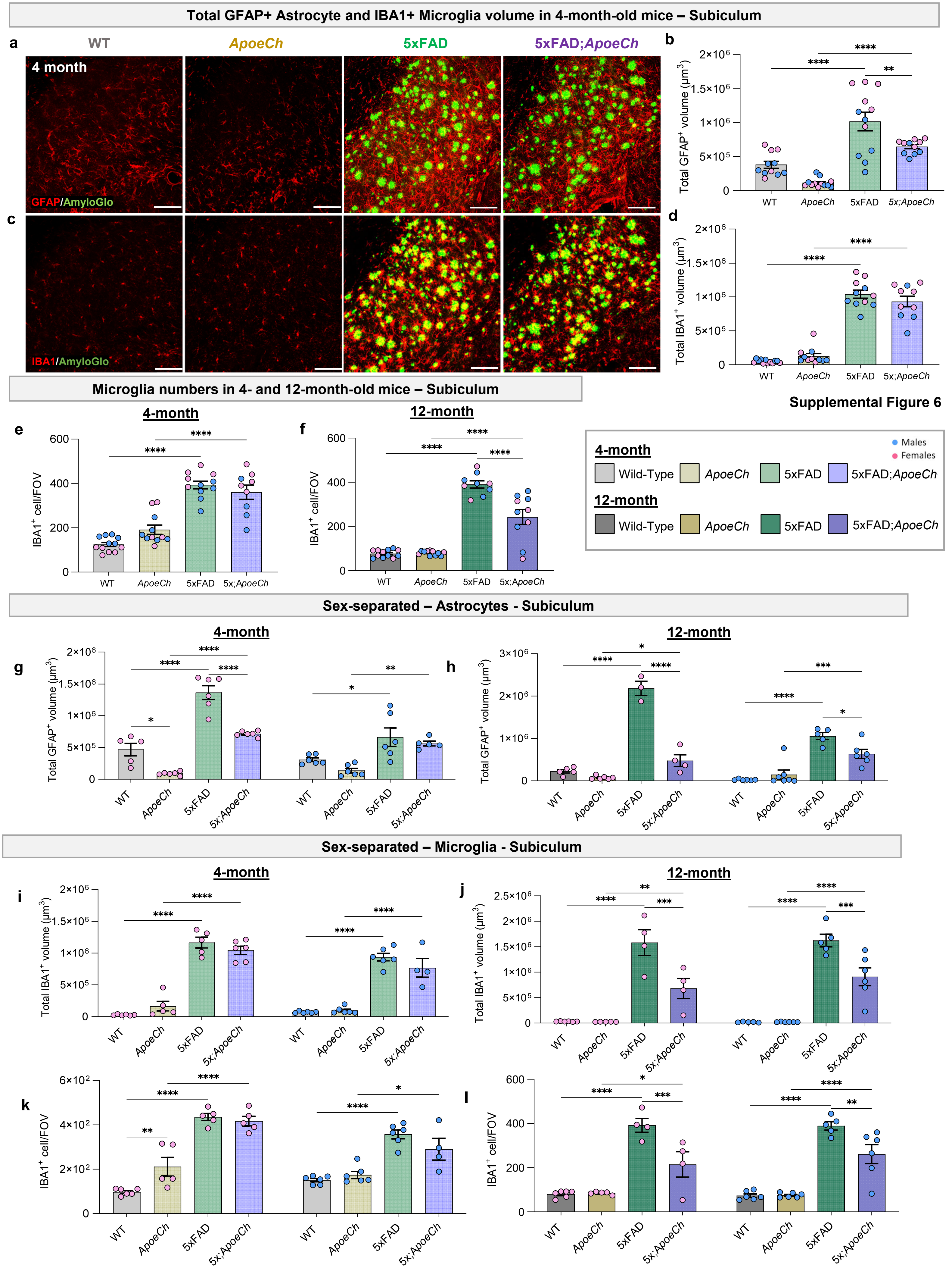

Supplemental Figure 6

Supplemental Figure 7

a Representative images of cell segmentation in cortex, dentate gyrus, and white matter tracts

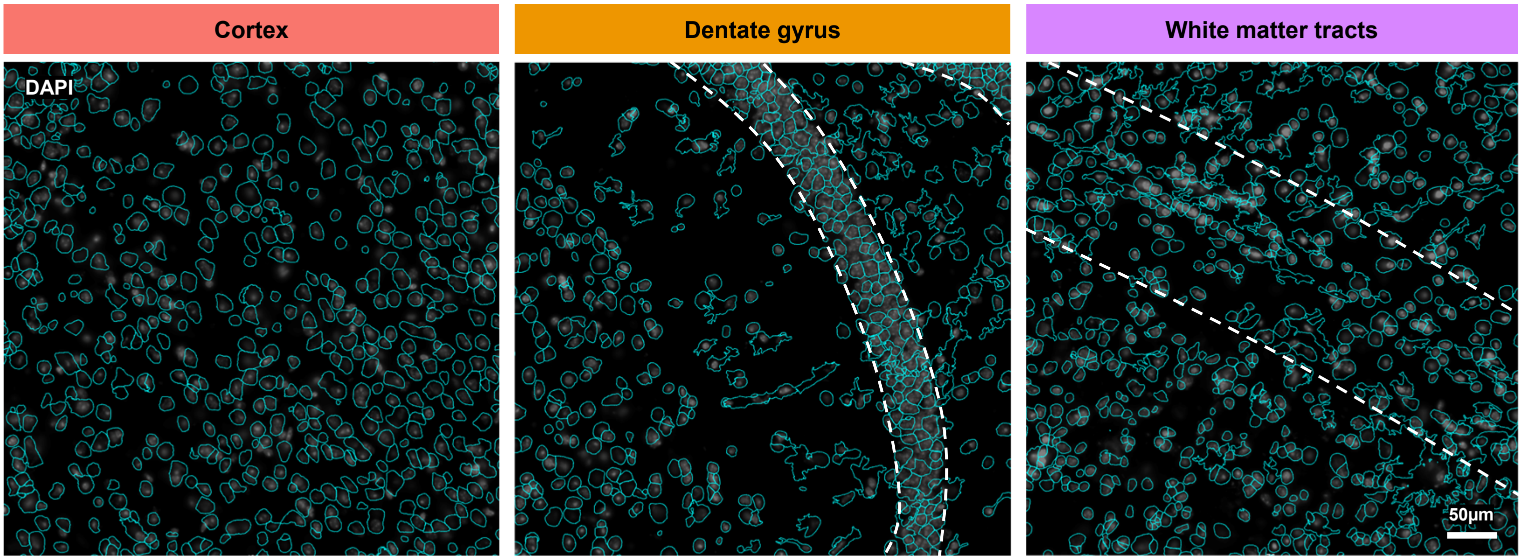

b Cell types in XY space

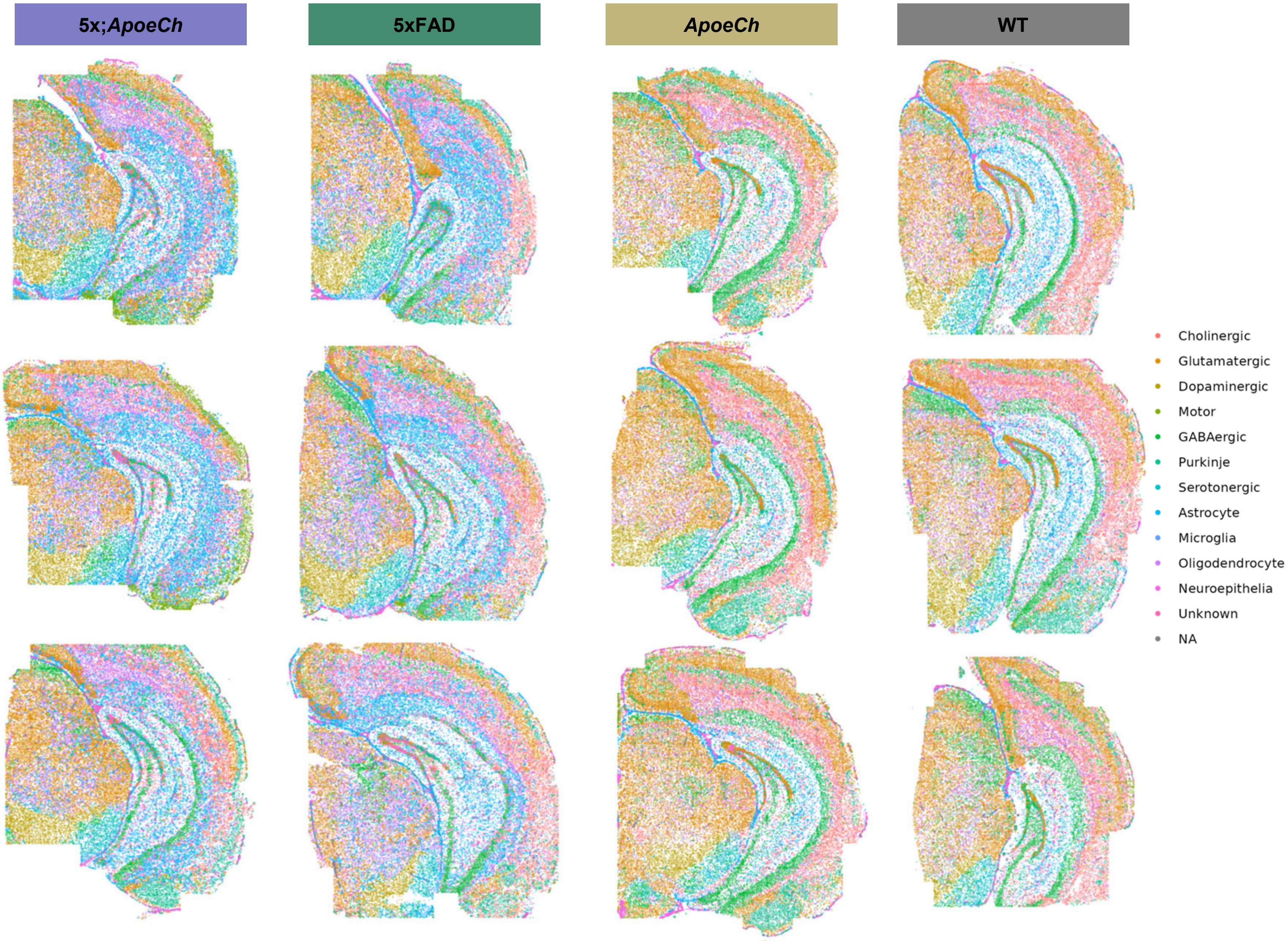

Supplemental Figure 8

a Representative images of cell segmentation in white matter tracts, CA1, and cortex

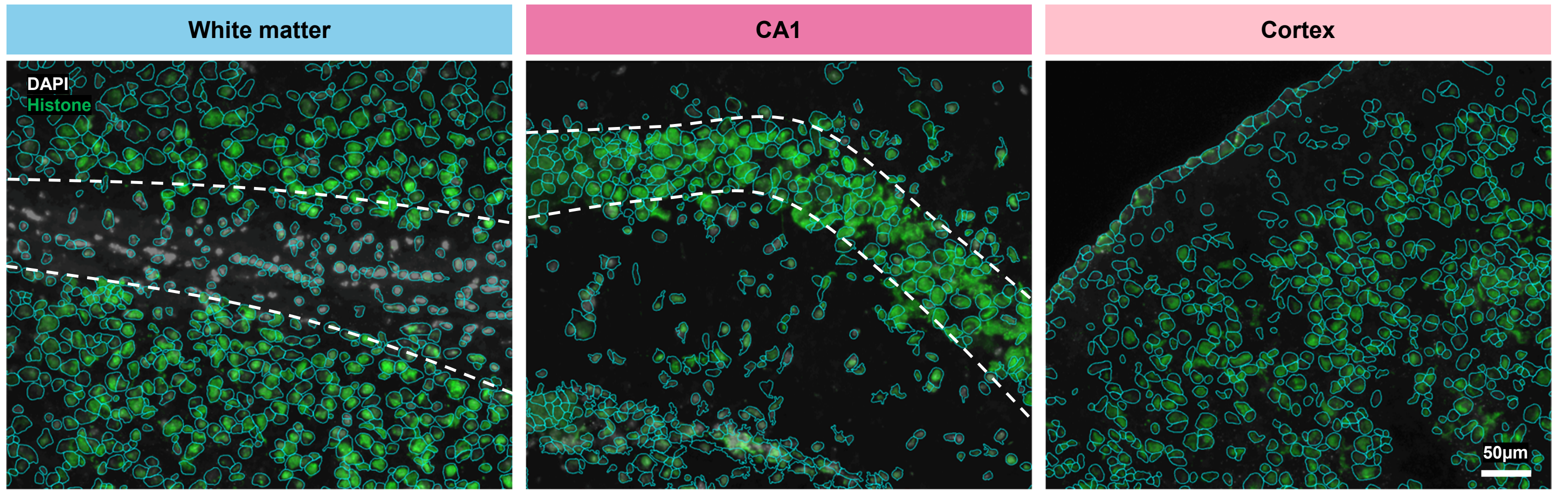

b Cell types in XY space

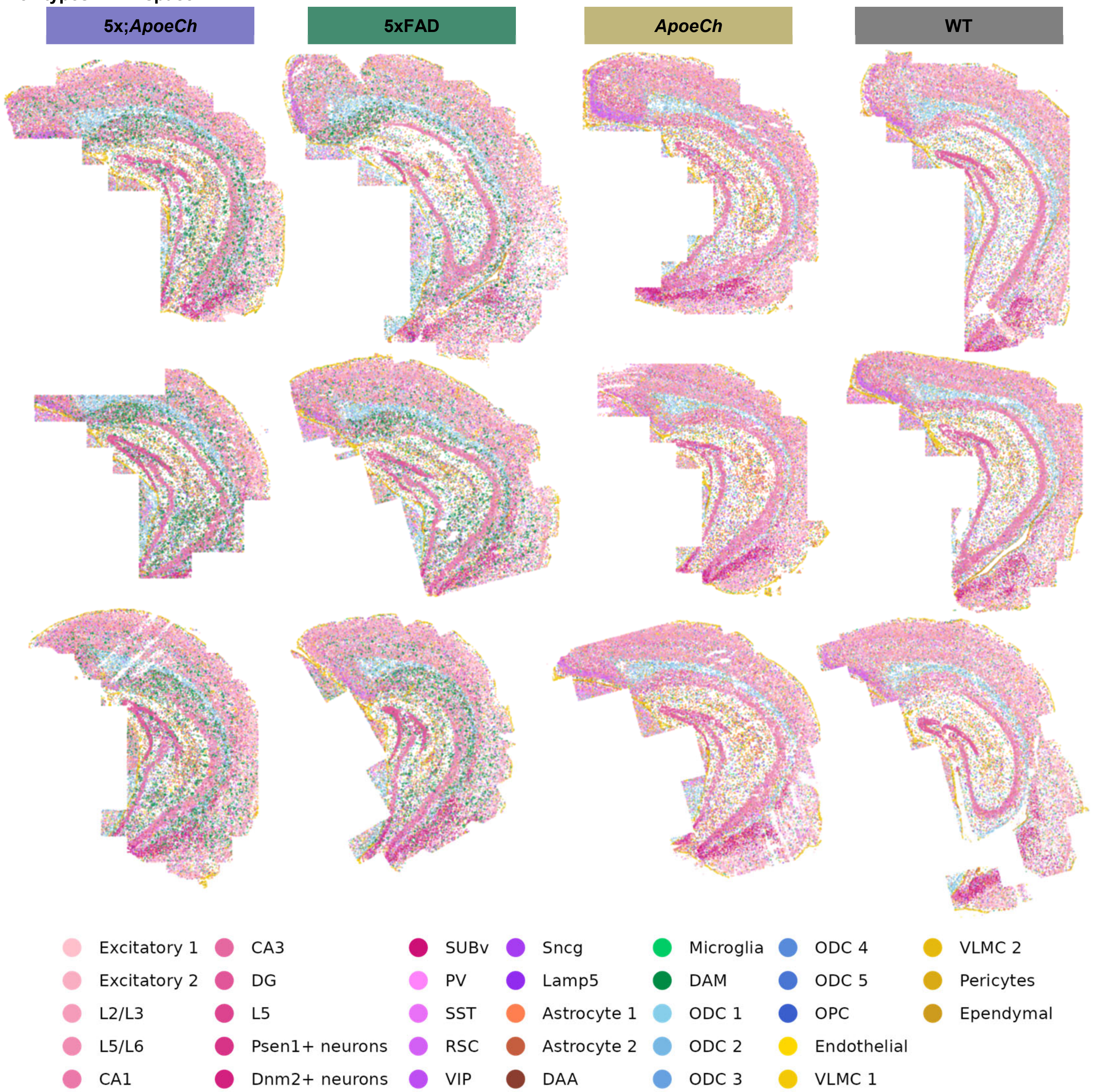

Supplemental Figure 9

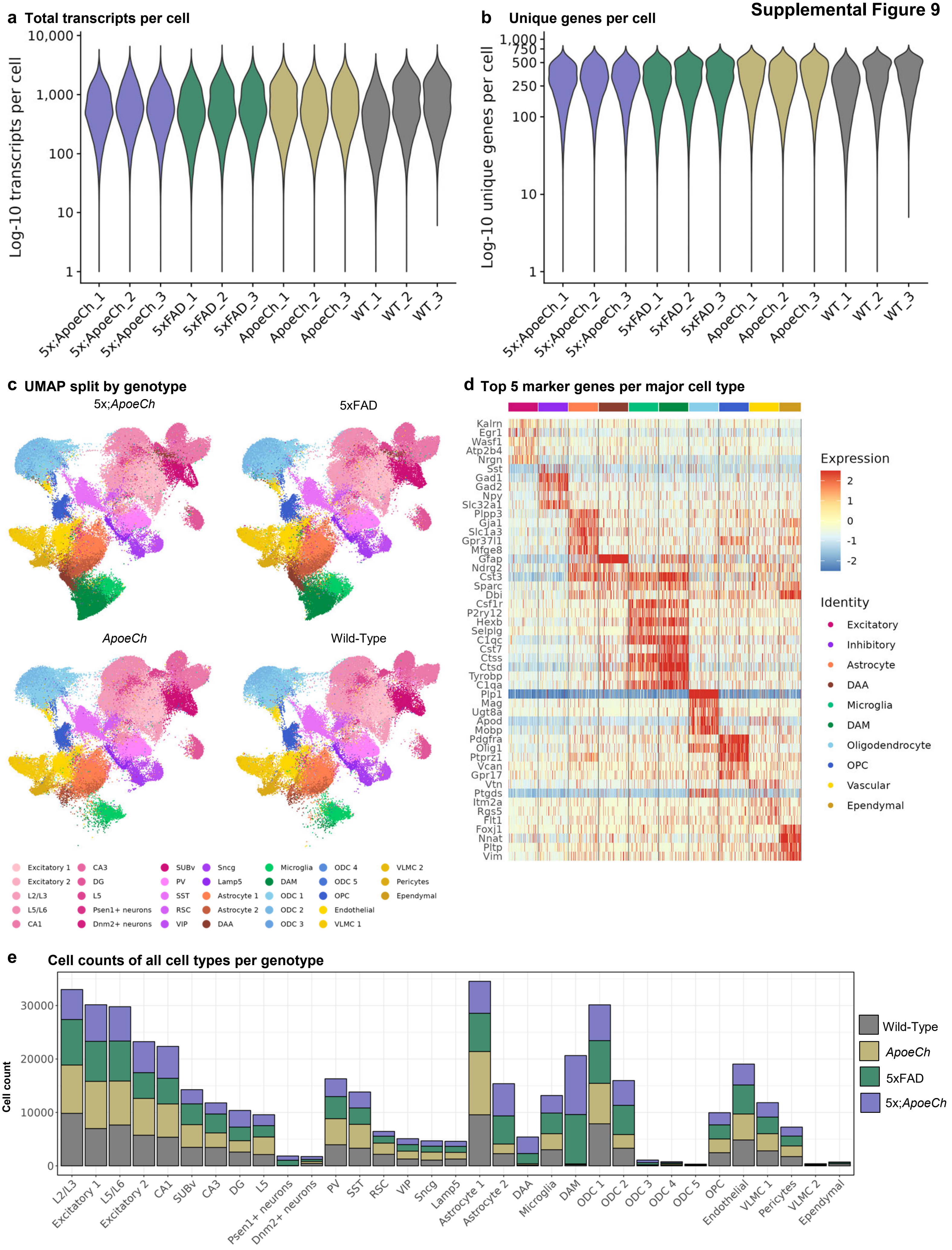

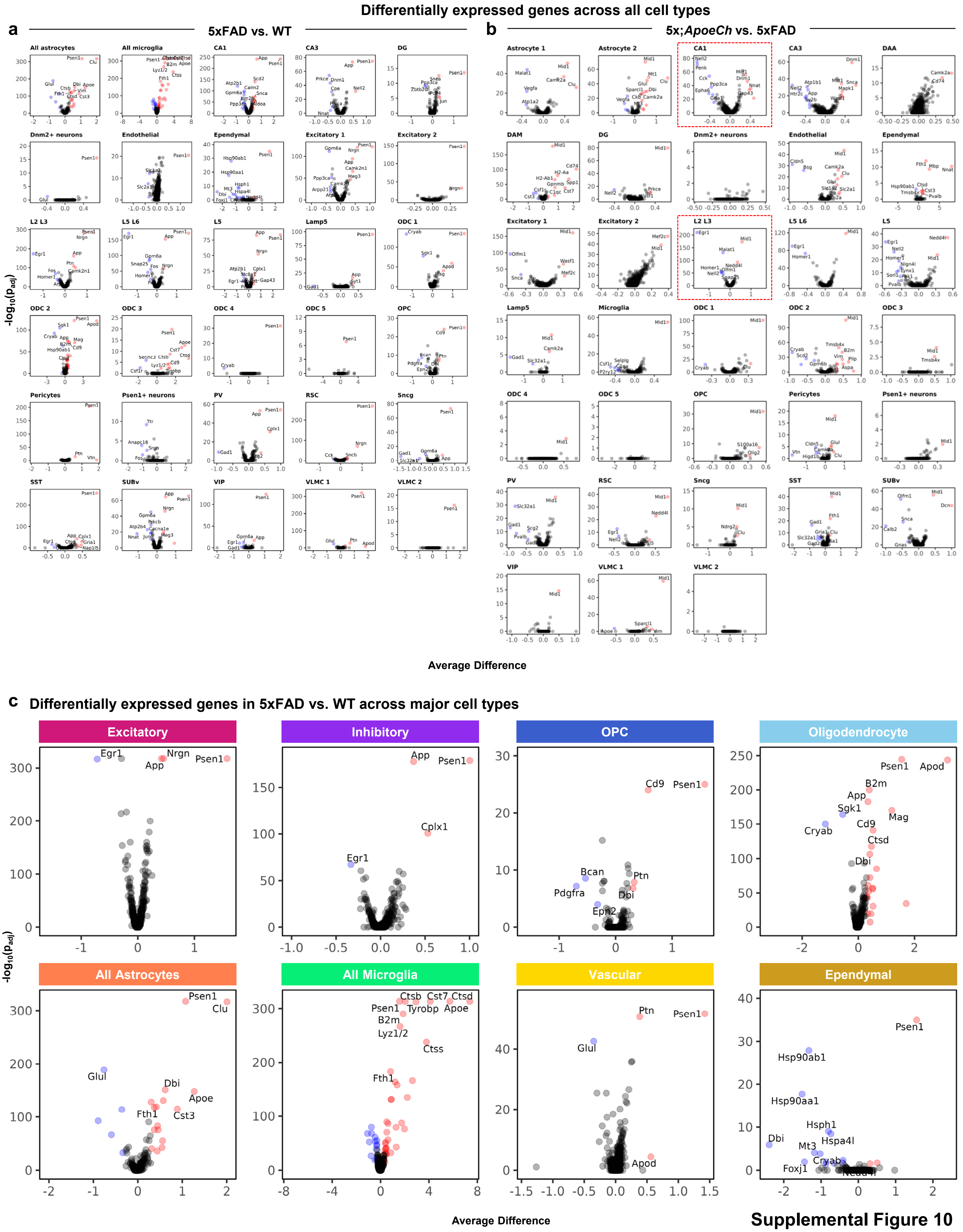

Supplemental Figure 10

a Dentate gyrus, CA1, and L2/L3 in XY space

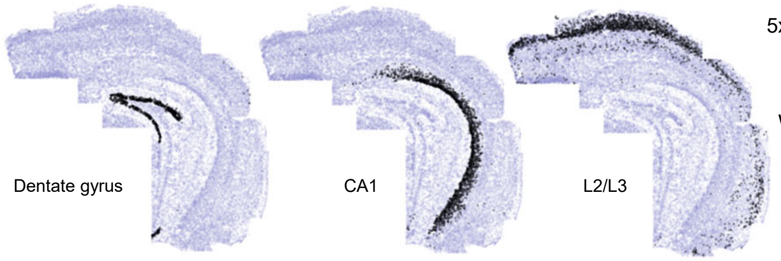

b

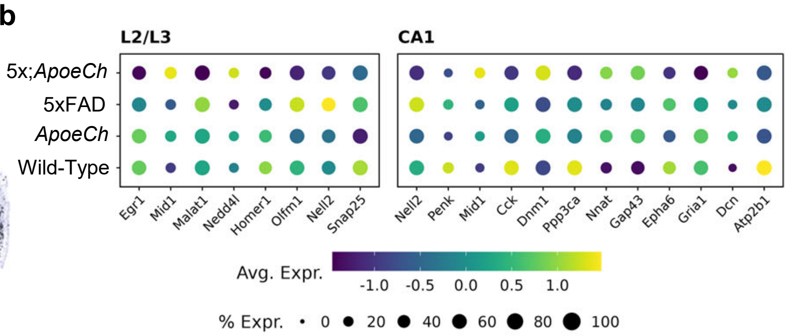

a Feature plots for microglial and DAM marker genes

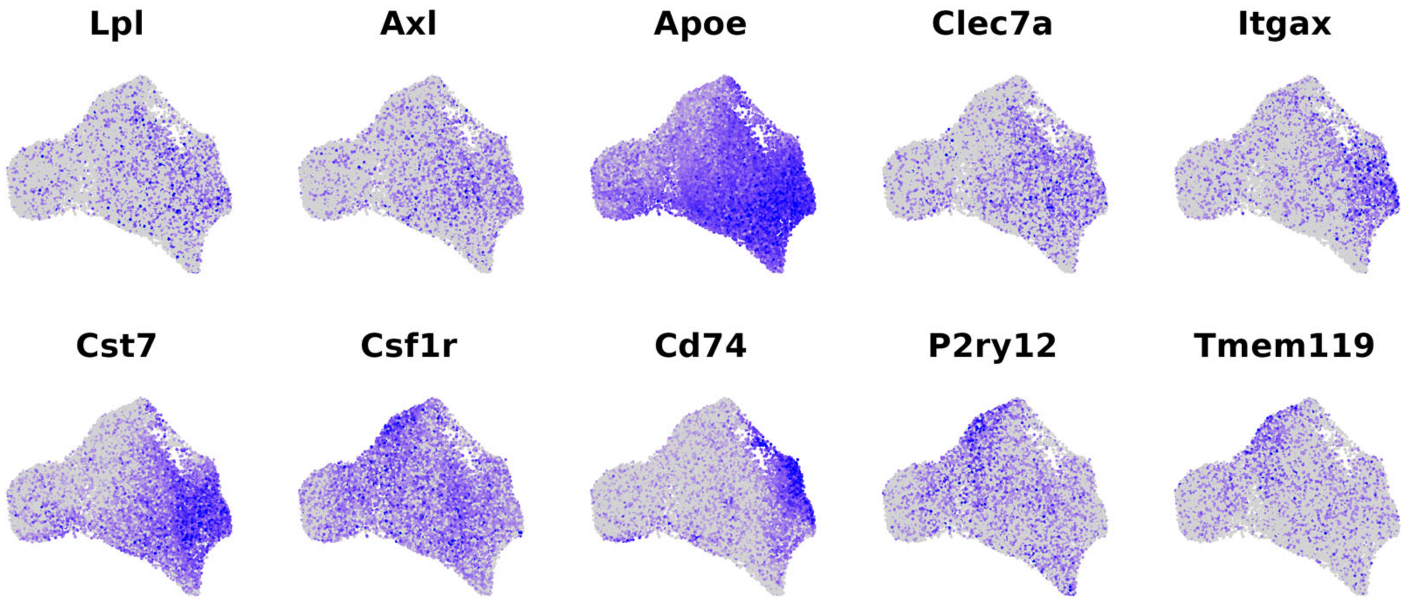

b Cell segmentation of plaque surrounded by microglia

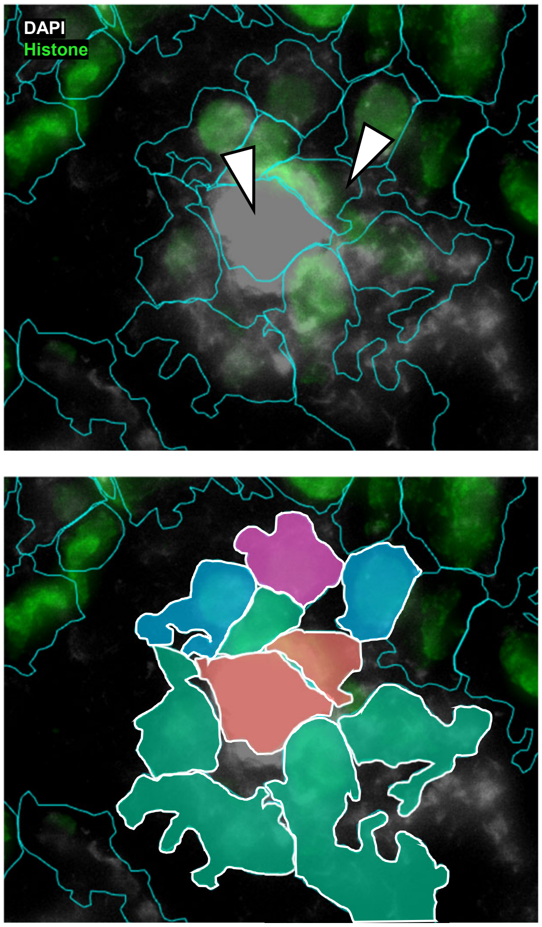

c Heatmap of the top 10 marker genes in each microglial subcluster

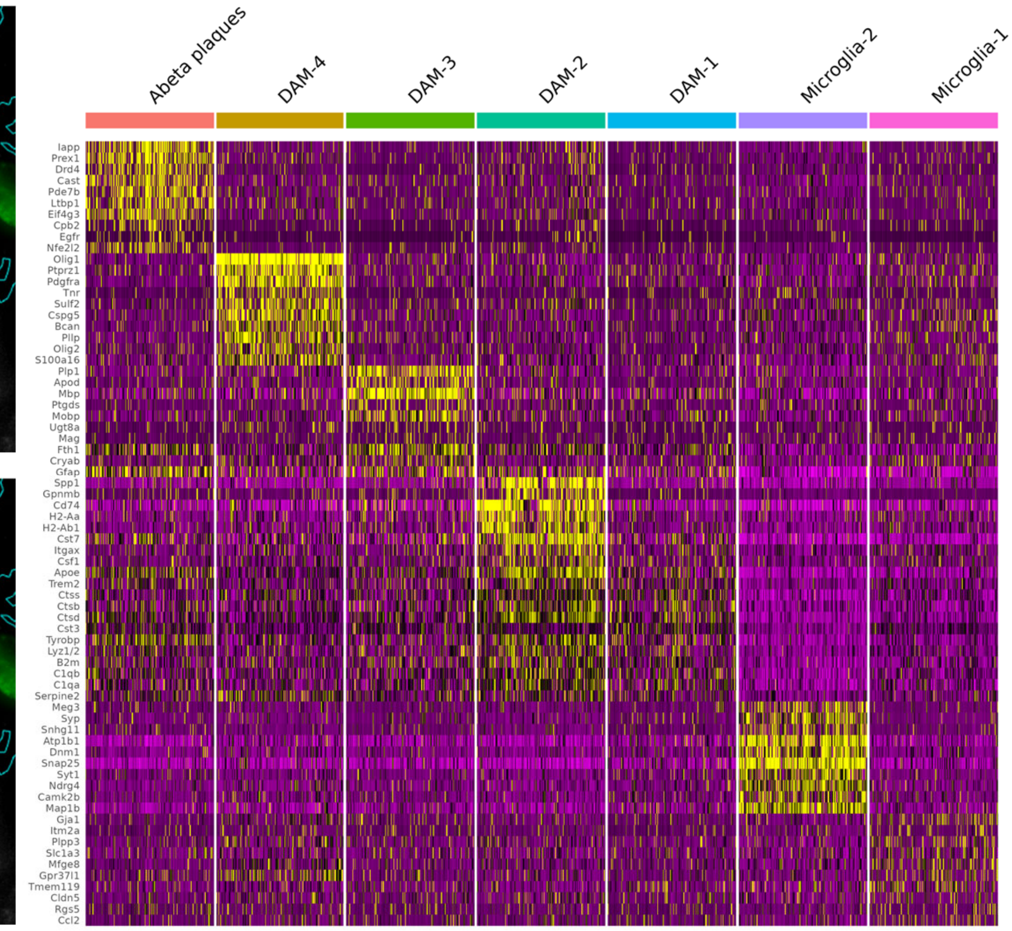

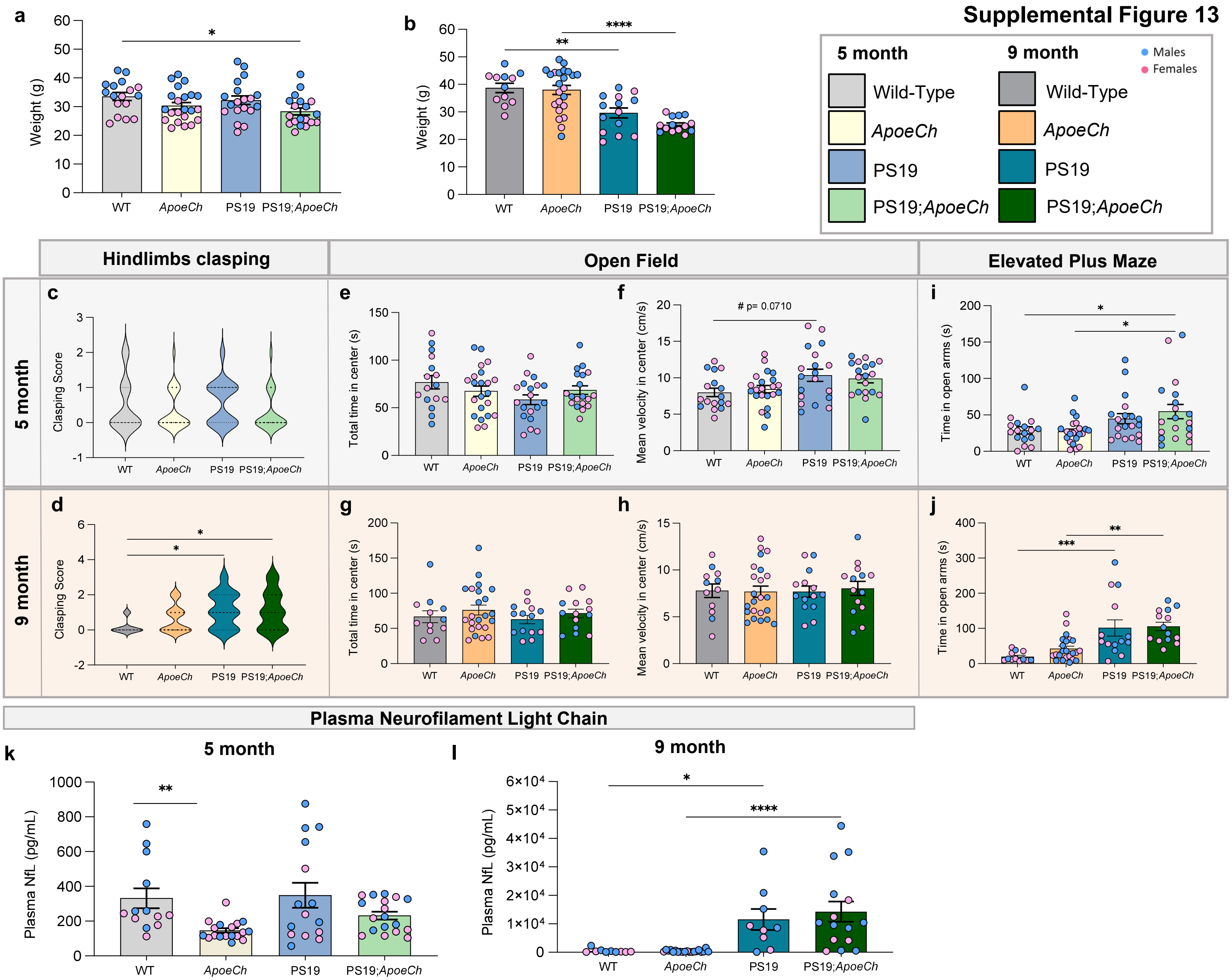

Supplemental Figure 13
a
b
Weight (g)
WT
ApoeCh
PS19
PS19;ApoeCh
5 month
9 month
Males
Females
Wild-Type
ApoeCh
PS19
PS19;ApoeCh
Hindlimbs clasping
Open Field
Elevated Plus Maze
c
d
e
f
g
h
i
j
Clasping Score
Total time in center (s)
Mean velocity in center (cm/s)
# p= 0.0710
Time in open arms (s)
Plasma Neurofilament Light Chain
k
l
Plasma NfL (pg/mL)

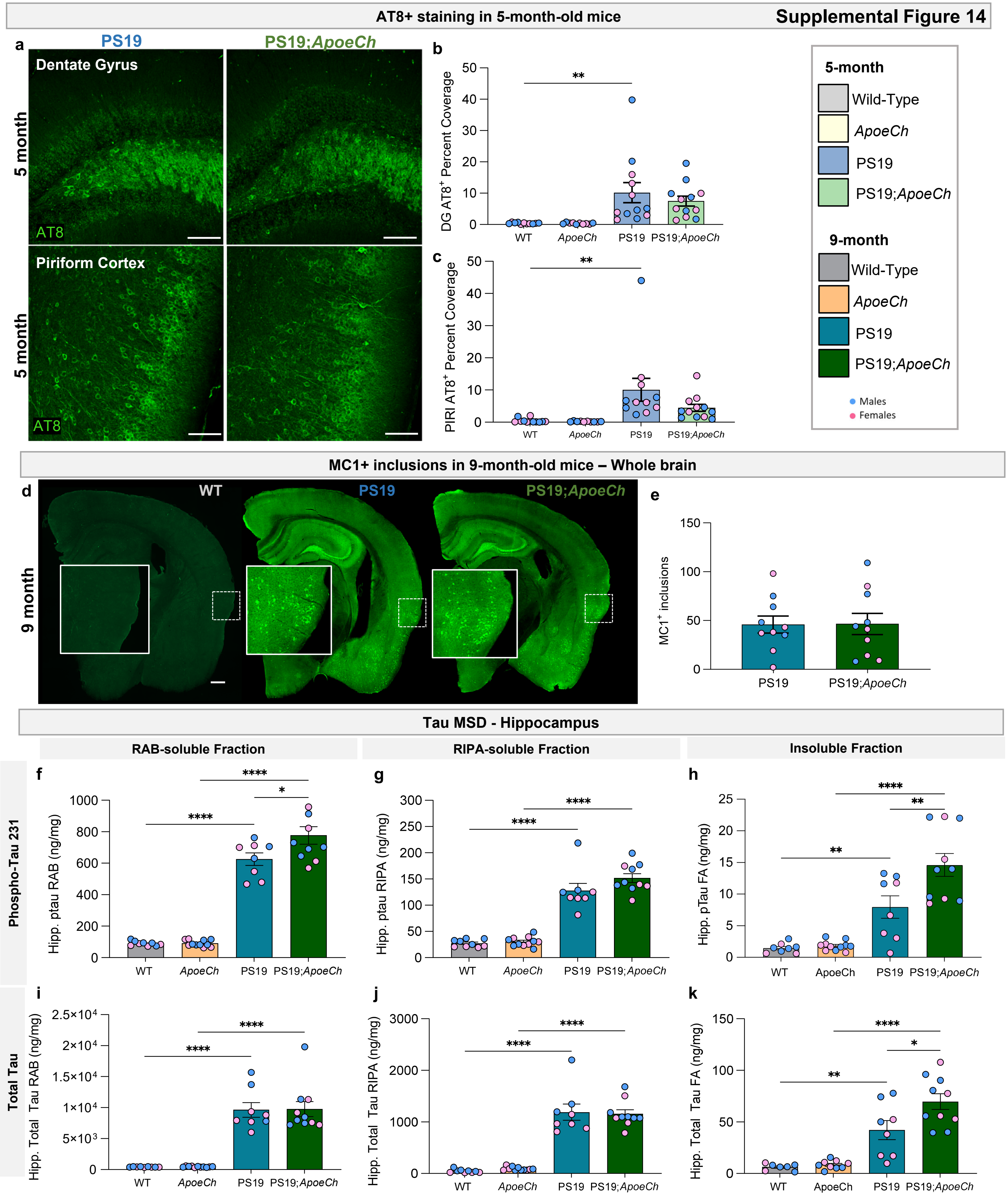

Supplemental Figure 14
AT8+ staining in 5-month-old mice
a
PS19
PS19;ApoeCh
Dentate Gyrus
5 month
AT8
Piriform Cortex
5 month
AT8
b
DG AT8+ Percent Coverage
**
WT
ApoeCh
PS19
PS19;ApoeCh
c
PIRI AT8+ Percent Coverage
**
WT
ApoeCh
PS19
PS19;ApoeCh
5-month
Wild-Type
ApoeCh
PS19
PS19;ApoeCh
9-month
Wild-Type
ApoeCh
PS19
PS19;ApoeCh
Males
Females
MC1+ inclusions in 9-month-old mice – Whole brain
d
WT
PS19
PS19;ApoeCh
9 month
e
MC1+ inclusions
PS19
PS19;ApoeCh
Tau MSD - Hippocampus
RAB-soluble Fraction
RIPA-soluble Fraction
Insoluble Fraction
Phospho-Tau 231
f
Hipp. ptau RAB (ng/mg)
g
Hipp. ptau RIPA (ng/mg)
h
Hipp. pTau FA (ng/mg)
Total Tau
i
Hipp. Total Tau RAB (ng/mg)
j
Hipp. Total Tau RIPA (ng/mg)
k
Hipp. Total Tau FA (ng/mg)
WT
ApoeCh
PS19
PS19;ApoeCh

Supplemental Figure 15

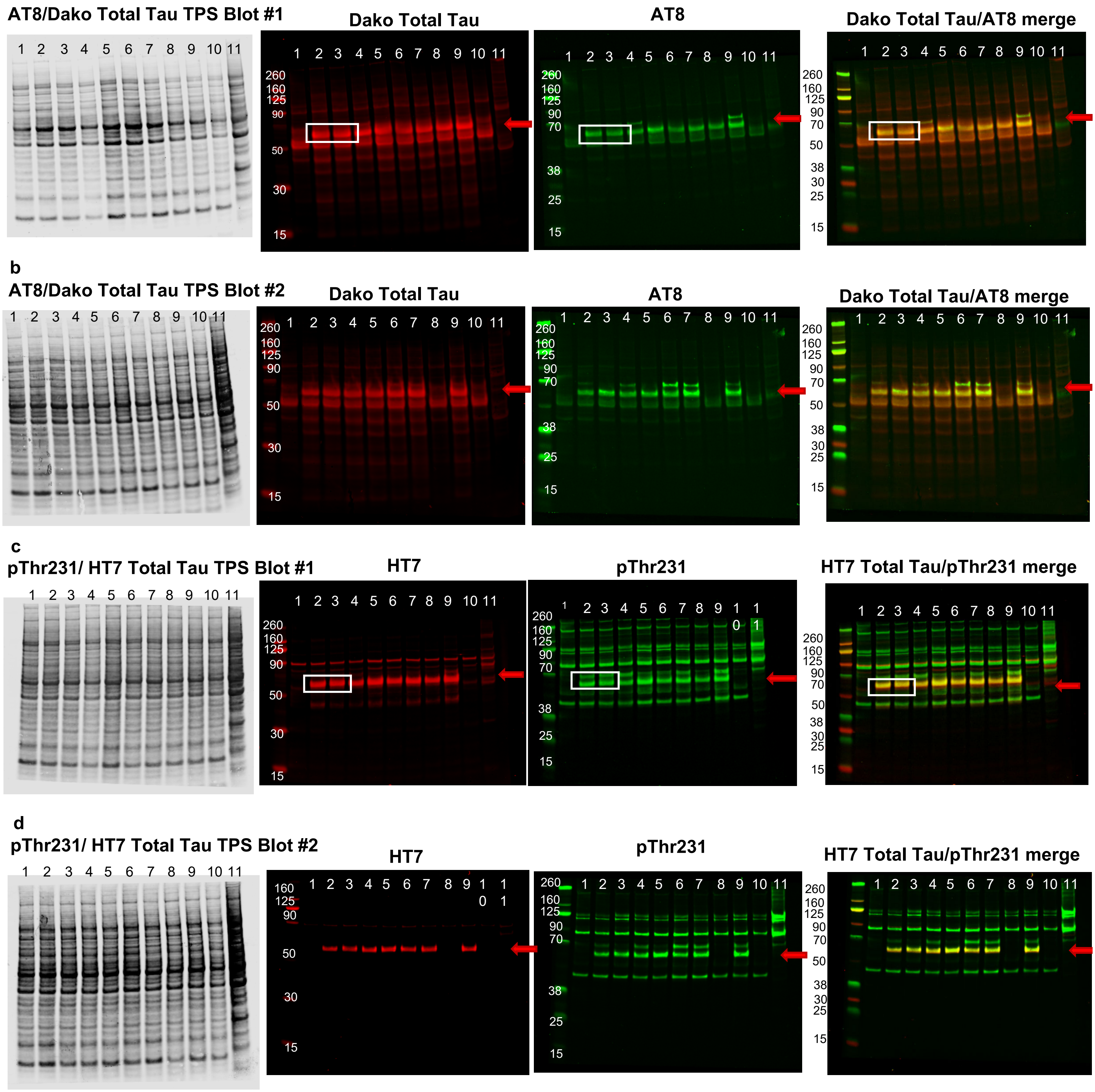

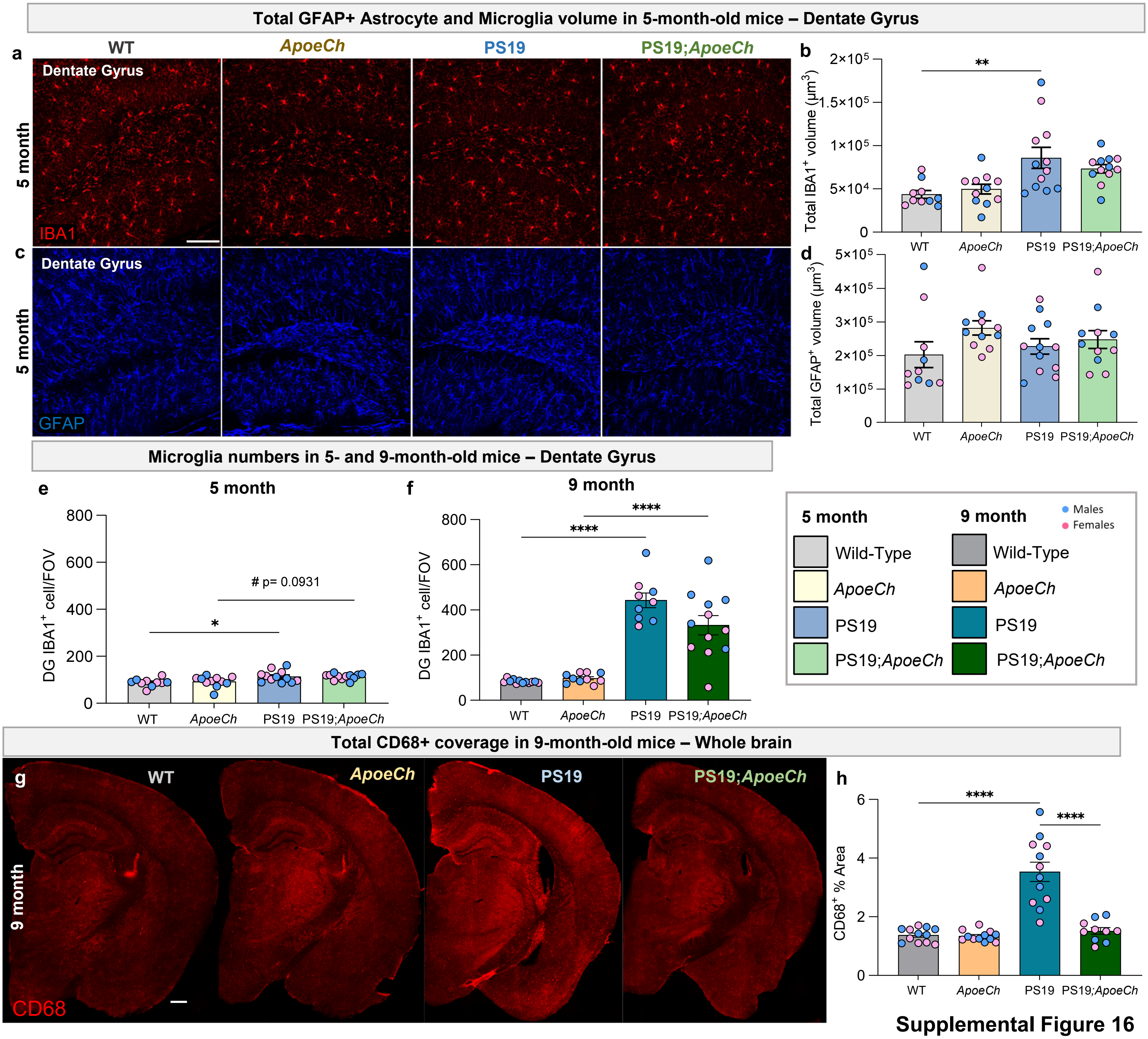

Supplemental Figure 16

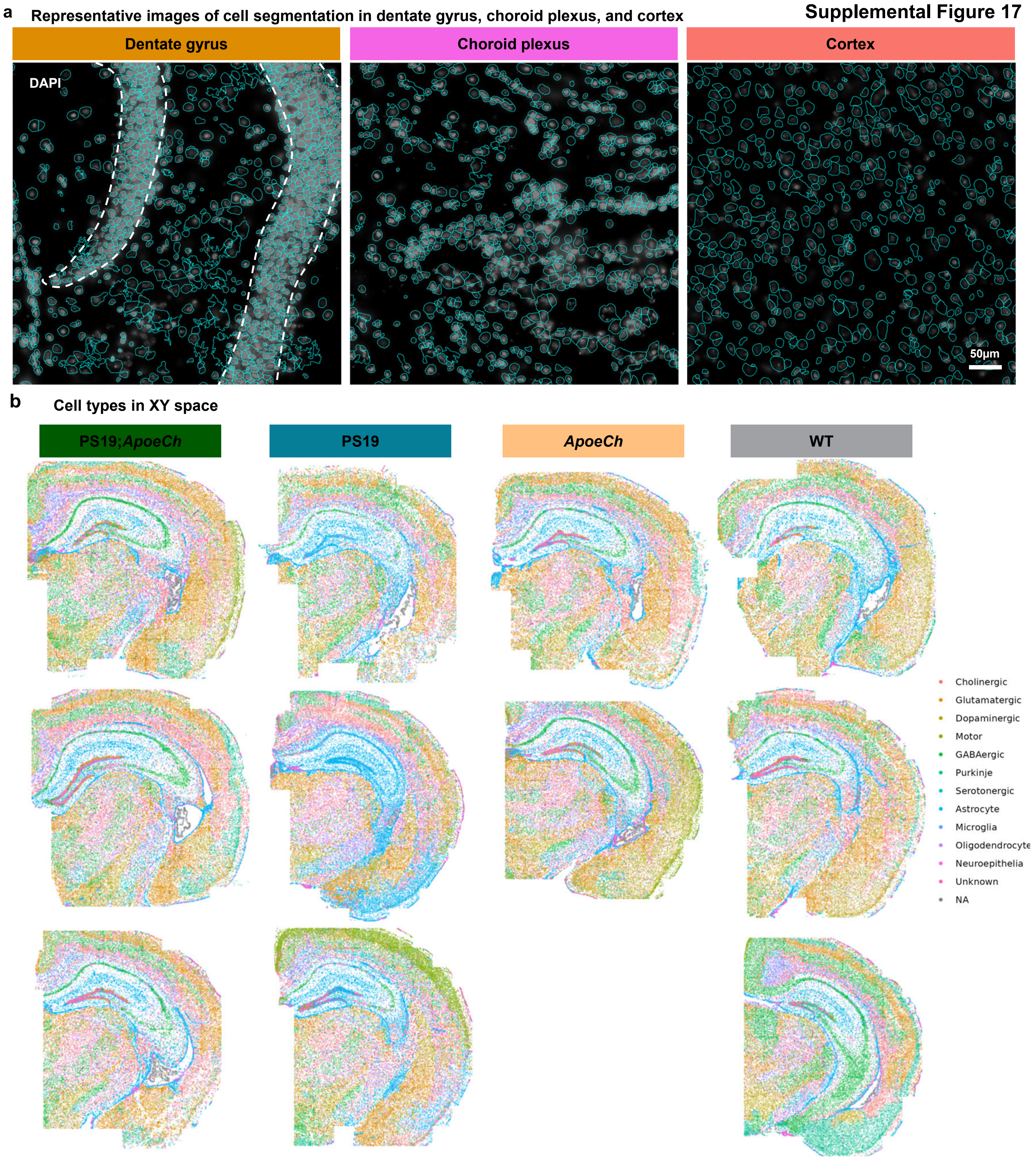
a
Representative images of cell segmentation in dentate gyrus, choroid plexus, and cortex
Dentate gyrus
Choroid plexus
Cortex
DAPI
50µm
b
Cell types in XY space
PS19;ApoeCh
PS19
ApoeCh
WT
Cholinergic
Glutamatergic
Dopaminergic
Motor
GABAergic
Purkinje
Serotonergic
Astrocyte
Microglia
Oligodendrocyte
Neuroepithelia
Unknown
NA

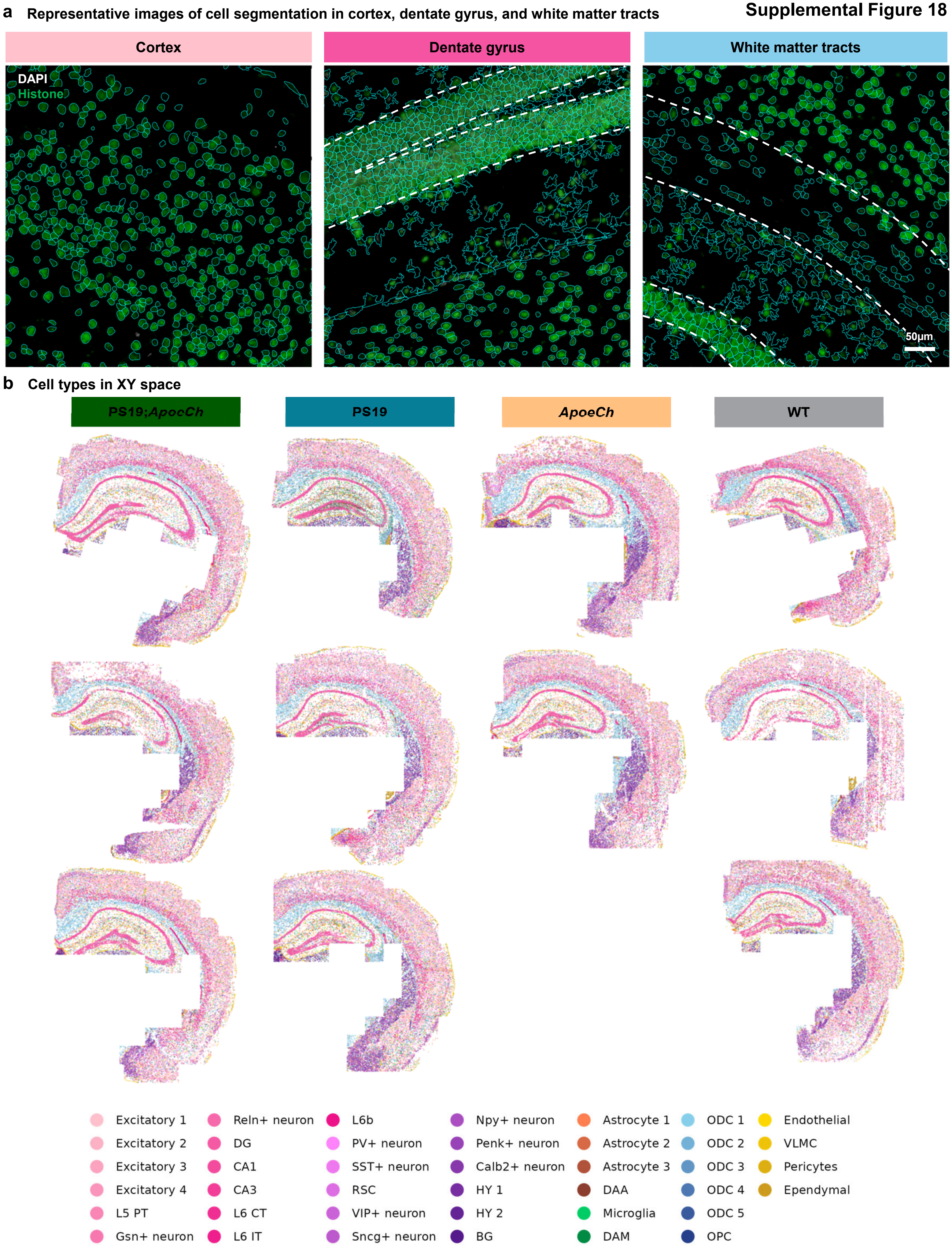
a Representative images of cell segmentation in cortex, dentate gyrus, and white matter tracts
Cortex
Dentate gyrus
White matter tracts
DAPI
Histone
50µm
b Cell types in XY space
PS19;ApocCh
PS19
ApoeCh
WT
Excitatory 1
Excitatory 2
Excitatory 3
Excitatory 4
L5 PT
Gsn+ neuron
Reln+ neuron
DG
CA1
CA3
L6 CT
L6 IT
L6b
PV+ neuron
SST+ neuron
RSC
VIP+ neuron
Sncg+ neuron
Npy+ neuron
Penk+ neuron
Calb2+ neuron
HY 1
HY 2
BG
Astrocyte 1
Astrocyte 2
Astrocyte 3
DAA
Microglia
DAM
ODC 1
ODC 2
ODC 3
ODC 4
ODC 5
OPC
Endothelial
VLMC
Pericytes
Ependymal

Supplemental Figure 19

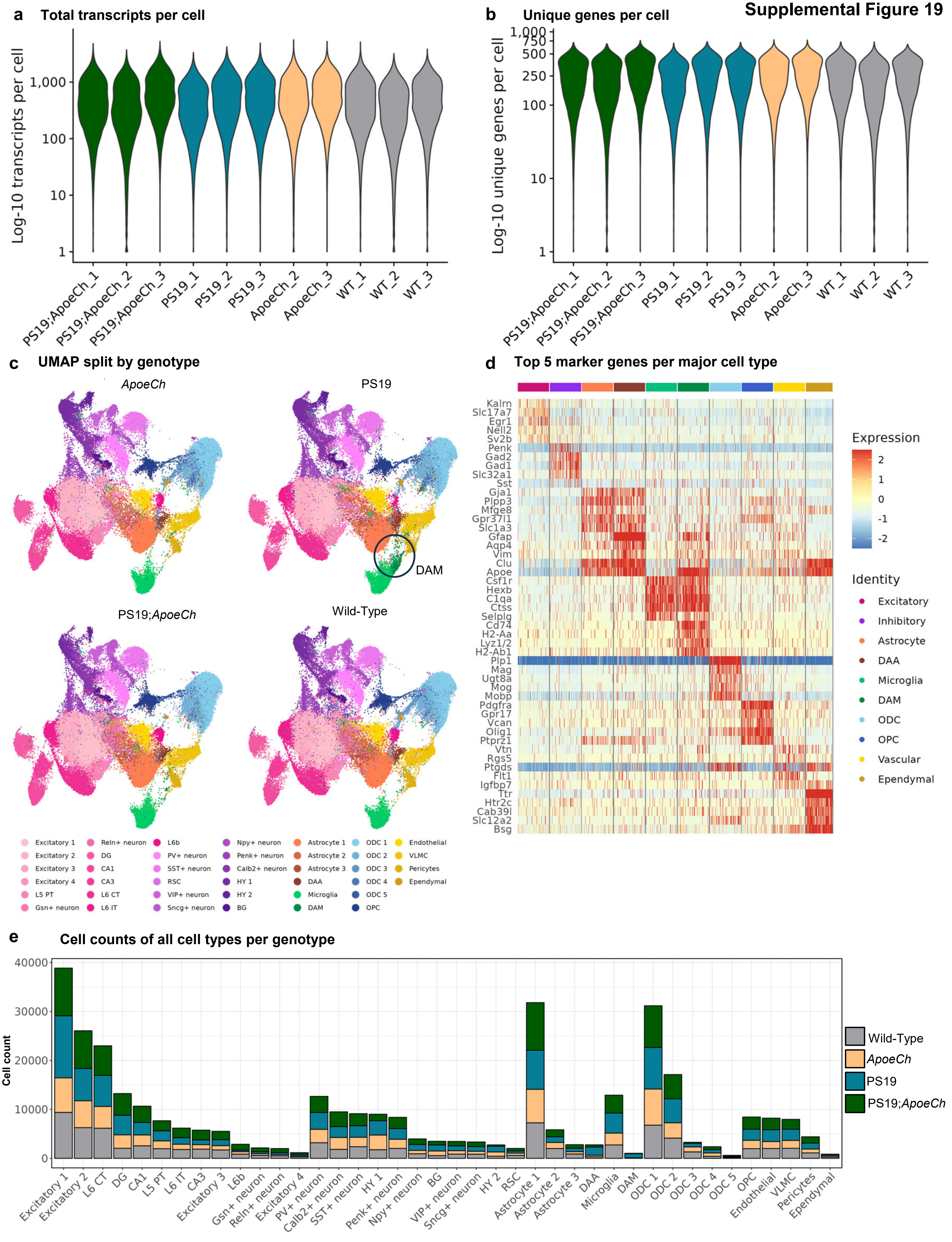

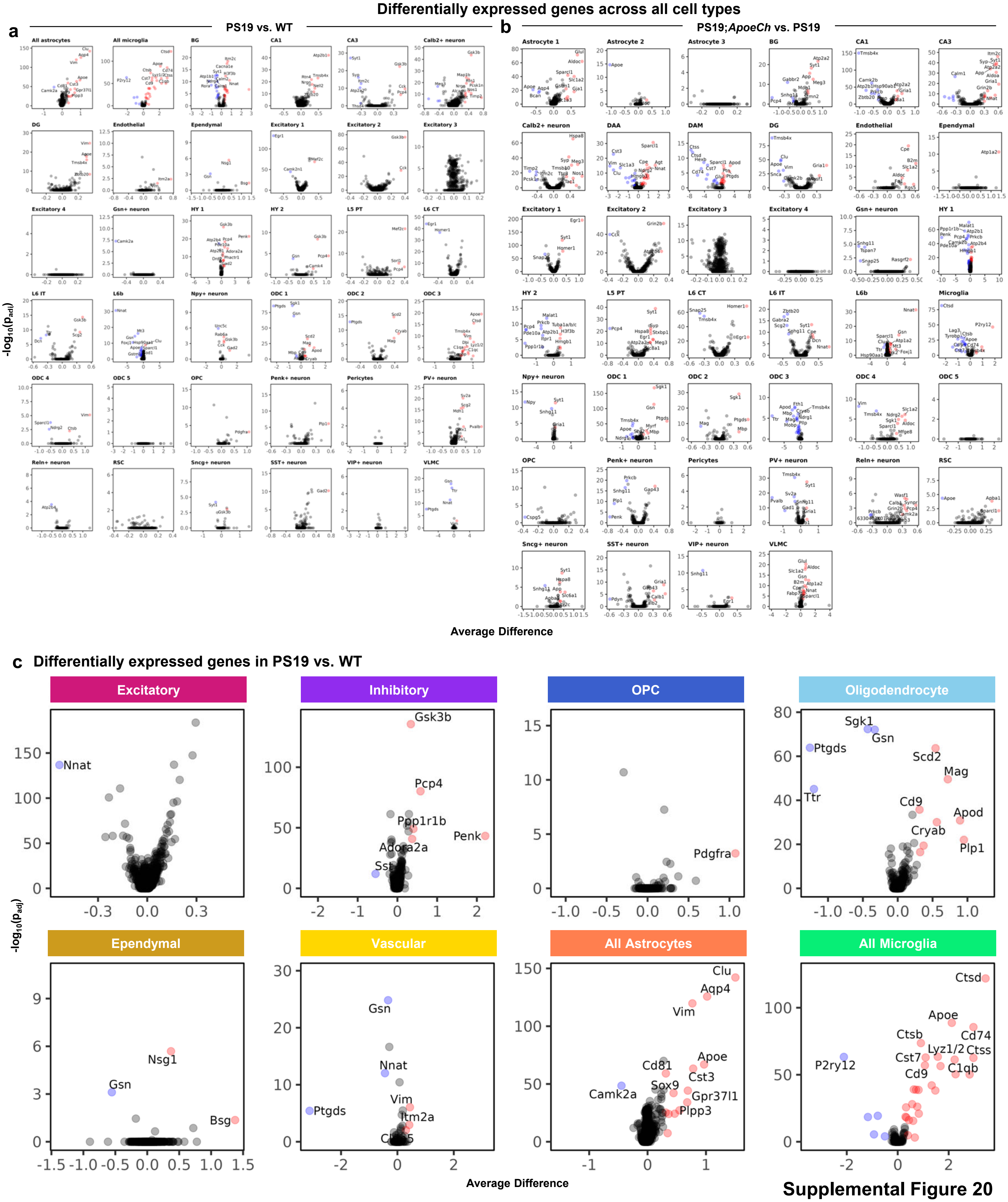

Supplemental Figure 20

Supplemental Figure 21

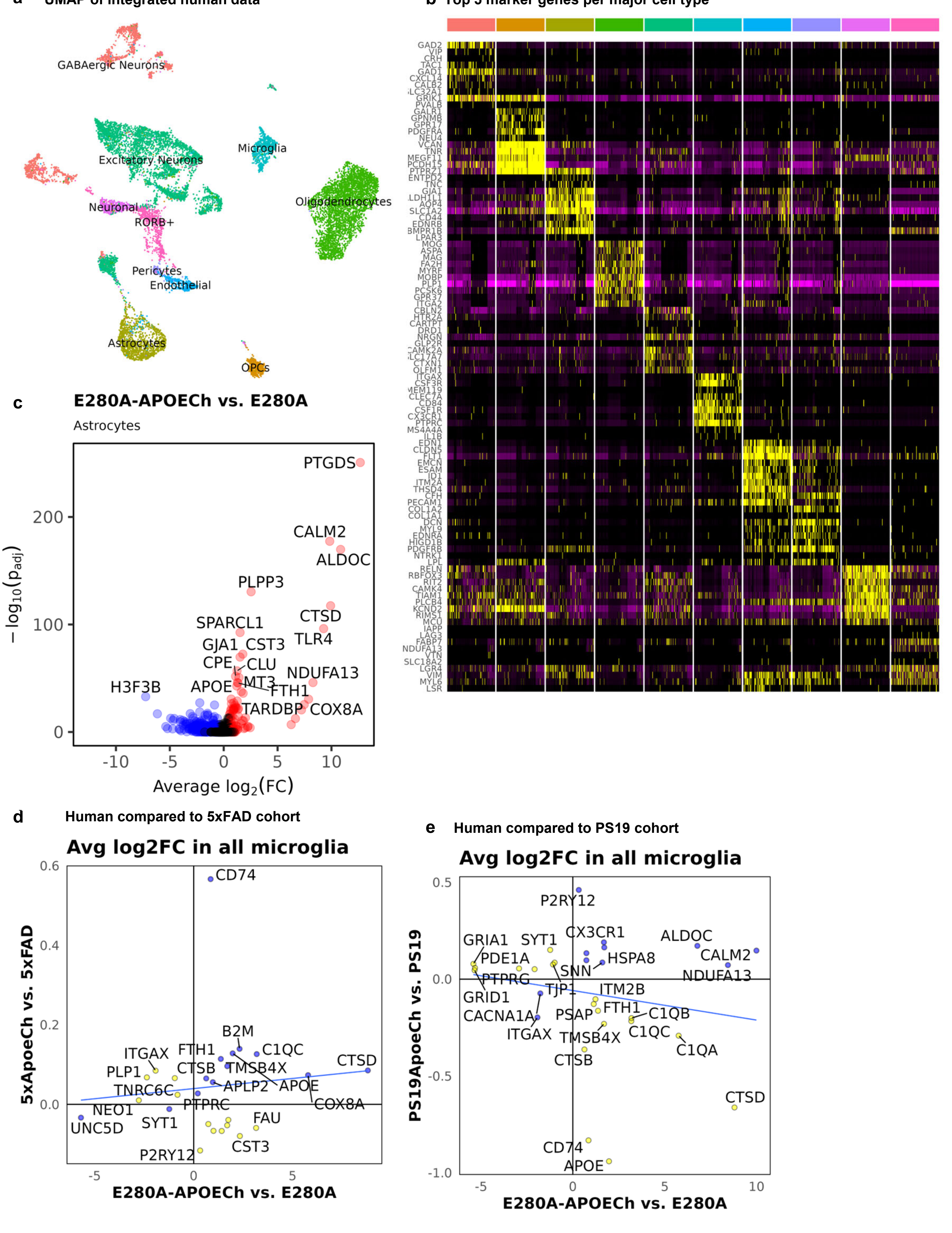

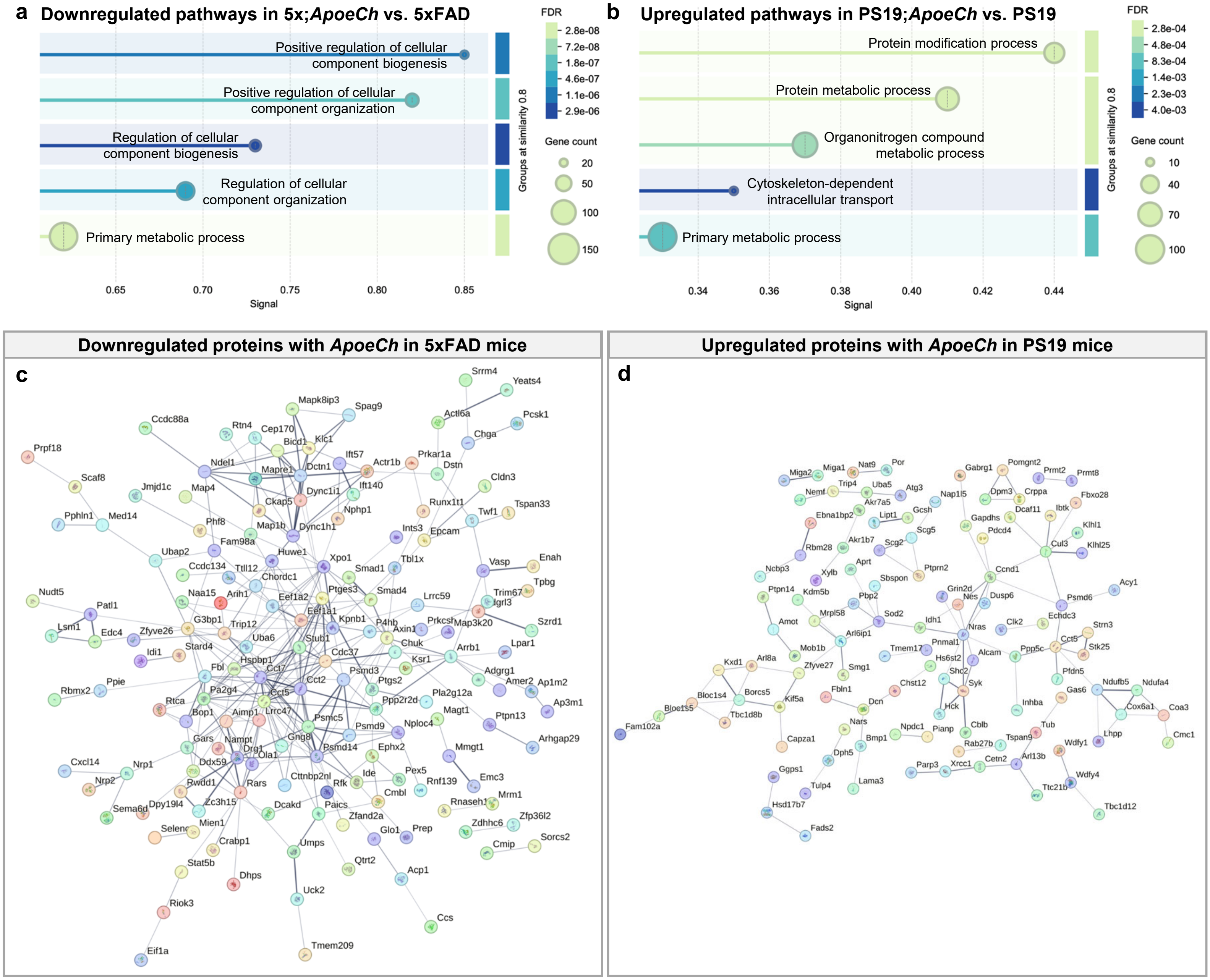

Supplemental Figure 22
